# Supplementary material for: Molecular Characterization of the Enterohemolysin Gene (ehxA) in Clinical Shiga Toxin-Producing Escherichia coli Isolates
Source: Toxins (Basel). 2021 Jan 19;13(1):71. doi: 10.3390/toxins13010071 (PMC7833379; doi:10.3390/toxins13010071)
Supplement: Supplementary file 1 [file toxins-13-00071-s001.pdf]

# Supplementary Materials: Molecular Characterization of the Enterohemolysin Gene (*ehxA*) in Clinical Shiga Toxin-Producing *Escherichia Coli* Isolates

**Ying Hua, Ji Zhang, Cecilia Jernberg, Milan Chromek, Sverker Hansson, Anne Frykman, Yanwen Xiong, Chengsong Wan, Andreas Matussek and Xiangning Bai**

**Table S1.** Association between the presence of *ehxA* and HUS, age of patients, and the duration of bacterial shedding.

| | No. (%) | | | No. (%) | | | No. (%) | | |
|---|---|---|---|---|---|---|---|---|---|
| *ehxA* | HUS (60) | Non-HUS (179) | *p*-value | <10 years of age (104) | ≥10 years of age (80) | *p*-value | Short Shedding (68) | Long Shedding (62) | *p*-value |
| Positive | 53 (88.33) | 146 (81.56) | 0.22 | 87 (83.65) | 62 (77.50) | 0.29 | 59 (86.76) | 49 (79.03) | 0.24 |
| Negative | 7 (11.67) | 33 (18.44) | | 17 (16.35) | 18 (22.50) | | 9 (13.24) | 13 (20.97) | |

[a]: Short shedding: duration of shedding ≤24 days; Long shedding: duration of shedding >24 days.

**Table S2.** Association between *ehxA* subtype C and serotypes.

| *ehxA* Subtype | No. (%) | | *p-value* | No. (%) | | *p-value* |
|---|---|---|---|---|---|---|
| | O121:H19 (25) | Non-O121 (174) | | O26:H11 (35) | Non-O26 (164) | |
| C | 25 (100.00) | 51 (29.31) | <0.001* | 35 (100.00) | 41 (25.00) | <0.001* |
| non-C | 0 (0.00) | 123 (70.69) | | 0 (0.00) | 123 (75.00) | |

* Statistically significant difference.

**Table S3.** Association between the presence of *ehxA* + *eae* + *stx*2 and clinical symptoms or serotypes (.doc).

| *ehxA* + *eae* + *stx*2 | No. (%) | | *p*-value | No. (%) | | *p*-value |
|---|---|---|---|---|---|---|
| | Non-HUS (146) | HUS (53) | | O157:H7 (65) | Non-O157 (134) | |
| + | 47 (32.19) | 47 (88.68) | <0.001* | 56 (86.15) | 38 (28.36) | <0.001* |
| − | 99 (67.81) | 6 (11.32) | | 9 (13.85) | 96 (71.64) | |

* Statistically significant difference.

**Table S4.** Metadata of 239 clinical STEC isolates.

| Strain Name | *ehxA* | *ehxA* Subtype | *ehxA* Genotype | Group | *eae* | *stx* Subtype | Serotype | ST | Symptoms | Age Group | Duration of Bacterial Shedding | Accession Number |
|---|---|---|---|---|---|---|---|---|---|---|---|---|
| SE0301 | + | A | GT1 | I | − | *stx*2d | O91:H21 | 442 | NBS | <10 years | Short | JABKKD000000000 |
| SE0410 | + | A | GT1 | I | − | *stx*2d | O91:H21 | 442 | NBS | ≥10 years | Short | JABKJJ000000000 |
| SE0411 | + | A | GT1 | I | − | *stx*2d | O91:H21 | 442 | NBS | ≥10 years | Short | JABKJI000000000 |

| | | | | | | | | | | | | |
|---|---|---|---|---|---|---|---|---|---|---|---|---|
| SE0701 | + | A | GT2 | I | – | *stx*2b | O113:H4 | 10 | NBS | <10 years | Long | JABKIO000000000 |
| SE0904 | + | A | GT2 | I | – | *stx*2b | O146:H21 | 442 | BD | ≥10 years | Long | JABKHA000000000 |
| SE1212 | + | A | GT2 | I | – | *stx*2b | O150:H10 | 1724 | NBS | <10 years | Short | JABKFJ000000000 |
| SE1214 | + | A | GT2 | I | – | *stx*2b | O150:H10 | 1724 | NBS | <10 years | NA | JABKFH000000000 |
| SE1215 | + | A | GT2 | I | – | *stx*2b | O150:H10 | 1724 | NBS | ≥10 years | NA | JABKFG000000000 |
| SE1216 | + | A | GT2 | I | – | *stx*2b | O150:H10 | 1724 | NBS | ≥10 years | NA | JABKFF000000000 |
| SE0302 | + | A | GT2 | I | – | *stx*1c | O128ab:H2 | 25 | NBS | <10 years | Long | JABKKC000000000 |
| SE1106 | + | A | GT2 | I | – | *stx*1c | O4:H16 | 10 | NBS | ≥10 years | NA | JABKGI000000000 |
| SE1210 | + | A | GT2 | I | – | *stx*1c | O117:H8 | 13 | NBS | ≥10 years | NA | JABKFL000000000 |
| SE0710 | + | A | GT2 | I | – | *stx*1c | O117:H8 | 13 | NBS | ≥10 years | NA | JABKHX000000000 |
| SE0906 | + | A | GT2 | I | – | *stx*1c +*stx*2b | O146:H21 | 442 | NBS | <10 years | Long | JABKGY000000000 |
| SE0307 | + | A | GT2 | I | – | *stx*1c +*stx*2b | O128ab:H2 | 25 | NBS | ≥10 years | NA | JABKJX000000000 |
| SE0813 | + | A | GT2 | I | – | *stx*1a +*stx*2b | O91:H14 | 33 | NBS | ≥10 years | Long | JABKHF000000000 |
| SE0308 | + | A | GT2 | I | – | *stx*1a +*stx*2b | O91:H14 | 33 | NBS | ≥10 years | NA | JABKJW000000000 |
| SE0401 | + | A | GT5 | I | – | *stx*1a +*stx*2a | O185:H28 | 658 | BD | ≥10 years | Short | JABKJS000000000 |
| SE0402 | + | A | GT6 | I | – | *stx*1c | O5:H19 | 447 | NBS | ≥10 years | NA | JABKJR000000000 |
| SE0805 | + | A | GT6 | I | – | *stx*1c +*stx*2b | O113:H4 | 10 | NBS | <10 years | Short | JABKHN000000000 |
| SE0703 | + | A | GT11 | I | – | *stx*1c | Ont:H4 | 3101 | NBS | <10 years | NA | JABKIE000000000 |
| SE0605 | + | A | GT11 | I | – | *stx*1c +*stx*2b | O78:H4 | 3101 | NBS | <10 years | NA | JABKIJ000000000 |
| SE0606 | + | A | GT11 | I | – | *stx*1c +*stx*2b | O78:H4 | 3101 | NBS | ≥10 years | Long | JABKII000000000 |
| SE0804 | + | A | GT12 | I | – | *stx*1a +*stx*2a | O175:H21 | 223 | NBS | <10 years | Long | JABKHO000000000 |
| SE1502 | + | A | GT16 | I | – | *stx*1a +*stx*2a | O183:H18 | 657 | BD | ≥10 years | NA | JABKDZ000000000 |
| SE1115 | + | A | GT16 | I | – | *stx*1a +*stx*2a | O183:H18 | 657 | BD | ≥10 years | Long | JABKFZ000000000 |
| SE1117 | + | A | GT17 | I | – | *stx*2a | O126:H20 | 58 | NBS | ≥10 years | NA | JABKFX000000000 |

| | | | | | | | | | | | | |
|---|---|---|---|---|---|---|---|---|---|---|---|---|
| SE1301 | + | A | GT20 | I | − | *stx*1a +*stx*2d | O163:H19 | 679 | NBS | <10 years | Long | JABKFE000000000 |
| SE1310 | + | A | GT20 | I | − | *stx*1a +*stx*2d | O163:H19 | 679 | NBS | ≥10 years | Short | JABKEU000000000 |
| SE1201 | + | B | GT3 | II | + | *stx*2a +*stx*2c | O157:H7 | 11 | BD | ≥10 years | NA | JABKFU000000000 |
| SE0903 | + | B | GT3 | II | + | *stx*2a +*stx*2c | O157:H7 | 11 | BD | <10 years | Long | JABKHB000000000 |
| SE0506 | + | B | GT3 | II | + | *stx*2a +*stx*2c | O157:H7 | 11 | BD | <10 years | Short | JABKJB000000000 |
| SE0407 | + | B | GT3 | II | + | *stx*2a +*stx*2c | O157:H7 | 11 | BD | <10 years | Long | JABKJM000000000 |
| SE0303 | + | B | GT3 | II | + | *stx*2a +*stx*2c | O157:H7 | 11 | BD | <10 years | Long | JABKKB000000000 |
| SE0510 | + | B | GT3 | II | + | *stx*2a +*stx*2c | O157:H7 | 11 | BD | ≥10 years | Short | JABKIY000000000 |
| SE0512 | + | B | GT3 | II | + | *stx*2a +*stx*2c | O157:H7 | 11 | BD | ≥10 years | Short | JABKIW000000000 |
| SE0513 | + | B | GT3 | II | + | *stx*2a +*stx*2c | O157:H7 | 11 | BD | ≥10 years | Short | JABKIV000000000 |
| SE0514 | + | B | GT3 | II | + | *stx*2a +*stx*2c | O157:H7 | 11 | NBS | ≥10 years | Short | JABKIU000000000 |
| SE0515 | + | B | GT3 | II | + | *stx*2a +*stx*2c | O157:H7 | 11 | HUS | <10 years | Long | JABKIT000000000 |
| SE1315 | + | B | GT3 | II | + | *stx*2a +*stx*2c | O157:H7 | 11 | BD | ≥10 years | NA | JABKEP000000000 |
| SE0412 | + | B | GT3 | II | + | *stx*2a +*stx*2c | O157:H7 | 11 | BD | ≥10 years | NA | JABKJH000000000 |
| SE0715 | + | B | GT3 | II | + | *stx*2a +*stx*2c | O157:H7 | 11 | BD | ≥10 years | NA | JABKHU000000000 |
| SE0309 | + | B | GT3 | II | + | *stx*2a +*stx*2c | O157:H7 | 11 | NBS | <10 years | Short | JABKJV000000000 |
| SE0310 | + | B | GT3 | II | + | *stx*2a +*stx*2c | O157:H7 | 11 | NBS | <10 years | Short | JABKJU000000000 |
| SE0311 | + | B | GT3 | II | + | *stx*2a +*stx*2c | O157:H7 | 11 | NBS | ≥10 years | Short | JABKJT000000000 |
| SEH9701 | + | B | GT3 | II | + | *stx*2a +*stx*2c | O157:H7 | 11 | HUS | NA | NA | JABWEJ000000000 |
| SEH9702 | + | B | GT3 | II | + | *stx*2a +*stx*2c | O157:H7 | 11 | HUS | NA | NA | JABWEK000000000 |
| SEH0501 | + | B | GT3 | II | + | *stx*2a +*stx*2c | O157:H7 | 11 | HUS | NA | NA | JABWEX000000000 |
| SEH0502 | + | B | GT3 | II | + | *stx*2a +*stx*2c | O157:H7 | 11 | HUS | NA | NA | JABWEY000000000 |
| SEH9703 | + | B | GT3 | II | + | *stx*2a +*stx*2c | O157:H7 | 11 | HUS | NA | NA | JABWEL000000000 |
| SEH0602 | + | B | GT3 | II | + | *stx*2a +*stx*2c | O157:H7 | 11 | HUS | NA | NA | JABWFG000000000 |

| | | | | | | | | | | | | |
|---|---|---|---|---|---|---|---|---|---|---|---|---|
| SEH1005 | + | B | GT3 | II | + | *stx*2a +*stx*2c | O157:H7 | 11 | HUS | NA | NA | JABWFQ000000000 |
| SEH9704 | + | B | GT3 | II | + | *stx*2a +*stx*2c | O157:H7 | 11 | HUS | NA | NA | JABWEM000000000 |
| SEH0505 | + | B | GT3 | II | + | *stx*2a +*stx*2c | O157:H7 | 11 | HUS | NA | NA | JABWFB000000000 |
| SEH0302 | + | B | GT3 | II | + | *stx*2a +*stx*2c | O157:H7 | 11 | HUS | NA | NA | JABWES000000000 |
| SEH0506 | + | B | GT3 | II | + | *stx*2a +*stx*2c | O157:H7 | 11 | HUS | NA | NA | JABWFC000000000 |
| SEH0202 | + | B | GT3 | II | + | *stx*2a +*stx*2c | O157:H7 | 11 | HUS | NA | NA | JABWEQ000000000 |
| SEH0401 | + | B | GT3 | II | + | *stx*2a +*stx*2c | O157:H7 | 11 | HUS | NA | NA | JABWET000000000 |
| SEH0603 | + | B | GT3 | II | + | *stx*2a +*stx*2c | O157:H7 | 11 | HUS | NA | NA | JABWFH000000000 |
| SEH0901 | + | B | GT3 | II | + | *stx*2a +*stx*2c | O157:H7 | 11 | HUS | NA | NA | JABWFL000000000 |
| SEH1001 | + | B | GT3 | II | + | *stx*2a +*stx*2c | O157:H7 | 11 | HUS | NA | NA | JABWFM000000000 |
| SEH1102 | + | B | GT3 | II | + | *stx*2a +*stx*2c | O157:H7 | 11 | HUS | NA | NA | JABWFS000000000 |
| SEH1406 | + | B | GT3 | II | + | *stx*2a +*stx*2c | O157:H7 | 11 | HUS | NA | NA | JABWGC000000000 |
| SEH1701 | + | B | GT3 | II | + | *stx*2a +*stx*2c | O157:H7 | 11 | HUS | NA | NA | JABWGG000000000 |
| SEH1601 | + | B | GT3 | II | + | *stx*2a +*stx*2c | O157:H7 | 11 | HUS | NA | NA | JABWGF000000000 |
| SEH1801 | + | B | GT3 | II | + | *stx*2a +*stx*2c | O157:H7 | 11 | HUS | NA | NA | JABWGH000000000 |
| SEH1802 | + | B | GT3 | II | + | *stx*2a +*stx*2c | O157:H7 | 11 | HUS | NA | NA | JABWGI000000000 |
| SEH0503 | + | B | GT3 | II | + | *stx*2a +*stx*2c | O157:H7 | 11 | HUS | NA | NA | JABWEZ000000000 |
| SEH9501 | + | B | GT3 | II | + | *stx*2a +*stx*2c | O157:H7 | 11 | HUS | NA | NA | JABWEI000000000 |
| SEH0701 | + | B | GT3 | II | + | *stx*2a +*stx*2c | O157:H7 | 11 | HUS | NA | NA | JABWFI000000000 |
| SEH0402 | + | B | GT3 | II | + | *stx*2a +*stx*2c | O157:H7 | 11 | HUS | NA | NA | JABWEU000000000 |
| SEH9901 | + | B | GT3 | II | + | *stx*2c | O157:H7 | 11 | HUS | NA | NA | JABWEO000000000 |
| SEH0507 | + | B | GT3 | II | + | *stx*2c | O157:H7 | 11 | HUS | NA | NA | JABWFD000000000 |
| SEH0508 | + | B | GT3 | II | + | *stx*2a | O157:H7 | 11 | HUS | NA | NA | JABWFE000000000 |
| SEH0403 | + | B | GT3 | II | + | *stx*2a | O157:H7 | 11 | HUS | NA | NA | JABWEV000000000 |

| | | | | | | | | | | | | |
|---|---|---|---|---|---|---|---|---|---|---|---|---|
| SE1119 | + | B | GT18 | II | + | *stx*2c | O157:H7 | 11 | NBS | <10 years | Short | JABKFV000000000 |
| SE0803 | + | B | GT29 | II | + | *stx*2c | O157:H7 | 11 | BD | <10 years | Short | JABKHP000000000 |
| SE0404 | + | B | GT29 | II | + | *stx*2c | O157:H7 | 11 | NBS | <10 years | Long | JABKJP000000000 |
| SE1704 | + | B | GT29 | II | + | *stx*2c | O157:H7 | 1595 | NBS | <10 years | Short | JABKDK000000000 |
| SE0808 | + | B | GT29 | II | + | *stx*2c | O157:H7 | 11 | NBS | <10 years | Short | JABKHK000000000 |
| SE0809 | + | B | GT29 | II | + | *stx*2c | O157:H7 | 11 | NBS | <10 years | Short | JABKHJ000000000 |
| SE0909 | + | B | GT29 | II | + | *stx*2c | O157:H7 | 1804 | NBS | <10 years | Long | JABKGW000000000 |
| SE1208 | + | B | GT29 | II | + | *stx*1a +*stx*2c | O157:H7 | 7645 | BD | ≥10 years | NA | JABKFN000000000 |
| SE0604 | + | B | GT29 | II | + | *stx*1a +*stx*2c | O157:H7 | 1804 | BD | ≥10 years | Short | JABKIK000000000 |
| SE0503 | + | B | GT29 | II | + | *stx*1a +*stx*2c | O157:H7 | 11 | BD | <10 years | Short | JABKJE000000000 |
| SE1302 | + | B | GT29 | II | + | *stx*1a +*stx*2c | O157:H7 | 11 | NBS | <10 years | Short | JABKFD000000000 |
| SE1303 | + | B | GT29 | II | + | *stx*1a +*stx*2c | O157:H7 | 11 | NBS | <10 years | Long | JABKFC000000000 |
| SE1204 | + | B | GT29 | II | + | *stx*1a +*stx*2c | O157:H7 | 11 | BD | ≥10 years | NA | JABKFR000000000 |
| SE0304 | + | B | GT29 | II | + | *stx*1a +*stx*2a | O157:H7 | 11 | BD | ≥10 years | NA | JABKKA000000000 |
| SE0708 | + | B | GT29 | II | + | *stx*1a +*stx*2c | O157:H7 | 11 | BD | <10 years | Long | JABKHZ000000000 |
| SE0907 | + | B | GT29 | II | + | *stx*1a +*stx*2c | O157:H7 | 11 | BD | ≥10 years | Short | JABKGX000000000 |
| SE1308 | + | B | GT29 | II | + | *stx*2a +*stx*2c | O157:H7 | 11 | NBS | ≥10 years | NA | JABKEW000000000 |
| SE1309 | + | B | GT29 | II | + | *stx*2a +*stx*2c | O157:H7 | 11 | NBS | ≥10 years | NA | JABKEV000000000 |
| SEH0201 | + | B | GT29 | II | + | *stx*2a +*stx*2c | O157:H7 | 11 | HUS | NA | NA | JABWEP000000000 |
| SE0902 | + | C | GT7 | II | + | *stx*1a | O84:H2 | 306 | NBS | <10 years | Long | JABKHC000000000 |
| SE0408 | + | C | GT7 | II | + | *stx*1a | O98:H21 | 306 | HUS | ≥10 years | Short | JABKJL000000000 |
| SEH9705 | + | C | GT8 | II | + | *stx*2a | O121:H19 | 655 | HUS | NA | NA | JABWEN000000000 |
| SEH1002 | + | C | GT8 | II | + | *stx*2a | O121:H19 | 655 | HUS | NA | NA | JABWFN000000000 |
| SE1001 | + | C | GT8 | II | + | *stx*2a | O121:H19 | 655 | NBS | <10 years | Short | JABKGV000000000 |

| | | | | | | | | | | | | |
|---|---|---|---|---|---|---|---|---|---|---|---|---|
| SE1401 | + | C | GT8 | II | + | *stx*2a | O121:H19 | 655 | BD | <10 years | Long | JABKEK000000000 |
| SE1402 | + | C | GT8 | II | + | *stx*2a | O121:H19 | 655 | BD | ≥10 years | Long | JABKEJ000000000 |
| SE1403 | + | C | GT8 | II | + | *stx*2a | O121:H19 | 655 | NBS | <10 years | NA | JABKEI000000000 |
| SE1705 | + | C | GT8 | II | + | *stx*2a | O121:H19 | 655 | NBS | <10 years | Long | JABKDJ000000000 |
| SE1503 | + | C | GT8 | II | + | *stx*2a | O121:H19 | 655 | HUS | <10 years | NA | JABKDY000000000 |
| SE1005 | + | C | GT8 | II | + | *stx*2a | O121:H19 | 655 | BD | <10 years | Short | JABKGR000000000 |
| SE1108 | + | C | GT8 | II | + | *stx*2a | O121:H19 | 655 | NBS | ≥10 years | NA | JABKGG000000000 |
| SE0603 | + | C | GT8 | II | + | *stx*2a | O121:H19 | 655 | BD | ≥10 years | Short | JABKIL000000000 |
| SE1707 | + | C | GT8 | II | + | *stx*2a | O121:H19 | 655 | BD | <10 years | NA | JABKDH000000000 |
| SE0711 | + | C | GT8 | II | + | *stx*2a | O121:H19 | 655 | BD | ≥10 years | Short | JABKHW000000000 |
| SE0712 | + | C | GT8 | II | + | *stx*2a | O121:H19 | 655 | NBS | <10 years | Long | JABKIH000000000 |
| SE0814 | + | C | GT8 | II | + | *stx*2a | O121:H19 | 655 | BD | <10 years | NA | JABKHE000000000 |
| SE1606 | + | C | GT8 | II | + | *stx*2a | O121:H19 | 655 | BD | <10 years | Long | JABKDP000000000 |
| SEH0601 | + | C | GT8 | II | + | *stx*2a | O121:H19 | 655 | HUS | NA | NA | JABWFF000000000 |
| SEH1301 | + | C | GT8 | II | + | *stx*2a | O121:H19 | 655 | HUS | NA | NA | JABWFW000000000 |
| SEH1404 | + | C | GT8 | II | + | *stx*2a | O121:H19 | 655 | HUS | NA | NA | JABWGA000000000 |
| SEH1202 | + | C | GT8 | II | + | *stx*2a | O121:H19 | 655 | HUS | NA | NA | JABWFU000000000 |
| SEH0301 | + | C | GT8 | II | + | *stx*2a | O121:H19 | 655 | HUS | NA | NA | JABWER000000000 |
| SEH1402 | + | C | GT8 | II | + | *stx*2a | O121:H19 | 655 | HUS | NA | NA | JABWFY000000000 |
| SEH9401 | + | C | GT8 | II | − | *stx*2a | O121:H19 | 655 | HUS | NA | NA | JABWEH000000000 |
| SE0501 | + | C | GT8 | II | + | *stx*1a +*stx*2a | O121:H19 | 655 | NBS | ≥10 years | Short | JABKJG000000000 |
| SEH0504 | + | C | GT8 | II | + | *stx*1a +*stx*2a | O121:H19 | 655 | HUS | NA | NA | JABWFA000000000 |
| SE0508 | + | C | GT9 | II | + | *stx*1a | O26:H11 | 21 | NBS | <10 years | Long | JABKJA000000000 |
| SE0901 | + | C | GT13 | II | + | *stx*1a | O111:H8 | N[a] | NBS | <10 years | Short | JABKHS000000000 |

| | | | | | | | | | | | | |
|---|---|---|---|---|---|---|---|---|---|---|---|---|
| SE1007 | + | C | GT14 | II | + | $stx_{2a}$ | O26:H11 | 29 | NBS | <10 years | Long | JABKGP000000000 |
| SE1008 | + | C | GT14 | II | + | $stx_{1a}$ | O26:H11 | 29 | NBS | <10 years | Short | JABKGO000000000 |
| SE1107 | + | C | GT15 | II | + | $stx_{2a}$ | O180:H2 | 301 | NBS | ≥10 years | NA | JABKGH000000000 |
| SE1205 | + | C | GT19 | II | + | $stx_{2a}$ +$stx_{2c}$ | O165:H25 | 119 | BD | ≥10 years | NA | JABKFQ000000000 |
| SEH0801 | + | C | GT21 | II | + | $stx_{1a}$ +$stx_{2a}$ | O111:H8 | 16 | HUS | NA | NA | JABWFK000000000 |
| SE1313 | + | C | GT21 | II | + | $stx_{1a}$ | O111:H8 | 16 | BD | <10 years | Short | JABKER000000000 |
| SE1316 | + | C | GT21 | II | + | $stx_{1a}$ | O111:H8 | 16 | NBS | <10 years | Short | JABKEO000000000 |
| SE1404 | + | C | GT22 | II | + | $stx_{2a}$ +$stx_{2c}$ | O165:H25 | 119 | NBS | <10 years | Long | JABKEH000000000 |
| SEH1405 | + | C | GT22 | II | + | $stx_{2a}$ +$stx_{2c}$ | O165:H25 | 119 | HUS | NA | NA | JABWGB000000000 |
| SEH1501 | + | C | GT22 | II | + | $stx_{2a}$ | O165:H25 | 119 | HUS | NA | NA | JABWGE000000000 |
| SE1406 | + | C | GT23 | II | + | $stx_{2a}$ | O145:H28 | 137 | NBS | ≥10 years | NA | JABKEG000000000 |
| SEH0702 | + | C | GT28 | II | + | $stx_{2a}$ | O103:H8 | 2836 | HUS | NA | NA | JABWFJ000000000 |
| SE0714 | + | C | GT30 | II | + | $stx_{1a}$ | O145:H28 | 32 | NBS | <10 years | Short | JABKHV000000000 |
| SEH1101 | + | C | GT30 | II | + | $stx_{1a}$ | O26:H11 | 21 | HUS | NA | NA | JABWFR000000000 |
| SEH1003 | + | C | GT30 | II | + | $stx_{2a}$ | O145:H28 | 32 | HUS | NA | NA | JABWFO000000000 |
| SEH1403 | + | C | GT30 | II | + | $stx_{1a}$ | O103:H2 | 386 | HUS | NA | NA | JABWFZ000000000 |
| SE0504 | + | C | GT30 | II | + | $stx_{1a}$ | O26:H11 | 21 | NBS | <10 years | Long | JABKJD000000000 |
| SE0702 | + | C | GT30 | II | + | $stx_{1a}$ | O26:H11 | 21 | BD | ≥10 years | Long | JABKIF000000000 |
| SE1002 | + | C | GT30 | II | + | $stx_{1a}$ | O26:H11 | 21 | NBS | ≥10 years | Short | JABKGU000000000 |
| SE1701 | + | C | GT30 | II | + | $stx_{1a}$ | O26:H11 | 21 | BD | ≥10 years | Short | JABKDN000000000 |
| SE0505 | + | C | GT30 | II | + | $stx_{1a}$ | O26:H11 | 21 | NBS | <10 years | NA | JABKJC000000000 |
| SE1703 | + | C | GT30 | II | + | $stx_{1a}$ | O26:H11 | 21 | NBS | <10 years | NA | JABKDL000000000 |
| SE1203 | + | C | GT30 | II | + | $stx_{1a}$ | O26:H11 | 21 | BD | ≥10 years | Short | JABKFS000000000 |
| SE0705 | + | C | GT30 | II | + | $stx_{1a}$ | O26:H11 | 21 | NBS | <10 years | Short | JABKIC000000000 |

| | | | | | | | | | | | | |
|---|---|---|---|---|---|---|---|---|---|---|---|---|
| SE1405 | + | C | GT30 | II | + | *stx*1a | O26:H11 | 21 | BD | <10 years | Short | JABKEZ000000000 |
| SE1504 | + | C | GT30 | II | + | *stx*1a | O26:H11 | 21 | NBS | <10 years | Long | JABKDX000000000 |
| SE0806 | + | C | GT30 | II | + | *stx*1a | O26:H11 | 21 | NBS | <10 years | Long | JABKHM000000000 |
| SE1110 | + | C | GT30 | II | + | *stx*1a | O26:H11 | 21 | NBS | ≥10 years | NA | JABKGE000000000 |
| SE1111 | + | C | GT30 | II | + | *stx*1a | O26:H11 | 21 | NBS | <10 years | Short | JABKGD000000000 |
| SE1209 | + | C | GT30 | II | + | *stx*1a | O26:H11 | 21 | BD | ≥10 years | NA | JABKFM000000000 |
| SE0511 | + | C | GT30 | II | + | *stx*1a | O26:H11 | 21 | NBS | <10 years | Short | JABKIX000000000 |
| SE1408 | + | C | GT30 | II | + | *stx*1a | O26:H11 | 21 | BD | <10 years | Long | JABKEF000000000 |
| SE0810 | + | C | GT30 | II | + | *stx*1a | O26:H11 | 21 | NBS | <10 years | Short | JABKHI000000000 |
| SE1409 | + | C | GT30 | II | + | *stx*1a | O26:H11 | 21 | BD | ≥10 years | NA | JABKEE000000000 |
| SE1113 | + | C | GT30 | II | + | *stx*1a | O26:H11 | 21 | NBS | <10 years | Short | JABKGB000000000 |
| SE1410 | + | C | GT30 | II | + | *stx*1a | O26:H11 | 21 | NBS | <10 years | Long | JABKED000000000 |
| SE1411 | + | C | GT30 | II | + | *stx*1a | O26:H11 | 21 | NBS | ≥10 years | Long | JABKEC000000000 |
| SE1116 | + | C | GT30 | II | + | *stx*2a | O26:H11 | 21 | NBS | <10 years | Long | JABKFY000000000 |
| SE1312 | + | C | GT30 | II | + | *stx*1a | O26:H11 | 21 | BD | ≥10 years | NA | JABKES000000000 |
| SE1314 | + | C | GT30 | II | + | *stx*1a | O26:H11 | 21 | NBS | <10 years | Long | JABKEQ000000000 |
| SE1608 | + | C | GT30 | II | − | *stx*1a | O26:H11 | 21 | NBS | ≥10 years | Short | JABKDO000000000 |
| SE0716 | + | C | GT30 | II | + | *stx*1a | O26:H11 | 21 | NBS | <10 years | Long | JABKHT000000000 |
| SE0519 | + | C | GT30 | II | + | *stx*1a | O26:H11 | 21 | NBS | <10 years | NA | JABKIP000000000 |
| SE1213 | + | C | GT30 | II | + | *stx*2a | O26:H11 | 21 | BD | ≥10 years | NA | JABKFI000000000 |
| SEH0404 | + | C | GT30 | II | + | *stx*2a | O26:H11 | 21 | HUS | NA | NA | JABWEW000000000 |
| SEH1407 | + | C | GT30 | II | + | *stx*2a | O26:H11 | 21 | HUS | NA | NA | JABWGD000000000 |
| SE1702 | + | C | GT30 | II | + | *stx*1a +*stx*2a | O26:H11 | 21 | BD | ≥10 years | NA | JABKDM000000000 |
| SE572 | + | D | GT26 | III | − | *stx*2g | O187:H28 | 200 | NBS | <10 years | Short | SAMN09758572 |

| | | | | | | | | | | | | |
|---|---|---|---|---|---|---|---|---|---|---|---|---|
| SE574 | + | D | GT27 | III | − | *stx*2a | O136:H12 | 329 | NBS | ≥10 years | Short | SAMN09758574 |
| SE0502 | + | F | GT4 | II | + | *stx*1a | O103:H2 | 17 | NBS | <10 years | Long | JABKJF000000000 |
| SE1202 | + | F | GT4 | II | + | *stx*1a | O103:H2 | 17 | NBS | <10 years | Short | JABKFT000000000 |
| SE1501 | + | F | GT4 | II | + | *stx*1a | O103:H2 | 17 | NBS | <10 years | NA | JABKEA000000000 |
| SE0801 | + | F | GT4 | II | + | *stx*1a | O103:H2 | 17 | NBS | <10 years | Long | JABKHR000000000 |
| SE0802 | + | F | GT4 | II | + | *stx*1a | O103:H2 | 17 | NBS | <10 years | Short | JABKHQ000000000 |
| SE1304 | + | F | GT4 | II | + | *stx*1a | O103:H2 | 17 | BD | ≥10 years | Long | JABKFB000000000 |
| SE1305 | + | F | GT4 | II | + | *stx*1a | O103:H2 | 17 | NBS | ≥10 years | Short | JABKFA000000000 |
| SE1004 | + | F | GT4 | II | + | *stx*1a | O103:H2 | 17 | NBS | <10 years | Long | JABKGS000000000 |
| SE1006 | + | F | GT4 | II | + | *stx*1a | O103:H2 | 17 | NBS | ≥10 years | Short | JABKGQ000000000 |
| SE0509 | + | F | GT4 | II | + | *stx*1a | O103:H2 | 17 | NBS | ≥10 years | Long | JABKIZ000000000 |
| SE1109 | + | F | GT4 | II | + | *stx*1a | O123:H2 | 17 | NBS | <10 years | Long | JABKGF000000000 |
| SE0305 | + | F | GT4 | II | + | *stx*1a | O103:H2 | 17 | BD | ≥10 years | Short | JABKJZ000000000 |
| SE0807 | + | F | GT4 | II | + | *stx*1a | O103:H2 | 17 | BD | <10 years | Long | JABKHL000000000 |
| SE1603 | + | F | GT4 | II | + | *stx*1a | O103:H2 | 17 | NBS | <10 years | Short | JABKDS000000000 |
| SE1505 | + | F | GT4 | II | + | *stx*1a | O103:H2 | 17 | NBS | <10 years | Long | JABKDW000000000 |
| SE1112 | + | F | GT4 | II | + | *stx*1a | O123:H2 | 17 | NBS | <10 years | Long | JABKGC000000000 |
| SE1317 | + | F | GT4 | II | + | *stx*1a | O123:H2 | 17 | NBS | <10 years | Long | JABKEN000000000 |
| SE1118 | + | F | GT4 | II | + | *stx*1a | O103:H2 | 17 | NBS | <10 years | Short | JABKFW000000000 |
| SE1318 | + | F | GT4 | II | + | *stx*1a | O71:H2 | 17 | NBS | <10 years | Short | JABKEM000000000 |
| SE1413 | + | F | GT4 | II | + | *stx*1a | O103:H2 | 17 | BD | <10 years | Long | JABKEB000000000 |
| SE0516 | + | F | GT10 | II | + | *stx*2c | O177:H25 | 342 | NBS | <10 years | Short | JABKIS000000000 |
| SE0517 | + | F | GT10 | II | + | *stx*2c | O177:H25 | 342 | NBS | <10 years | Short | JABKIR000000000 |
| SE0518 | + | F | GT10 | II | + | *stx*2c | O177:H25 | 342 | NBS | <10 years | Long | JABKIQ000000000 |

| | | | | | | | | | | | |
|---|---|---|---|---|---|---|---|---|---|---|---|
| SE1607 | + | F | GT24 | II | + | $stx_{1a}$ | O103:H2 | 17 | NBS | <10 years | NA | JABKDV000000000 |
| SE1602 | + | F | GT24 | II | + | $stx_{1a}$ | O103:H2 | 401 | NBS | ≥10 years | Short | JABKDT000000000 |
| SE1706 | + | F | GT25 | II | + | $stx_{1a}$ | O5:H9 | 342 | NBS | <10 years | Long | JABKDI000000000 |
| SE1604 | + | F | GT25 | II | + | $stx_{1a}$ | O5:H9 | 342 | NBS | <10 years | Short | JABKDR000000000 |
| SE0713 | − | - | - | - | + | $stx_{2a}$ | O121:H19 | 655 | NBS | ≥10 years | Long | JABKIG000000000 |
| SEH1201 | − | - | - | - | + | $stx_{2a}$ | O111:H8 | 327 | HUS | NA | NA | JABWFT000000000 |
| SE0601 | − | - | - | - | + | $stx_{1a}$ | O26:H11 | 21 | NBS | <10 years | Long | JABKIN000000000 |
| SE1601 | − | - | - | - | + | $stx_{1a}$ | O26:H11 | 21 | BD | ≥10 years | NA | JABKDU000000000 |
| SEH1203 | − | - | - | - | + | $stx_{2a}$ | O109:H21 | 40 | HUS | NA | NA | JABWFV000000000 |
| SEH1004 | − | - | - | - | + | $stx_{2c}$ | O26:H11 | 29 | HUS | NA | NA | JABWFP000000000 |
| SE1101 | − | - | - | - | − | $stx_{2a}$ | O104:H4 | 678 | BD | ≥10 years | Short | JABKGN000000000 |
| SE1102 | − | - | - | - | − | $stx_{2a}$ | O104:H4 | 678 | HUS | ≥10 years | NA | JABKGM000000000 |
| SE1103 | − | - | - | - | − | $stx_{2a}$ | O104:H4 | 678 | HUS | ≥10 years | Long | JABKGL000000000 |
| SE0905 | − | - | - | - | − | $stx_{2d}$ | O113:H4 | 10 | NBS | ≥10 years | NA | JABKGZ000000000 |
| SE0704 | − | - | - | - | − | $stx_{2c}$ | O8:H9 | 410 | NBS | ≥10 years | Short | JABKID000000000 |
| SEH1401 | − | - | - | - | + | $stx_{2a}$ | O59:H19 | 1136 | HUS | NA | NA | JABWFX000000000 |
| SE0403 | − | - | - | - | − | $stx_{1a}$ | O55:H12 | 101 | NBS | <10 years | Long | JABKJQ000000000 |
| SE1003 | − | - | - | - | − | $stx_{1a}$ | O115:H10 | 10 | NBS | <10 years | Short | JABKGT000000000 |
| SE1104 | − | - | - | - | − | $stx_{2a}$ | O104:H4 | 678 | NBS | ≥10 years | NA | JABKGK000000000 |
| SE0405 | − | - | - | - | − | $stx_{1c}$ + $stx_{2b}$ | O128ab:H2 | 811 | BD | ≥10 years | Long | JABKJO000000000 |
| SE0706 | − | - | - | - | − | $stx_{2e}$ | O9:H21 | 1494 | NBS | ≥10 years | NA | JABKIB000000000 |
| SE1105 | − | - | - | - | − | $stx_{2b}$ | O146:H28 | 738 | NBS | ≥10 years | NA | JABKGJ000000000 |
| SE0602 | − | - | - | - | − | $stx_{1c}$ + $stx_{2d}$ | O76:H19 | 675 | NBS | <10 years | Short | JABKIM000000000 |
| SE0406 | − | - | - | - | − | $stx_{1a}$ | O55:H12 | 101 | NBS | <10 years | Long | JABKJN000000000 |

| | | | | | | | | | | | | |
|---|---|---|---|---|---|---|---|---|---|---|---|---|
| SE0707 | – | - | - | - | – | $stx_{1a}$ | O117:H7 | 504 | NBS | ≥10 years | NA | JABKIA000000000 |
| SE1306 | – | - | - | - | – | $stx_{1a}$ | O96:H19 | 1303 | NBS | <10 years | Long | JABKEY000000000 |
| SE1307 | – | - | - | - | – | $stx_{1a}$ | O96:H19 | 1303 | NBS | <10 years | Long | JABKEX000000000 |
| SE0306 | – | - | - | - | – | $stx_{1c}$ +$stx_{2b}$ | O15:H27 | 2388 | NBS | ≥10 years | NA | JABKJY000000000 |
| SE0709 | – | - | - | - | – | $stx_{1c}$ | O167:H14 | N[a] | NBS | <10 years | NA | JABKHY000000000 |
| SE573 | – | - | - | - | – | $stx_{2g}$ | O15:H16 | 325 | NBS | ≥10 years | Short | SAMN09758573 |
| SE1206 | – | - | - | - | – | $stx_{1a}$ | O117:H7 | 504 | NBS | <10 years | Long | JABKFP000000000 |
| SE1207 | – | - | - | - | – | $stx_{1c}$ +$stx_{2b}$ | O146:H21 | N[a] | NBS | <10 years | NA | JABKFO000000000 |
| SE1211 | – | - | - | - | – | $stx_{1a}$ | O117:H7 | 504 | NBS | <10 years | Long | JABKFK000000000 |
| SE0811 | – | - | - | - | – | $stx_{2b}$ +$stx_{2d}$ | O112ab:H2 | 388 | NBS | ≥10 years | Long | JABKHH000000000 |
| SE0812 | – | - | - | - | – | $stx_{2a}$ | O35:H10 | 398 | BD | <10 years | Short | JABKHG000000000 |
| SE0409 | – | - | - | - | – | $stx_{1a}$ | O117:H7 | 504 | NBS | <10 years | NA | JABKJK000000000 |
| SE1114 | – | - | - | - | – | $stx_{2b}$ | O146:H28 | 738 | NBS | <10 years | Short | JABKGA000000000 |
| SE1605 | – | - | - | - | – | $stx_{1c}$ | Ont:H21 | 40 | NBS | <10 years | Long | JABKDQ000000000 |
| SE1311 | – | - | - | - | – | $stx_{1d}$ | O8:H19 | 3233 | NBS | <10 years | Short | JABKET000000000 |
| SE575 | – | - | - | - | – | $stx_{2e}$ | O100:H30 | 993 | NBS | ≥10 years | NA | SAMN09758575 |
| SE0815 | – | - | - | - | – | $stx_{1a}$ | O156:H7 | 504 | NBS | ≥10 years | NA | JABKHD000000000 |
| SE1709 | – | - | - | - | – | $stx_{1c}$ | Ont:H16 | 8649 | NBS | <10 years | Short | JABKDG000000000 |
| SE1412 | – | - | - | - | – | $stx_{1a}$ | O117:H7 | 504 | NBS | ≥10 years | Long | JABKEL000000000 |
| E1147–96 | – | - | - | - | – | $stx_{2d}$ | O148:H8 | 448 | HUS | NA | NA | NA |

"–": Negative; "+": Positive; NA: not available.

Age groups : <10 years of age; ≥10 years of age.

Short: duration of shedding ≤24 days; Long: duration of shedding >24 days.

a: new STs identified.

**Table S5.** 26 reference sequences of six described *ehxA* subtypes.

| Accession No. (Strain Name) | Serogroup | *ehxA* Subtype | Sources | Sequence(5'→3') |
|---|---|---|---|---|
| AB011549.2 (Sakia) | O157 | B | Homo sapiens | ATGACAGTAAATAAAATAAAGAACATTTTCAATAATGCGACATTGAC-TACAAAATCAGCATTTAATACAG CATCATCAAGCGTACGTTCCGCTGGAAAAAAACTCATATTATTAATAC-CTGATAATTATGAAGCTCAGGG CGTGGGTATTAATGAGTTGGTCAAAGCTGCTGATGAGCTTGGAA-TAGAAATACACCGTACTGAACGAGAT GATACAGCGATTGCAAACCAGTTTTTTGGTGCAGCAGAAAAAGTTGTAG-GATTAACTGAACGTGGTGTTG CAATATTCGCACCACAACTTGACAAACTTCTGCAGAAGTATCAGAAAGTT-GGGAGTAAAATAGGAGGAAC CGCTGAAAATGTAGGTAATAATCTGGGAAAAGCCGGAACAG-TTCTCTCAGCACTACAGAATTTTACGGGG ATTGCTTTATCAGGCATGGCTCTTGATGAATTGCTGAGAAAACAACGG-GAAGGAGAGGATATAAGTCAGA ATGATATTGCCAAAAGTAGTATTGAACTTATTAATCAGCTTGTAGATA-CAGTATCAAGTATAAACAGTAC CGTTGATTCATTTTCTGAGCAGCTTAACCAGCTTGGCTCATTTTTATCCAG-TAAACCTCGATTAAGTTCT GTTGGTGGGAAATTACAAAATTTACCAGACCTGGGCCCCCTGGGG-GATGGGCTGGATGTTGTCTCCGGAA TTCTTTCTGCTGTATCAGCAAGCTTTATTCTGGGAAACAGTGACGCACATA-CAGGAACAAAAGCTGCAGC GGGTATCGAACTGACAACTCAGGTTCTTGGAAATGTTGGTAAA-GCTGTTTCGCAATATATTCTGGCTCAG AGAATGGCACAGGGGTTATCGACAACAGCTG-CAAGTGCGGGTCTGATCACATCGGCTGTTATGCTGGCTA TCAGTCCTCTTTCTTTCCTGGCTGCTGCAGATAAATTTGAGCGAGCTAA-GCAGCTTGAATCATATTCTGA ACGATTTAAAAAATTGAATTATGAAGGGGATGCTTTACTCG-CAGCCTTTCATAAAGAAACCGGAGCTATA GATGCAGCCCTGACAACAATAAATACTGTCCTGAGTTCTGTATCTGCGG-GAGTTAGTGCAGCCTCCAGTG CATCCCTCATAGGGGCCCCGATAAGCATGCTGGTGAGTGCATTAACCGG-TACGATATCTGGCATTCTGGA AGCATCAAAACAGGCTATGTTTGAGCACGTTGCAGA-GAAATTCGCTGCTCGGATCAATGAATGGGAAAAG GAGCATGGCAAAAATTATTTTGAGAATGGATATGACGCAAGA-CATGCTGCGTTTTTAGAAGACTCTCTGT CTTTGCTTGCTGATTTTTCTCGTCAGCATGCAGTAGAAAGAGCAGTCGCAA-TAACCCAGCAACATTGGGA TGAGAAGATCGGTGAACTTGCAGGCATAACCCGTAATGCTGATCGCAG-TCAGAGTGGTAAGGCATATATT AATTATCTGGAAAATGGAGGGCTTTTAGAGGCTCAACCGAAGGAG-TTTACACAACAAGTGTTTGATCCTC |

| | | | | |
|---|---|---|---|---|
| | | | | AAAAAGGGACCATAGACCTTTCAACAGGTAATGTATCAAGTGTTTT-<br>GACATTTATAACACCAACATTTAC<br>CCCAGGAGAAGAAGTTAGAGAAAGAAAACAGAGTGGTAAA-<br>TATGAATATATGACATCTCTTATTGTAAAT<br>GGTAAGGATACATGGTCTGTAAAAGGCATAAAAAATCATAAAGGTG-<br>TATATGATTATTCAAAATTGATTC<br>AGTTTGTTGAAAAGAATAACAAACACTATCAGGCGAGAA-<br>TAATTTCTGAGCTCGGAGATAAAGACGATGT<br>GGTTTATTCTGGAGCAGGCTCATCAGAAGTATTTGCTGGTGAAGGTTATGA-<br>TACCGTATCTTATAATAAG<br>ACGGATGTTGGTAAACTAACAATTGATGCAACAGGAGCATCAAAAC-<br>CTGGTGAGTATATAGTTTCAAAAA<br>ATATGTATGGTGACGTGAAGGTATTGCAGGAAGTCGTTAAGGAACAG-<br>GAGGTGTCAGTAGGGAAGCGAAC<br>AGAGAAAATACAATATCGTGATTTTGAATTCAGAACCGGTG-<br>GAATTCCTTATGATGTAATAGATAATCTT<br>CATTCTGTTGAAGAGCTCATTGGCG-<br>GAAAACATGATGATGAATTCAAAGGCGGTAAGTTTAATGATATAT<br>TCCATGGCGCAGATGGGAACGATTATATCGAAGGTAATTATGG-<br>TAATGATCGACTATACGGCGATGATGG<br>GGATGATTATATATCCGGAGGACAGGGAGACGACCAGTTATTTGGTGG-<br>TAGTGGAAACGATAAATTGAGT<br>GGAGGGGATGGTAATAATTATCTGACAGGAGGAAGCGG-<br>TAATGATGAGCTTCAGGCACACGGAGCTTATA<br>ATATTCTGTCAGGTGGTACTGGTGATGATAAACTTTATGGTGGTGGTGG-<br>TATTGATCTTCTGGATGGAGG<br>GGAAGGTAATGACTATCTGAATGGTGGTTTTGGTAATGA-<br>TATTTATGTTTATGGGCAAAACTATGGTCAT<br>CATACAATTGCAGATGAAGGAGGTAAAGGAGATCGTTTGCACTTATCTGA-<br>TATTAGCTTTGATGATATCG<br>CATTTAAGAGAGTTGGAAATGATCTTATCATGAATAAA-<br>GCCATTAATGGTGTACTTTCATTTAATGAGTC<br>AAATGATGTCAATGGGATAACATTTAAAAACTGGTTTGCGAAA-<br>GATGCCTCAGGAGCAGATAATCATCTT<br>GTTGAGGTTATAACAGATAAAGATGGTCGAGAGATAAAAGTTGATAAGA-<br>TACCTCATAATAATAATGAAC<br>GGTCAGGTTATATAAAAGCCAGTAA-<br>TATAGCATCTGAAAAAAACATGGTTAATATCACCAGTGTTGCCAA<br>TGATATTAATAAGATTATTTCTTCAGTTTCAGGGTTCGAT-<br>TCAGGTGATGAACGATTAGCATCTTTATAT<br>AATTTATCCTTACATCAAAACAACACACACTCAACAACTTTAAC-<br>GACAACTGTCTGA |
| AP0109<br>54.1<br>(11368) | O26 | C | Homo sapiens | ATGACAGTAAATAAAATAAAGAACATTTTCAATAATGCGACATTGAC-<br>TACAAAATCAGCATTTAATACAG<br>CATCATCAAGCGTACGTTCCGCTGGCAAAAAACTCATATTATTAATAC-<br>CTGATAATTATGAAGCTCAGGG<br>CGTGGGTATTAATGAGTTGGTCAAAGCTGCTGATGAGCTTGGAA-<br>TAGAAATACACCGTACTGAACGAGAT<br>GATACAGCGATTGCAAACCAGTTTTTGGTGCAGCAGAAAAAGTTGTAG-<br>GATTAACTGAACGTGGTGTTG |

CAATATTCGCACCACAACTTGACAAACTTCTGCAGAAGTATCAGAAAGTT-
GGGAGTAAAATAGGAAGAAC
CGCTGAAAATGTAGGTAATAATCTGGAAAAGCCGGAACAG-
TTCTCTCAGCACTACAGAATTTTACGGGG
ATTGCTTTATCAGGCATGGCTCTTGATGAATTGCTGAGAAAACAACGG-
GAAGGAGAGGATATAAGTCAGA
ATGATATTGCCAAAAGTAGTATTGAACTTATTAATCAGCTTGTAGATA-
CAGTATCAAGTATAAACAGTAC
CGTTGATTCATTTTCTGAGCAGCTTAACCAGCTTGGCTCATTTTTATCCAG-
TAAACCTCGCTTAAGTTCT
GTTGGTGGGAAATTACAAAATTTACCAGACCTGGGCTCCCTGGGG-
GATGGGCTGGATGTTGTCTCCGGAA
TTCTTTCTGCTGTATCAGCAAGCTTTATTCTGGGAAACAGTGACGCACATA-
CAGGAACAAAAGCTGCAGC
GGGTATCGAACTGACAACTCAGGTTCTTGGAAATGTTGGTAAA-
GCTGTTTCGCAATATATTCTGGCTCAG
AGAATGGCACAGGGGTTATCGACAACAGCTG-
CAAGTGCGGGTCTGATCACATCGGCTGTTATGCTGGCTA
TCAGTCCTCTTTCTTTCCTGGCTGCTGCAGATAAATTTGAGCGAGCTAA-
GCAGCTTGAATCATATTCTGA
ACGATTTAAAAAATTGAATTATGAAGGGGATGCTTTACTCG-
CAGCCTTTCATAAAGAAACCGGAGCTATA
GATGCAGCCCTGACAACAATAAATACTGTCCTGAGTTCTGTATCTGCGG-
GAGTTAGTGCAGCCTCCAGTG
CATCCCTCATAGGGGCCCCGATAAGCATGCTGGTGAGTGCATTAACCGG-
TACGATATCTGGCATTCTGGA
AGCATCAAAACAGGCTATGTTTGAGCACGTTGCAGA-
GAAATTCGCTGCTCGGATCAATGAATGGGAAAAG
GAGCATGGCAAAAATTATTTTGAGAATGGCTATGACGCAAGA-
CATGCTGCGTTTTTAGAAGACTCTCTGT
CTTTGCTTGCTGATTTTTCTCGTCAGCATGCAGTAGAAAGAGCAGTCGCAA-
TAACCCAGCAACATTGGGA
TGAGAAGATCGGTGAACTTGCAGGCATAACCCGTAATGCTGATCGCAG-
TCAGAGTGGTAAGGCATATATT
AATTATCTGGAAAATGGAGGGCTTTTAGAGGCTCAACCGAAAGAG-
TTTACACAACAAGTGTTTGATCCTC
AAAAAGGGACCATAGACCTTTCAACAGGTAATGTATCAAGTGTTTT-
GACATTTATAACACCAACATTTAC
CCCAGGAGAAGAAGTTAGAGAAAGAAAACAGAGTGGTAAA-
TATGAATATATGACATCTCTTATTGTAAAT
GGTAAGGATACATGGTCTGTAAAAGGCATAAAAAATCATAAAGGTG-
TATATGATTATTCAAAATTGATTC
AGTTTGTTGAAAAGAATAACAAACACTATCAGGCGAGAATGAT-
TTCTGAACTCGGAGATAAAGACGATGT
GGTTTATTCTGGAGCAGGCTCATCAGAAGTATTT-
GCTGGTGAAGGTCATGATACCGTATCTTATAATAAG
ACGGATGTTGGTAAACTAACAATTGATGCAACAGGAGCATCAAAAC-
CTGGTGAGTATATAGTTTCAAAAA
ATATGTATGGTGACGTGAAGGTATTGCAGGAAGTCGTTAAGGAACAG-
GAGGTGTCAGTAGGGAAGCGAAC

| | | | | |
|---|---|---|---|---|
| AP0109 59.1 (12009) | O103 | F | Homo sapiens | AGAGAAAATACAATATCGTGATTTTGAATTCAGAACCGGTG- GAATTCCTTATGATGTAATAGATAATCTT CATTCTGTTGAAGAGCTCATTGGCG- GAAAACATGATGATGAATTCAAAGGCGGTAAGTTTAATGATATAT TCCATGGCGCAGATGGGAACGATTATATCGAAGGTAATTATGG- TAATGATCGACTATACGGCGATGATGG GGATGATTATATATCCGGAGGACAGGGAGACGACCAGTTATTTGGTGG- TAGTGGAAACGATAAATTGAGT GGAGGGGATGGTAATAATTATCTGACAGGAGGAAGCGG- TAATGATGAGCTTCAGGCACACGGAGCTTATA ATATTCTGTCAGGTGGTACTGGTGATGATAAACTTTATGGTGGTGGTGG- TATTGATCTTCTGGATGGAGG GGAAGGTAATGACTATCTGAATGGTGGTTTTGGTAATGA- TATTTATGTTTATAGGCAAAACTATGGTCAT CATACAATTGCAGATGAAGGAGGTAAAGGAGATCGTTTGCACTTATCTGA- TATTAGCTTTGATGATATCG CATTTAAGAGAGTTGGAAATGATCTTATCATGAATAAA- GCCATTAATGGTGTACTTTCATTTAATGAGTC AAATGATGTCAATGGGATAACATTTAAAAACTGGTTTGCGAAA- GATGCCTCAGGAGCAGATAATCATCTT GTTGAGGTTATAACAGATAAAGATGGTCGAGAGATAAAAGTTGATAAAA- TACCTCATAATAATAATGAAC GGTCAGGTTATATAAAAGCCAGTAA- TATAGCATCTGAAAAAAACATGGTTAATATCACCAGTGTTGCCAA TGATATTAATAAGATTATTTCTTCAGTTTCAGGGTTCGAT- TCAGGTGATGAACGATTAGCATCTTTATAT AATTTATCCTTACATCAAAACAACACACACTCAACAACTTTAAC- GACAACTGTCTGA ATGACAGTAAATAAAATAAAGAACATTTTCAATAATGCGACATTGAC- TACAAAATCAG- CATTTAATACAGCATCATCAAGCGTACGTTCCGCTGGCAAAAAACTCATAT TATTAATACCTGATAATTATGAAGCTCAGGGCGTGGGTATTAATGAGTT- GGTCAAA- GCTGCTGATGAGCTTGGAATAGAAATACACCGTACTGAACGAGATGATAC AGCGATTGCAAACCAGTTTTTTGGTGCAGCAGAAAAAGTTGTAGGAT- TAACTGAAC- GTGGTGTTGCAATATTCGCACCACAACTTGACAAACTTCTGCAGAAGTATC AGAAAGTTGGGAGTAAAATAGGAGGAACCGCTGAAAATGTAGGTAA- TAATCTGGGAAAA- GCCGGAACAGTTCTCTCAGCACTACAGAATTTTACGGGGATTGCTTTATCA GGCATGGCTCTTGATGAATTGCTGAGAAAACAACGGGAAGGAGAGGATA- TAAGTCAGAATGA- TATTGCCAAAAGTAGTATTGAACTTATTAATCAGCTTGTAGATACAGTATC AAGTATAAACAGTACCGTTGATTCATTTTCTGAGCAGCTTAACCAGCTT- GGCTCATTTTTATCCAGTAAACCTCGCTTAAGTTCTGTTGGTGGGAAATTAC AAAATTTACCAGACCTGGGCTCCCTGGGGGATGGGCTGGATGTT- GTCTCCG- GAATTCTTTCTGCTGTATCAGCAAGCTTTATTCTGGGAAACAGTGACGCAC ATACAGGAACAAAAGCTGCAGCGGGTATA- GAACTGACAACTCAGGTTCTTGGAAATGTTGG- TAAAGCTGTTTCGCAATATATTCTGGCTCAGAGAATGGCACAGGGGTTATC |

GACAACAGCTGCAAGTGCGGGTCTGATCACATCGGCTGTTATGCTGGC-
TATCAG-
TCCTCTTTCTTTCCTGGCTGCTGCAGATAAATTTGAGCGAGCTAAGCAGCTT
GAATCATATTCTGAACGATTTAAAAAATTGAATTATGAAGGG-
GATGCTTTACTCG-
CAGCCTTTCATAAAGAAACCGGAGCTATAGATGCAGCCCTGACAACAATA
AATACTGTCCTGAGTTCTGTATCTGCGGGAGTTAGTGCAGCCTCCAGTG-
CATCCCTCATAGGGGCCCCGATAAGCATGCTGGTGAGTGCATTAACCGGT
ACGATATCTGGCATTCTGGAAGCATCAAAACAGGCTATGTTTGAGCAC-
GTTGCAGA-
GAAATTCGCTGCTCGGATCAATGAATGGGAAAAGGAGCATGGCAAAAATT
ATTTTGAGAATGGCTATGACGCAAGACATGCTGCGTTTTTAGAA-
GACTCTCTGTCTTTGCTT-
GCTGATTTTTCTCGTCAGCATGCAGTAGAAAGAGCAGTCGCAATAACCCA
GCAACATTGGGATGAGAAGATCGGTGAACTTGCAGGCATAACCCG-
TAATGCTGATCGCAG-
TCAGAGTGGTAAGGCATATATTAATTATCTGGAAAATGGAGGGCTTTTAGA
GGCTCAACCGAAGGAGTTTACACAACAAGTGTTT-
GATCCTCAAAAAGGGACCATAGAC-
CTTTCAACAGGTAATGTATCAAGTGTTTTGACATTTATAACACCAACATTT
ACCCCAGGAGAAGAAGTTAGAGAAAGAAAACAGAGTGGTAAA-
TATGAATATATGACATCTCTTATTGTAAATGGTAAGGATACATGGTCTGTA
AAAGGCATAAAAAATCATAAAGGTGTATATGATTATTCAAAATTGAT-
TCAGTTTGTTGAAAA-
GAATAACAAACACTATCAGGCGAGAATAATTTCTGAGCTCGGAGATAAAG
ACGATGTGGTTTATTCTGGAGCAGGCTCATCAGAAGTATTT-
GCTGGTGAAGGTCATGATAC-
CGTATCTTATAATAAGACGGATGTTGGTAAACTAACAATTGATGCAACAG
GAGCATCAAAACCTGGTGAGTATATAGTTTCAAAAAATATGTATGGTGAC-
GTGAAGGTATTGCAGGAAGTCGTTAAGGAACAGGAGGTGTCAGTAGGGA
AGCGAACAGAGAAAATACAATATCGTGATTTTGAATTCAGAACCGGTG-
GAATTCCTTATGATGTAATAGATAATCTTCATTCTGTTGAAGAGCTCATTGG
CGGAAAACATGATGATGAATTCAAAGGCGGTAAGTTTAATGATA-
TATTCCATGGCG-
CAGATGGGAACGATTATATCGAAGGTAATTATGGTAATGATCGACTATAC
GGCGATGATGGGGATGATTATATATCCGGAGGACAGGGAGACGACCAG-
TTATTTGGTGGTAG-
TGGAAACGATAAATTGAGTGGAGGGGATGGTAATAATTATCTGACAGGAG
GAAGCGGTAATGATGAGCTTCAGGCACACGGAGCTTATAA-
TATTCTGTCAGGTGG-
TACTGGTGATGATAAACTTTATGGTGGTGGTGGTATTGATCTTCTGGATGG
AGGGGAAGGTAATGACTATCTGAATGGTGGTTTTGGTAATGA-
TATTTATGTTTATAGGCAAAACTATGGTCATCATACAATTGCAGATGAAGG
AGGTAAAGGAGATCGTTTGCACTTATCTGATATTAGCTTTGATGATATCG-
CATTTAAGAGAG-
TTGGAAATGATCTTATCATGAATAAAGCCATTAATGGTGTACTTTCATTTA
ATGAGTCAAATGATGTCAATGGGATAACATTTAAAAACTGGTTTGCGAAA-
GATGCCTCAG-
GAGCAGATAATCATCTTGTTGAGGTTATAACAGATAAAGATGGTCGAGAG
ATAAAAGTTGATAAAATACCTCATAATAATAATGAACGGTCAGGTTATA-

| | | | | |
|---|---|---|---|---|
| AP010963.1 (11128) | O111 | C | Homo sapiens | TAAAAGCCAGTAA-<br>TATAGCATCTGAAAAAAACATGGTTAATATCACCAGTGTTGCCAATGATAT<br>TAATAAGATTATTTCTTCAGTTTCAGGATTCGATTCAGGTGATGAAC-<br>GATTAGCATCTTTA-<br>TATAATTTATCCTTACATCAAAACAACACACACTCAACAACTTTAACGACA<br>ACTGTCTGA<br>ATGACAGTAAATAAAATAAAGAACATTTTCAATAATGCGACATTGAC-<br>TACAAAATCAGCATTTAATACAG<br>CATCATCAAGCGTACGTTCCGCTGGCAAAAAACTCATATTATTAATAC-<br>CTGATAATTATGAAGCTCAGGG<br>CGTGGGTATTAATGAGTTGGTCAAAGCTGCTGATGAGCTTGGAA-<br>TAGAAATACACCGTACTGAACGAGAT<br>GATACAGCGATTGCAAACCAGTTTTTGGTGCAGCAGAAAAAGTTGTAG-<br>GATTAACTGAACGTGGTGTTG<br>CAATATTCGCACCACAACTTGACAAACTTCTGCAGAAGTATCAGAAAGTT-<br>GGGAGTAAAATAGGAAGAAC<br>CGCTGAAAATGTAGGTAATAATCTGGGAAAAGCCGGAACAG-<br>TTCTCTCAGCACTACAGAATTTTACGGGG<br>ATTGCTTTATCAGGCATGGCTCTTGATGAATTGCTGAGAAAACAACGG-<br>GAAGGAGAGGATATAAGTCAGA<br>ATGATATTGCCAAAAGTAGTATTGAACTTATTAATCAGCTTGTAGATA-<br>CAGTATCAAGTATAAACAGTAC<br>CGTTGATTCATTTTCTGAGCAGCTTAACCAGCTTGGCTCATTTTTATCCAG-<br>TAAACCTCGCTTAAGTTCT<br>GTTGGTGGGAAATTACAAAATTTACCAGACCTGGGCTCCCTGGGG-<br>GATGGGCTGGATGTTGTCTCCGGAA<br>TTCTTTCTGCTGTATCAGCAAGCTTTATTCTGGGAAACAGTGACGCACATA-<br>CAGGAACAAAAGCTGCAGC<br>GGGTATCGAACTGACAACTCAGGTTCTTGGAAATGTTGGTAAA-<br>GCTGTTTCGCAATATATTCTGGCTCAG<br>AGAATGGCACAGGGGTTATCGACAACAGCTG-<br>CAAGTGCGGGTCTGATCACATCGGCTGTTATGCTGGCTA<br>TCAGTCCTCTTTCTTTCCTGGCTGCTGCAGATAAATTTGAGCGAGCTAA-<br>GCAGCTTGAATCATATTCTGA<br>ACGATTTAAAAAATTGAATTATGAAGGGGATGCTTTACTCG-<br>CAGCCTTTCATAAAGAAACCGGAGCTATA<br>GATGCAGCCCTGACAACAATAAATACTGTCCTGAGTTCTGTATCTGCGG-<br>GAGTTAGTGCAGCCTCCAGTG<br>CATCCCTCATAGGGGCCCCGATAAGCATGCTGGTGAGTGCATTAACCGG-<br>TACGATATCTGGCATTCTGGA<br>AGCATCAAAACAGGCTATGTTTGAGCACGTTGCAGA-<br>GAAATTCGCTGCTCGGATCAATGAATGGGAAAAG<br>GAGCATGGCAAAAATTATTTTGAGAATGGCTATGACGCAAGA-<br>CATGCTGCGTTTTTAGAAGACTCTCTGT<br>CTTTGCTTGCTGATTTTTCTCGTCAGCATGCAGTAGAAAGAGCAGTCGCAA-<br>TAACCCAGCAACATTGGGA<br>TGAGAAGATCGGTGAACTTGCAGGCATAACCCGTAATGCTGATCGCAG-<br>TCAGAGTGGTAAGGCATATATT<br>AATTATCTGGAAAATGGAGGGCTTTTAGAGGCTCAACCGAAGGAG-<br>TTTACACAACAAGTGTTTGATCCTC |

| | | | | |
|---|---|---|---|---|
| | | | | AAAAAGGGACCATAGACCTTTCAACAGGTAATGTATCAAGTGTTTT-<br>GACATTTATAACACCAACATTTAC<br>CCCAGGAGAAGAAGTTAGAGAAAGAAAACAGAGTGGTAAA-<br>TATGAATATATGACATTTCTTATTGTAAAT<br>GGTAAGGATACATGGTCTGTAAAAGGCATAAAAAATCATAAAGGTG-<br>TATATGATTATTCAAAATTGATTC<br>AGTTTGTTGAAAAGGATAACAAACACTATCAGGCGAGAATGAT-<br>TTCTGAACTCGGAGATAAAGACGATGT<br>GGTTTATTCTGGAGCAGGCTCATCAGAAGTATTT-<br>GCTGGTGAAGGTCATGATACCGTATCTTATAATAAG<br>ACGGATGTTGGTAAACTAACAATTGATGCAACAGGAGCATCAAAAC-<br>CTGGTGAGTATATAGTTTCAAAAA<br>ATATGTATGGTGACGTGAAGGTATTGCAGGAAGTCGTTAAGGAACAG-<br>GAGGTGTCAGTAGGGAAGCGAAC<br>AGAGAAAATACAATATCGTGATTTTGAATTCAGAACCGGTG-<br>GAATTCCTTATGATGTAATAGATAATCTT<br>CATTCTGTTGAAGAGCTCATTGGCG-<br>GAAAACATGATGATGAATTCAAAGGCGGTAAGTTTAATGATATAT<br>TCCATGGCGCAGATGGGAACGATTATATCGAAGGTAATTATGG-<br>TAATGATCGACTATACGGCGATGATGG<br>GGATGATTATATATCCGGAGGACAGGGAGACGACCAGTTATTTGGTGG-<br>TAGTGGAAACGATAAATTGAGT<br>GGAGGGGATGGTAATAATTATCTGACAGGAGGAAGCGG-<br>TAATGATGAGCTTCAGGCACACGGAGCTTATA<br>ATATTCTGTCAGGTGGTACTGGTGATGATAAACTTTATGGTGGTGGTGG-<br>TATTGATCTTCTGGATGGAGG<br>GGAAGGTAATGACTATCTGAATGGTGGTTTTGGTAATGA-<br>TATTTATGTTTATAGGCAAAACTATGGTCAT<br>CATACAATTGCAGATGAAGGAGGTAAAGGAGATCGTTTGCACTTATCTGA-<br>TATTAGCTTTGATGATATCG<br>CATTTAAGAGAGTTGGAAATGATCTTATCATGAATAAA-<br>GCCATTAATGGTGTACTTTCATTTAATGAGTC<br>AAATGATGTCAATGGGATAACATTTAAAAACTGGTTTGCGAAA-<br>GATGCCTCAGGAGCAGATAATCATCTT<br>GTTGAGGTTATAACAGATAAAGATGGTCGAGAGATAAAAGTTGATAAAA-<br>TACCTCATAATAATAATGAAC<br>GGTCAGGTTATATAAAAGCCAGTAATATAGCATCTGAAAAAA-<br>GCATGGTTAATATCACCAGTGTTGCCAA<br>TGATATTAATAAGATTATTTCTTCAGTTTCAGGGTTCGAT-<br>TCAGGTGATGAACGATTAGCATCTTTATAT<br>AATTTATCCTTACATCAAAACAACACACACTCAACAACTTTAAC-<br>GACAACTGTCTGA |
| AY258<br>503.2<br>(EH41) | O113 | A | Homo<br>sapiens | ATGACAGTAAATAAAATAAAGAACATTTTCAACAATGCGACATCGAC-<br>TACAAAATCAG-<br>CATTTAATACAGCATCATCAAGCGTACGTTCCGCTGGAAAAAAACTCATA<br>TTATTAATACCTGATAATTATGAAGCTCAGGGCGTGGGTATTAATGAGTT-<br>GGTCAAA-<br>GCTGCTGATGAGCTTGGAATAGAAATACACCGTACTGAACGAGATGATAC<br>AGCGATTGCAAACCAGTTTTTTGGTACAGCAGAAAAAGTTGTAGGAT-<br>TAACTGAAC-<br>GTGGTGTTGCAATATTCGCACCACAACTTGACAAACTTCTGCAGAAGTATC |

AGAAAGTTGGGAGTAAAATAGGCGGAACTGCTGAAAATGTAGGTAA-
TAATCTGGGAAAA-
GCCGGAACAGTTCTCTCAGCACTACAGAATTTTACGGGGATTGCTTTATCA
GGCATGGCTCTTGATGAATTGCTGAGAAAACAACGGGAAGGAGAGGATA-
TAAGTCAGAATGA-
TATTGCCAAAAGTAGTATTGAACTTATTAATCAGCTTGTAGATACAGTATC
AAGTATAAACAGTACCGTTGATTCATTTTCTGAGCAGCTTAACCAGCTT-
GGCTCATTTTTATCCAGTAAACCTCGCTTAAGTTCTGTTGGTGGAAAATTAC
AAAATTTACCAGACCTGGGCCCCCTGGGGGATGGGCTGGATGTT-
GTCTCCG-
GAATTCTTTCTGCTGTATCAGCAAGTTTTATTCTGGGAAACAGTGACGCAC
ATACAGGAACAAAAGCTGCAGCGGG-
TATCGAACTGACAACTCAGGTTCTTGGAAATGTTGG-
TAAAGCTGTTTCGCAATATATTCTGGCTCAGAGAATGGCACAGGGATTATC
GACAACAGCTGCAAGTGCGGGTCTGATCACATCGGCTGTTATGCTGGC-
TATCAG-
TCCTCTTTCTTTCCTGGCTATTGCAGATAAATTTGAGCGAGCTAAGCAGCTT
GAATCATATTCTGAACGATTTAAAAAATTCAATTATGAAGGA-
GATGCTTTACTCG-
CAGCCTTTCATAAAGAAAGCGGAGCTATAGATGCAGCCCTGACAACAATA
AATACTGTCCTGAGTTCTGTATCTGCGGGAGTTAGTGCAGCCTCCAGTG-
CATCCCTCATAGGGGCCCCGATAAGCATGCTGGTGAGTGCATTAACCGGT
ACGATATCTGGCATTCTGGAAGCATCAAAACAGGCTATGTTTGAGCAC-
GTTGCAGA-
TAAATTCGCTGCTCGGATCAATGAATGGGAAAAGGAGCATGGCAAAAATT
ATTTTGAGAATGGCTATGACGCAAGACATGCTGCGTTTTTAGAA-
GACTCTCTGTCTTTGCTT-
GCTGATTTTTCTCGTCAGCATGCAGTAGAAAGAGCTGTCGCAATAACCCAG
CAACATTGGGATGAGAAGATCGGTGAACTTGCAGGTATAACCCG-
TAATGCTGATCGCAG-
TCAGAGTGGTAAGGCATATATTAATTATCTGGAGAATGGAGGGCTTTTAGA
GGCTCAACCGAAGGAGTTTACACAACAAGTTTTT-
GATCCTCAAAAAGGGACTATAGAC-
CTTTCAACAGGTAATGTATCAAGTGTTTTGACATTTGTAACACCAACATTT
ACCCCAGGAGAAGAAGTCAGAGAAAGAAAACAGAGTGGTAAA-
TATGAATATATGACATCTCTTATTGTAAATGGTAAGGATACATGGTCTGTA
AAAGGCATAAAAAAATCATAAAGGTGTATATGATTATTCAAATTTGAT-
TCAGTTTGTT-
GAAAAGGATAACAAACACTATCAGGCGAGAATAATTTCTGAGCTCGGAG
ATAAAGACGATATAGTTTATTCTGGGGCAGGCTCATCAGAAGTATTT-
GCTGGTGAAGGTCATGATACCGTATCTTATAATAAGACGGATGTTGGTAAA
CTAACAATTGATGCAACAGGAGCATCAAAAC-
CTGGTGAATATATAGTTTCAAAAAA-
TATGTATGGTGACGTGAAGGTATTGCAGGAAGTCGTTAAGGAACAGGAGG
TGTCAGTAGGGAAGAGAACAGAGAAAATACAATATCGTGATTTT-
GAATTCAGAACCGGTG-
GAATTCCTTATGATGTGATAGATAATCTTCATTCTGTTGAAGAACTCATTGG
CGGAAAACATGATGATGAATTCAAAGGCGGTAAGTTTAATGATA-
TATTCCATGGTG-
CAGATGGGAACGATTATATCGAAGGTAATTATGGTAATGATCGACTATAC

| | | | | |
|---|---|---|---|---|
| CP0060 28.1 (RM13 514) | O145 | C | Homo sapiens | GGCGATGATGGGGATGATTATATATCCGGTGGACAGGGAGACGACCAG-<br>TTATTTGGTGGTAG-<br>TGGAAACGATAAATTGAGTGGAGGGGATGGTAATAATTATCTGACAGGAG<br>GACGCGGTAATGATGAGCTTCAGGCACACGGAGCTTATAA-<br>TATTCTGTCAGGTGG-<br>TACTGGTGATGATAAACTTTATGGTGGTGGTGGTATTGACCTTCTGGATGG<br>AGGGGAAGGTAATGACTATCTGAATGGTGGTTTTGGTAATGA-<br>TATTTATGTTTATAGGCAAAACTATGGTCATCATACAATTGCAGATGAAGG<br>AGGTAAAGGAGATCGTCTGCACTTATCTGATATTAGCTTTGATGATATCG-<br>CATTTAA-<br>GAAAGTTGGAAATGATCTTATCATGAATAAAGCCATTAATGGTGCACTTTC<br>ATTTAATGAGTCAAATGATGTCAATGGGATAACATTTAAAAACTGGTTT-<br>GCGAAA-<br>GATGCCTCAGGTGAAGATAATCATCTTGTTGAGGTTATAACAGATAAAGA<br>TGGTCGTGAGATAAAAGCTGATAAGATATCTCATAATAATAATGAACAG-<br>TCAGGTTATA-<br>TAAAAGCCAGTAATATAGCATCTGAAAAAAACATGGTTAATATCACTAGT<br>GTTGCCAATGATATTAACAAGATTATTTCTTCAGTTTCAGGGTTCGAT-<br>TCAGGTGATGAAC-<br>GATTAGCATCTTTATATAATTTATCCTTACATCAAAATAACACACACTCAA<br>CAACTTTAACGACAACTGTCTGA<br>ATGACAGTAAATAAAATAAAGAACATTTTCAATAATGCGACATTGAC-<br>TACAAAATCAG-<br>CATTTAATACAGCATCATCAAGCGTACGTTCCGCTGGCAAAAAACTCATAT<br>TATTAATACCTGATAATTATGAAGCTCAGGGCGTGGGTATTAATGAGTT-<br>GGTCAAA-<br>GCTGCTGATGAGCTTGGAATAGAAATACACCGTACTGAACGAGATGATAC<br>AGCGATTGCAAACCAGTTTTTTGGTGCAGCAGAAAAAGTTGTAGGAT-<br>TAACTGAAC-<br>GTGGTGTTGCAATATTCGCACCACAACTTGACAAACTTCTGCAGAAGTATC<br>AGAAAGTTGGGAGTAAAATAGGAAGAACCGCTGAAAATGTAGGTAA-<br>TAATCTGGGAAAA-<br>GCCGGAACAGTTCTCTCAGCACTACAGAATTTTACGGGGATTGCTTTATCA<br>GGCATGGCTCTTGATGAATTGCTGAGAAAACAACGGGAAGGAGAGGATA-<br>TAAGTCAGAATGA-<br>TATTGCCAAAAGTAGTATTGAACTTATTAATCAGCTTGTAGATACAGTATC<br>AAGTATAAACAGTACCGTTGATTCATTTTCTGAGCAGCTTAACCAGCTT-<br>GGCTCATTTTTATCCAGTAAACCTCGCTTAAGTTCTGTTGGTGGGAAATTAC<br>AAAATTTACCAGACCTGGGCTCCCTGGGGGATGGGCTGGATGTT-<br>GTCTCCG-<br>GAATTCTTTCTGCTGTATCAGCAAGCTTTATTCTGGGAAACAGTGACGCAC<br>ATACAGGAACAAAAGCTGCAGCGGG-<br>TATCGAACTGACAACTCAGGTTCTTGGAAATGTTGG-<br>TAAAGCTGTTTCGCAATATATTCTGGCTCAGAGAATGGCACAGGGGTTATC<br>GACAACAGCTGCAAGTGCGGGTCTGATCACATCGGCTGTTATGCTGGC-<br>TATCAG-<br>TCCTCTTTCTTTCCTGGCTGCTGCAGATAAATTTGAGCGAGCTAAGCAGCTT<br>GAATCATATTCTGAACGATTTAAAAAATTGAATTATGAAGGG-<br>GATGCTTTACTCG-<br>CAGCCTTTCATAAAGAAACCGGAGCTATAGATGCAGCCCTGACAACAATA |

AATACTGTCCTGAGTTCTGTATCTGCGGGAGTTAGTGCAGCCTCCAGTG-
CATCCCTCATAGGGGCCCCGATAAGCATGCTGGTGAGTGCATTAACCGGT
ACGATATCTGGCATTCTGGAAGCATCAAAACAGGCTATGTTTGAGCAC-
GTTGCAGA-
GAAATTCGCTGCTCGGATCAATGAATGGGAAAAGGAGCATGGCAAAAATT
ATTTTGAGAATGGCTATGACGCAAGACATGCTGCGTTTTTAGAA-
GACTCTCTGTCTTTGCTT-
GCTGATTTTTCTCGTCAGCATGCAGTAGAAAGAGCAGTCGCAATAACCCA
GCAACATTGGGATGAGAAGATCGGTGAACTTGCAGGCATAACCCG-
TAATGCTGATCGCAG-
TCAGAGTGGTAAGGCATATATTAATTATCTGGAAAATGGAGGGCTTTTAGA
GGCTCAACCGAAAGAGTTTACACAACAAGTGTTT-
GATCCTCAAAAAGGGACCATAGAC-
CTTTCAACAGGTAATGTATCAAGTGTTTTGACATTTATAACACCAACATTT
ACCCCAGGAGAAGAAGTTAGAGAAAGAAAACAGAGTGGTAAA-
TATGAATATATGACATCTCTTATTGTAAATGGTAAGGATACATGGTCTGTA
AAAGGCATAAAAAATCATAAAGGTGTATATGATTATTCAAAATTGAT-
TCAGTTTGTTGAAAA-
GAATAACAAACACTATCAGGCGAGAATGATTTCTGAACTCGGAGATAAAG
ACGATGTGGTTTATTCTGGAGCAGGCTCATCAGAAGTATTT-
GCTGGTGAAGGTCATGATAC-
CGTATCTTATAATAAGACGGATGTTGGTAAACTAACAATTGATGCAACAG
GAGCATCAAAACCTGGTGAGTATATAGTTTCAAAAAATATGTATGGTGAC-
GTGAAGGTATTGCAGGAAGTCGTTAAGGAACAGGAGGTGTCAGTAGGGA
AGCGAACAGAGAAAATACAATATCGTGATTTTGAATTCAGAACCGGTG-
GAATTCCTTATGATGTAATAGATAATCTTCATTCTGTTGAAGAGCTCATTGG
CGGAAAACATGATGATGAATTCAAAGGCGGTAAGTTTAATGATA-
TATTCCATGGCG-
CAGATGGGAACGATTATATCGAAGGTAATTATGGTAATGATCGACTATAC
GGCGATGATGGGGATGATTATATATCCGGAGGACAGGGAGACGACCAG-
TTATTTGGTGGTAG-
TGGAAACGATAAATTGAGTGGAGGGGATGGTAATAATTATCTGACAGGAG
GAAGCGGTAATGATGAGCTTCAGGCACACGGAGCTTATAA-
TATTCTGTCAGGTGG-
TACTGGTGATGATAAACTTTATGGTGGTGGTGGTATTGATCTTCTGGATGG
AGGGGAAGGTAATGACTATCTGAATGGTGGTTTTGGTAATGA-
TATTTATGTTTATAGGCAAAACTATGGTCATCATACAATTGCAGATGAAGG
AGGTAAAGGAGATCGTTTGCACTTATCTGATATTAGCTTTGATGATATCG-
CATTTAAGAGAG-
TTGGAAATGATCTTATCATGAATAAAGCCATTAATGGTGTACTTTCATTTA
ATGAGTCAAATGATGTCAATGGGATAACATTTAAAAACTGGTTTGCGAAA-
GATGCCTCAG-
GAGCAGATAATCATCTTGTTGAGGTTATAACAGATAAAGATGGTCGAGAG
ATAAAAGTTGATAAAATACCTCATAATAATAATGAACGGTCAGGTTATA-
TAAAAGCCAGTAA-
TATAGCATCTGAAAAAAACATGGTTAATATCACCAGTGTTGCCAATGATAT
TAATAAGATTATTTCTTCAGTTTCAGGGTTCGATTCAGGTGATGAAC-
GATTAGCATCTTTA-
TATAATTTATCCTTACATCAAAACAACACACACTCAACAACTTTAACGACA
ACTGTCTGA

| | | | | |
|---|---|---|---|---|
| CP009107.1(94-3024) | O104 | A | Homo sapiens | ATGACAGTAAATAAAATAAAGAACATTTTCAACAATGCGACATCGAC-<br>TACAAAATCAG-<br>CATTTAATACAGCATCATCAAGCGTACGTTCCGCTGGAAAAAAACTCATA<br>TTATTAATACCTGATAATTATGAAGCTCAGGGCGTGGGTATTAATGAGTT-<br>GGTCAAA-<br>GCTGCTGATGAGCTTGGAATAGAAATACACCGTACTGAACGAGATGATAC<br>AGCGATTGCAAACCAGTTTTTTGGTACAGCAGAAAAAGTTGTAGGAT-<br>TAACTGAAC-<br>GTGGTGTTGCAATATTCGCACCACAACTTGACAAACTTCTGCAGAAGTATC<br>AGAAAGTTGGGAGTAAAATAGGAGGAACCGCTGAAAATGTAGGTAA-<br>TAATCTGGGAAAA-<br>GCCGGAACAGTTCTCTCAGCACTACAGAATTTTACGGGGATTGCTTTATCA<br>GGCATGGCTCTTGATGAATTGCTGAGAAAACAACGGGAAGGAGAGGATA-<br>TAAGTCAGAATGA-<br>TATTGCCAAAAGTAGTATTGAACTTATTAATCAGCTTGTAGATACAGTATC<br>AAGTATAAACAGTACCGTTGATTCATTTTCTGAGCAGCTTAACCAGCTT-<br>GGTTCATTTTTATCCAGTAAACCTCGCTTAAGTTCTGTTGGTGGAAAATTAC<br>AAAATTTACCAGACCTGGGCCCCCTGGGGGATGGGCTGGATGTT-<br>GTCTCCG-<br>GAATTCTTTCTGCTGTATCAGCAAGTTTTATTCTGGGAAACAGTGACGCAC<br>ATACAGGAACAAAAGCTGCAGCGGG-<br>TATCGAACTGACAACTCAGGTTCTTGGAAATGTTGG-<br>TAAAGCTGTTTCGCAATATATTCTGGCTCAGAGAATGGCACAGGGATTATC<br>GACAACAGCTGCAAGTGCGGGTCTGATCACATCGGCTGTTATGCTGGC-<br>TATCAG-<br>TCCTCTTTCTTTCCTGGCTATTGCAGATAAATTTGAGCGAGCTAAGCAGCTT<br>GAATCATATTCTGAACGATTTAAAAAATTCAATTATGAAGGG-<br>GATGCTTTACTCG-<br>CAGCCTTTCATAAAGAAAGCGGAGCTATAGATGCAGCCCTGACAACAATA<br>AATACTGTCCTGAGTTCTGTATCTGCGGGAGTTAGTGCAGCCTCCAGTG-<br>CATCCCTCATAGGGGCCCCGATAAGCATGCTGGTGAGTGCATTAACCGGT<br>ACGATATCTGGCATTCTGGAAGCATCAAAACAGGCTATGTTTGAGCAC-<br>GTTGCAGA-<br>TAAATTCGCTGCTCGGATCAATGAATGGGAAAAGGAGCATGGCAAAAATT<br>ATTTTGAGAATGGCTATGACGCAAGACATGCTGCGTTTTTAGAA-<br>GACTCTCTGTCTTTGCTT-<br>GCTGATTTTTCTCGTCAGCATGCAGTAGAAAGAGCTGTCGCAATAACCCAG<br>CAACATTGGGATGAGAAGATCGGTGAACTTGCAGGTATAACCCG-<br>TAATGCTGATCGCAG-<br>TCAGAGTGGTAAGGCATATATTAATTATCTGGAGAATGGAGGGCTTTTAGA<br>GGCTCAACCGAAGGAGTTTACACAACAAGTTTTT-<br>GATCCTCAAAAAGGGACTATAGAC-<br>CTTTCAACAGGTAATGTATCAAGTGTTTTGACATTTGTAACACCAACATTT<br>ACCCCAGGAGAAGAAGTCAGAGAAAGAAAACAGAGTGGTAAA-<br>TATGAATATATGACATCTCTTATTGTAAATGGTAAGGATACATGGTCTGTA<br>AAAGGCATAAAAAATCATAAAGGTGTATATGATTATTCAAATTTGAT-<br>TCAGTTTGTT-<br>GAAAAGGATAACAAACACTATCAGGCGAGAATAATTTCTGAGCTCGGAG<br>ATAAAGACGATATAGTTTATTCTGGGGCAGGCTCATCTGAAGTATTT-<br>GCTGGTGAAGGTCATGATACCGTATATTATAATAAGACGGATGTTGGTAA |

| | | | | |
|---|---|---|---|---|
| CP012493.1 (CFSAN004176) | O145 | E | Homo sapiens | ACTAACAATTGATGCAACAGGAGCATCAAAAC-<br>CTGGTGAATATATAGTTTCAAAAAA-<br>TATGTATGGTGACGTGAAGGTATTGCAGGAAGTCGTTAAGGAACAGGAGG<br>TATCAGTAGGGAAGAGAACAGAGAAAATACAATATCGTGATTTT-<br>GAATTCAGAACCGGTG-<br>GAATTCCTTATGATGTGATAGATAATCTTCATTCTGTTGAAGAACTCATTGG<br>CGGAAAACATGATGATGAATTCAAAGGCGGTAAGTTTAATGATA-<br>TATTCCATGGTG-<br>CAGATGGGAACGATTATATCGAAGGTAATTATGGTAATGATCGACTATAC<br>GGCGATGATGGGGATGATTATATATCCGGTGGACAGGGAGACGACCAG-<br>TTATTTGGTGGTAG-<br>TGGAAACGATAAATTGAGTGGAGGGGATGGTAATAATTATCTGACAGGAG<br>GAAGCGGTAATGATGAGCTTCAGGCACACGGAGCTTATAA-<br>TATTCTGTCAGGTGG-<br>TACTGGTGATGATAAACTTTATGGTGGTGGTGGTATTGACCTTCTGGATGG<br>AGGGGAAGGTAATGACTATCTGAATGGTGGTTTTGGTAATGA-<br>TATTTATGTTTATAGGCAAAACTATGGTCATCATACAATTGCAGATGAAGG<br>AGGTAAAGGAGATCGTCTGCACTTATCTGATATTAGCTTTGATGATATCG-<br>CATTTAA-<br>GAAAGTTGGAAATGATCTTATCATGAATAAAGCCATTAATGGTGTACTTTC<br>ATTTAATGAGTCAAATGATGTCAATGGGATAACATTTAAAAACTGGTTT-<br>GCGAAA-<br>GATGCCTCAGGTGAAGATAATCATCTTGTTGAGGTTATAACAGATAAAGA<br>TGGTCGTGAGATAAAAGCTGATAAGATATCTCATAATAATAATGAACAG-<br>TCAGGTTATA-<br>TAAAAGCCAGTAATATAGCATCTGAAAAAAACATGGTTAATATCACTAGT<br>GTTGCCAATGATATTAACAAGATTATTTCTTCAGTTTCAGGGTTCGAT-<br>TCAGGTGATGAAC-<br>GATTAGCATCTTTATATAATTTATCCTTACATCAAAATAACACACACTCAA<br>CAACTTTAACGACAACTGTCTGA<br>ATGACAGCAAATAAAATAAAGAGCATTTTCAATAATGCTGCATTGAC-<br>TACAAAATCAG-<br>CATTTAATACAGCATCATCAAGCGTACGTTCCGCTGGAAAAAAACTCATA<br>TTATTAATACCTGATAATTATGAAGCTCAGGGCGTGGGTATTAATGAGTT-<br>GGTCAAA-<br>GCTGCTGATGAGCTTGGAATAGAAATACACCGTACTGAACGAGATGATAC<br>AGCGATTGCAAACCAGTTTTTGGTACAGCAGAAAAAGTTGTAGGAT-<br>TAACTGAAC-<br>GTGGTGTTGCAATATTCGCACCACAACTTGACAAACTTCTGCAGAAGTATC<br>AGAAAGTTGGGAGTAAAATAGGAGGAACCGCTGAAAATGTAGGTAA-<br>TAATCTGGGAAAA-<br>GCCGGAACAGTTCTCTCAGCACTACAGAATTTTACGGGGATTGCTTTATCA<br>GGCATGGCTCTTGATGAATTGCTGAGAAAACAACGGGAAGGAGAGGATA-<br>TAAGTCAGAATGA-<br>TATTGCCAAAAGTAGTATTGAACTTATTAATCAACTTGTAGATACAGTATC<br>AAGTATAAACAGTACCGTTGATTCATTTTCTGAGCAGCTTAACCAGCTT-<br>GGCTCATTTTTATCCAGTAAACCTCGCTTAAGTTCTGTTGGTGGAAAATTAC<br>AAAATTTACCGGACCTGGGCCCCTTGGGGGATGGGCTGGATGTT-<br>GTCTCCG-<br>GAATTCTTTCTGCTGTATCAGCAAGCTTTATTCTGGGAAACAGTGACGCAC |

ATACAGGAACAAAAGCTGCAGCGGG-
TATCGAACTGACAACTCAGGTTCTTGGAAATGTTGG-
TAAAGCTGTTTCGCAATATATTCTGGCTCAGAGAATGGCACAGGGGTTATC
GACAACAGCTGCAAGTGCGGGTCTGATCACATCGGCTGTTATGCTGGC-
TATCAG-
TCCTCTTTCTTTCCTGGCTATTGCAGATAAATTTGAGCGAGCTAAGCAGCTT
GAATCATATTCTGAACGATTTAAAAAATTGAATTATGAAGGG-
GATGCTTTACTCG-
CAGCCTTTCATAAAGAAACCGGAGCTATAGATGCAGCCCTGACAACAATA
AATACTGTCCTGAGTTCTGTATCTGCGGGAGTTAGTGCAGCCTCCAGTG-
CATCCCTCATAGGGGCCCCGATAAGCATGCTGGTGAGTGCATTAACCGGT
ACGATATCTGGCATTCTGGAAGCATCAAAACAGGCTATGTTTGAGCAC-
GTTGCAGA-
TAAATTCGCTGCTCGGATCAATGAATGGGAAAAGGAGCATGGCAAAAATT
ATTTTGAGAATGGCTATGACGCAAGACATGCTGCGTTTTTAGAAGAT-
TCTCTGTCTTTGCTT-
GCTGATTTTTCTCGTCAGCATGCAGTAGAAAGAGCTGTCGCAATAACCCAG
CAACATTGGGATGAGAAGATCGGTGAACTTGCAGGTATAACCCG-
TAATGCTGATCGCAG-
TCAGAGTGGTAAGGCATATATTAATTATCTGGAGAATGGAGGGCTTTTAGC
GGCTCAACCGAAGGAGTTTACACAACAAGTTTTT-
GATCCTCAAAAAGGGACTATAGAC-
CTTTCAACAGGTAATGTATCAAGTGTTTTGACATTTGTAACACCAACATTT
ACCCCAGGAGAAGAAGTCAGAGAAAGAAAACAGAGTGGTAAA-
TATGAATATATGACATCTCTTATTGTAAATGGTAAGGATACATGGTCTGTA
AAAGGCATAAAAAATCATAAAGGTGTATATGATTATTCAAATTTGAT-
TCAGTTTGTT-
GAAAAGGATAACAAACACTATCAGGCGAGAATAATTTCTGAGCTCGGAG
ATAAAGACGATATAGTTTATTCTGGGGCTGGCTCATCAGAAGTATTT-
GCTGGTGAAGGCCATGATACCGTATCTTATAATAAGACGGATGTTGGTAA
ACTAACAATTAATGCAACAGGAGCATCAAAAC-
CTGGTGAATATATAGTTTCAAAAAA-
TATGTATGGTGACGTGAAGGTATTGCAGGAAGTCGTTAAGGAACAGGAGG
TGTCAGTAGGGAAGCGAACAGAGAAAATACAATATCGTGATTTT-
GAATTCAGAACCGGTG-
GAATTCCTTATGATGTGATAGATAATCTTCATTCTGTTGAAGAACTCATTGG
CGGAAAACATGATGATGAATTCAAAGGCGGTAAGTTTAATGATA-
TATTCCATGGCG-
CAGATGGGAACGATTATATCGAAGGTAATTATGGTAATGATCGACTATAC
GGCGATGATGGGGATGATTATATATCCGGAGGACAGGGAGACGACCAG-
TTATTTGGTGGTAG-
TGGAAACGATAAATTGAGTGGAGGGGATGGTAATAATTATCTGACAGGAG
GAAGCGGTAATGATGAGCTTCAGGCACACGGGGCTTATAA-
TATTCTGTCAGGTGG-
TACTGGTGATGATAAACTTTATGGTGGTGGTGGTATTGACCTTCTGGATGG
AGGGGAAGGTAATGACTATCTGAATGGTGGTTTTGGTAATGA-
TATTTATGTTTATAGGCAAAACTATGGTCATCATACAATTGCAGATGAAGG
AGGTAAAGGAGATCGTCTGCACTTATCTGATATTAGCTTTGATGATATCG-
CATTTAAGAGAG-
TTGGAAATGATCTTATCATGAATAAAGCCATTAATGGTGTACTTTCATTTA

| | | | | |
|---|---|---|---|---|
| CP012498.1 (06-00048) | O36 | D | Alfalfa sprouts | ATGAGTCAAATGATGTCAATGGGATAACATTTAAAAACTGGTTTGAGAAA-GATGCCTCAG-GAGCAGATAATCATCTTGTTGAGGTTATAACAGATAAAGATGGTCGTGAG ATAAAAGCTGATAAGATACCTCATAATAATAATGAACGGTCAGGTTATA-TAAAAGCCAGTAA-TATAGCATCTGAAAAAAACATGGTTAATATCACCAGTGTTGCCGATGATAT TAACAAGATTATTTCTTCAGTTTCAGGGTTCGATTCAGGTGATGAAC-GATTAGCATCTTTA-TATAATTTATCTTTACATCAAAACAACACACACTCAACAACTTTAACGACA ACTGTCTGA ATGACAGTAAATAAAATAAAGAACATTTTCAGCAATGCGACATCGAC-TACAAAATCAGCATTTAATACAG CATCATCAAACGTACGTACCGCTGGAAAAAAACTCATATTATTAATAC-CTGATGATTATGAAGCTCAGGG CGTGGGTATTAATGAGTTGGTCAAAGCTGCTGATGAGCTTGGAA-TAGAAATACACCGTACTGAACGAGAT AATACAACGATTGCAAACCAGTTTTTTGGTACAGCAGAAAAAGTTTTAG-GATTAACTGAACGTGGTGTTG CAATATTCGCACCACAACTTGATAAACTTCTGCAGAAGTATCAGAACGTT-GGGAGTAAAATAGGCGGAAC CGCTGAGAATGTAGGTAATAATCTGGGAAAAGCCGGAACAGTTCTTTCAG-CACTACAGAATTTTACGGGG ATTGCCTTATCAGGCATGGCTCTTGATGAATTGCTGAGAAAACAACGG-GAAGGAGAGGATATAAGTCAGA ATGATATTGCCAAAAGTAGTATTGAACTTATTAATCACCTTGTAGATA-CAGTATCAAGTATAAACAGTAC TGTTGATTCATTTTCTGAGCAGCTTAACCAGCTTGGCTCATTTTTATCCAG-TAAACCTCGCTTAAGTTCT GTTGGTGGAAAATTACAAAATTTACCTGACCTGGGCCCCTTGGGG-GATGGGCTGGATGTTATCTCTGGAA TTCTTTCAGCTGTATCAGCAATCTTTATTCTGGGAAACAGTGACGCACATA-CAGGAACAAAAGCAGCAGC AGGTATCGAACTGACAACTCAGGTTCTGGGAAATGTTGGTAAA-GCTGTTTCACAATATATTCTGGCTCAG AGAATGGCACAGGGCTTATCGACAACAGCTG-CAAGTGCGGGTCTGATCACATCGGCTGTTATGCTGGCTA TCAGTCCTCTTTCTTTCCTGGCTATTGCAGATAAATTTGAGCGAGCTAA-GCAGCTTGAAGCATATTCTGA ACGATTTAAAAAAATTGAATTATGAAGGGGATGCTTTACTT-GCAGCCTTTCATAAAGAAACCGGAGCTATA GATGCAGCCCTGACAACAATAAATACTGTTCTGAGTTCTGTATCTGCGG-GAGTTAGTGCAGCCTCCAGTG CATCCCTCATAGGGGCCCCGATAAGCATGCTGGTGAGTGCATTAACCGG-TACGATATCTGGCATTCTGGA AGCATCAAAACAGGCTATGTTTGAGCACGTTGCAGA-TAAATTCGCTGCTCGGATCAATGAATGGGAAAAG GAGCATGGCAAAAATTATTTTGAGAATGGCTATGATGCAAGACATGCTG-CATTTTTAGAAGACTCTCTGT CTTTGCTTGCAGATTTTTCTCGTCAGCATGCAGTAGAAAGAGCTGTCGCAA-TAACTCAGCAACATTGGGA |

| | | | | |
|---|---|---|---|---|
| | | | | TGAGAAGATCGGTGAACTTGCAGGTATAACCCGTAATGCTGATCGTAG-<br>TCAGAGTGGTAAGGCATATATT<br>AATTATCTGGAGAATGGAGGGCTTTTAGAGGATCAACCGAAGGAG-<br>TTTACACAACAAGTTTTTGATCCTC<br>AAAAAGGGACCATAGACCTTTCAACAGGTAATGTATCAAGTGTTTT-<br>GACATTTGTAACACCAACATTTAC<br>CCCAGGAGAAGAAGTCAGAGAAAGAAAACAGAGTGGTAAA-<br>TATGAATATATGACATCTCTTATTGTAAAT<br>GGTAAGGATACATGGTCTGTAAAAGGATTAAAAAGTCATAAAGGTG-<br>TATATGATTATTCAAATCTGATTC<br>AGTTTGTTGAAAAGGAAAATAAATACCATCAGGCGAAAA-<br>TAATTTCTGAGCTCGGAGATAAAAATGATGT<br>TGTTTATTCTGGCTCAGGCTCGTCAGAAGTATTTGCTGGA-<br>GAAGGCCATGACACCGTATCTTATAATAAG<br>ACGGATGCTGGCAAACTAACAATTGATGCAACAGGAGCATCGAAAC-<br>CTGGTGAGTATATAGTTTCAAAAA<br>ATATGTATGGTGACGTGAGGGTATTGCAGGAAGTTGTTAATGAACAG-<br>GAGGTGTCAGTAGGAAAGCGAAC<br>AGAGAAAATACAATATCGTGATTTTGAATTCAGAGCCGGTG-<br>GAATTTCTTATGATGTGATAGATAAACTT<br>CATTCTGTTGAAGAACTCATCGGCG-<br>GAAAACATGATGATGAATTCAAAGGCGGTAAGTTTAATGATATAT<br>TCCATGGCGCAGATGGGAACGATTATATCGAAGGTAATTATGG-<br>TAATGATCGACTATACGGCGATGATGG<br>GGATGATTATATATCCGGAGGACAGGGCGACGACCAGTTATTTGGTGG-<br>TAGTGGAAACGATAAATTGAGT<br>GGAGGGGATGGTAATAATTATCTGACAGGAGGAAGCGG-<br>TAATGATGAGCTTCAGGCACACGGAGCTTATA<br>ATATTCTGTCAGGTGGCACTGGTGATGATAAACTTTATGGTGGTGG-<br>TATTGACCTTCTGGATGGAGGGGA<br>AGGTAATGACTATCTGAATGGCGGTTTTGGTAATGA-<br>TATTTATGTTTATCGGCAAAACTATGGTCATCAT<br>ACAATTGCAGATGAAGGAGGTAAAGGAGATCGCCTGCACTTACCTGA-<br>TATTAGCTTTGATGATATCGCAT<br>TTAAGAAAGTGGGAAATGATCTTATCATGAATAAAGCCATTAATGGTG-<br>TACTTTCATTTAATGAGTCAAA<br>TGATGTCAATGGGATAACATTTAAAAACTGGTTTGCGAAAGATGCCTCAG-<br>GAGCAGATAATCATCTTGTT<br>GAGATTATAACAGATAAAGATGGTCGTGAAATTAAATCTGATAACAT-<br>ACCTCATAAAAATAATGATTGGT<br>CAGGTTATATAAAAGCTAGTTCTATAGCATCAGAAAAAAATATGGTTAA-<br>TATCACCAGTGTTGCCAATGA<br>TATTAACAAAATTATTTCTTCAGTTTCAGGGGTAGATTCAGGTGATGAAC-<br>GATTAGCATCTTTATATAAT<br>TTAGCCTTACATCAAAATAACACACACTCAACAATTTTAAC-<br>GACAACTGTCTGA |
| CP012500.1 (09-00049) | O168 | D | Lettuce | ATGACAGTAAATAAAATAAAGAACATTTTCAGCAATGCGACATCGAC-<br>TACAAAATCAG-<br>CATTTAATACAGCATCATCAAACGTACGTACCGCTGGAAAAAAACTCATA<br>TTATTAATACCTGATGATTATGAAGCTCAGGGCGTGGGTATTAATGAGTT- |

GGTCAAA-
GCTGCTGATGAGCTTGGAATAGAAATACACCGTACTGAACGAGATGATAC
AGCGATTGCAAACCAGTTTTTGGTACAGCAGAAAAAGTTTTAGGAT-
TAACTGAAC-
GTGGTGTTGCAATATTCGCACCACAACTTGATAAACTTCTGCAGAAGTATC
AGAACGTTGGGAGTAAAATAGGCGGAACCGCTGAGAATGTAGGTAA-
TAATCTGGGAAAA-
GCCGGAACAGTTCTTTCAGCACTACATAATTTTACGGGGATTGCCTTATCA
GGCATGGCTCTTGATGAATTGCTGAGAAAACAACGGGGAGGAGAGGATA-
TAAGTCAGAATGA-
TATTGCCAAAAGTAGTATTGAACTTATTAATCACCTTGTAGATACAGTATC
AAGTATAAACAGTACTGTTGATTCATTTTCTGAGCAGCTTAACCAGCTT-
GGCTCATTTTTATCCAGTAAACCTAGCTTAAGTTCTGTTGGTGGAAAATTAC
AAAATTTACCTGACCTGGGCCCCTTGGGGGATGGGCTGGATGTTATCTCTG-
GAATTCTTTCAGCTGTATCAGCAAGCTTTATTCTGGGAAACAGTGACGCAC
ATACAGGAACAAAAGCAGCAG-
CAGGTATCGAACTGACAACTCAGGTTCTGGGAAATGTTGG-
TAAAGCTGTTTCACAATATATTCTGGCTCAGAGAATGGCACAGGGCTTATC
GACAACAGCTGCAAGTGCGGGTCTGATCACATCGGCTGTTATGCTGGC-
TATCAG-
TCCTCTTTCTTTCCTGGCTATTGCAGATAAATTTGAGCGAGCTAAGCAGCTT
GAAGCATATTCTGAACGATTTAAAAAATTGAATTATGAAGGG-
GATGCTTTACTT-
GCAGCCTTTCATAAAGAAACCGGAGCTATAGATGCAGCCCTGACAACAAT
AAATACTGTTCTGAGTTCTGTATCTGCGGGAGTTAGTGCAGCCTCCAGTG-
CATCCCTCATAGGGGCCCCGATAAGCATGCTAGTGAGTGCATTAACCGGT
ACGATATCTGGCATTCTGGAAGCATCAAAACAGGCTATGTTTGAGCAC-
GTTGCAGA-
TAAATTCGCTGCTCGGATCAATGAATGGGAAAAGGAGCATGGCAAAAATT
ATTTTGAGAATGGCTATGATGCAAGACATGCTGCATTTTTAGAA-
GACTCTCTGTCTTTGCTT-
GCAGATTTTTCTCGTCAGCATGCAGTAGAAAGAGCTGTCGCAATAACTCA
GCAACATTGGGATGAGAAGATCGGTGAACTTGCAGGTATAACCCG-
TAATGCTGATCGTAG-
TCAGAGTGGTAAGGCATATATTAATTATCTGGAGAATGGAGGGCTTTTAGA
GGATCAACCGAAGGAGTTTACACAACAAGTTTTT-
GATCCTCAAAAAGGGACCATAGAC-
CTTTCAACAGGTAATGTATCAAGTGTTTTGACATTTGTAACACCAACATTT
ACCCCAGGAGAAGAAGTCAGAGAAAGAAAACAGAGTGGTAAA-
TATGAATATATGACATCTCTTATTGTAAATGGTAAGGATACATGGTCTGTA
AAAGGATTAAAAAGTCATAAAGGTGTATATGATTATTCAAATCTGAT-
TCAGTTTGTT-
GAAAAGGATAATAAATACCATCAGGCGAAAATAATTTCTGAGCTCGGAG
ATAAAAATGATATTGTTTATTCTGGCTCAGGCTCGTCAGAAGTATTTGCTG-
GA-
GAAGGCCATGACACCGTATCTTATAATAAGACGGATGCTGGCAAACTAAC
AATTGATGCAACAGGAGCATCGAAACCTGGTGAGTATA-
TAGTTTCAAAAAA-
TATGTATGGTGACGTGAAGGTATTGCAGGAAGTCGTTAATGAACAGGAGG
TGTCAGTGGGAAAGCGAACAGAGAAAATACAATATCGTGATTTT-

| | | | | |
|---|---|---|---|---|
| CP012501.1 (08-00022) | O136 | D | Bagged lettuce | GAATTCAGAGCCGGTG-<br>GAATTTCTTATGATGTGATAGATAAGCTACATTCTGTTGAAGAACTCATCG<br>GCGGAAAACATGATGATGAATTCAAAGGCGGTAAGTTTAATGATA-<br>TATTCCATGGCG-<br>CAGATGGGAACGATTATATCGAAGGTAATTATGGTAATGATCGACTATAC<br>GGCGATGATGGGGATGATTATATATCCGGAGGACAGGGCGACGACCAG-<br>TTATTTGGTGGTAG-<br>TGGAAACGATAAATTGAGTGGAGGGGATGGTAATAATTATCTGACAGGAG<br>GAAGCGGTAATGATGAGCTTCAGGCACACGGAGCTTATAA-<br>TATTCTGTCAGGTGGCAC-<br>TGGTGATGATAAACTTTATGGTGGTGGTATTGACCTTCTGGATGGAGGGGA<br>AGGTAATGACTATCTGAATGGCGGTTTTGGTAATGA-<br>TATTTATGTTTATCGGCAAAAC-<br>TATGGTCATCATACAATTGCAGATGAAGGAGGTAAAGGAGATCGCCTGCA<br>CTTATCTGATATTAGCTTTGATGATATCGCATTTAAGAAAGTGG-<br>GAAATGATCTTATCATGAATAAAGCCATTAATGGTGTAATTTCATTTAATG<br>AGTCAAATGATGTCAATGGGATAACATTTAAAAACTGGTTTGCGAAA-<br>GATGCCTCAGGAG-<br>CAGATAATCATCTTGTTGAGATTATAACAGATAAAGATGGTCGTGAAATTA<br>AATCTGATAACATACCTCATAAAAATAATGATTGGTCAGGTTATATAAAA-<br>GCTAGTTC-<br>TATAGCATCAGAAAAAAATATGGTTAATATCACCAGTGTTGCCAATGATA<br>TTAACAAAATTATTTCTTCAGTTTCAGGGTTAGATTCAGGTGATGAAC-<br>GATTAGCATCTTTA-<br>TATAATTTAGCCTTACATCAAAATAACACACACTCAACAATTTTAACGACA<br>ACTGTCTGA<br>ATGACAGTAAATAAAATAAAGAACATTTTCAGCAATGCGACATCGAC-<br>TACAAAATCAG-<br>CATTTAATACAGCATCATCAAACGTACGTACCGCTGGAAAAAAACTCATA<br>TTATTAATACCTGATGATTATGAAGCTCAGGGCGTGGGTATTAATGAGTT-<br>GGTCAAA-<br>GCTGCTGATGAGCTTGGAATAGAAATACACCGTACTGAACGAGATGATAC<br>AGCGATTGCAAACCAGTTTTTTGGTACAGCAGAAAAAGTTTTAGGAT-<br>TAACTGAAC-<br>GTGGTGTTGCAATATTCGCACCACAACTTGATAAACTTCTGCAGAAGTATC<br>AGAACGTTGGGAGTAAAATAGGCGGAACCGCTGAGAATGTAGGTAA-<br>TAATCTGGGAAAA-<br>GCCGGAACAGTTCTTTCAGCACTACATAATTTTACGGGGATTGCCTTATCA<br>GGCATGGCTCTTGATGAATTGCTGAGAAAACAACGGGGAGGAGAGGATA-<br>TAAGTCAGAATGA-<br>TATTGCCAAAAGTAGTATTGAACTTATTAATCACCTTGTAGATACAGTATC<br>AAGTATAAACAGTACTGTTGATTCATTTTCTGAGCAGCTTAACCAGCTT-<br>GGCTCATTTTTATCCAGTAAACCTCGCTTAAGTTCTGTTGGTGGAAAATTAC<br>AAAATTTACCTGACCTGGGCCCCTTGGGGGATGGGCTGGATGTTATCTCTG-<br>GAATTCTTTCAGCTGTATCAGCAAGCTTTATTCTGGGAAACAGTGACGCAC<br>ATACAGGAACAAAAGCAGCAG-<br>CAGGTATCGAACTGACAACTCAGGTTCTGGGAAATGTTGG-<br>TAAAGCTGTTTCACAATATATTCTGGCTCAGAGAATGGCACAGGGCTTATC<br>GACAACAGCTGCAAGTGCGGGTCTGATCACATCGGCTGTTATGCTGGC-<br>TATCAG-<br>TCCTCTTTCTTTCCTGGCTATTGCAGATAAATTTGAGCGAGCTAAGCAGCTT |

GAAGCATATTCTGAACGATTTAAAAAATTGAATTATGAAGGG-
GATGCTTTACTT-
GCAGCCTTTCATAAAGAAACCGGAGCTATAGATGCAGCCCTGACAACAAT
AAATACTGTTCTGAGTTCTGTATCTGCGGGAGTTAGTGCAGCCTCCAGTG-
CATCCCTCATAGGGGCCCCGATAAGCATGCTAGTGAGTGCATTAACCGGT
ACGATATCTGGCATTCTGGAAGCATCAAAACAGGCTATGTTTGAGCAC-
GTTGCAGA-
TAAATTCGCTGCTCGGATCAATGAATGGGAAAAGGAGCATGGCAAAAATT
ATTTTGAGAATGGCTATGATGCAAGACATGCTGCATTTTTAGAA-
GACTCTCTGTCTTTGCTT-
GCAGATTTTTCTCGTCAGCATGCAGTAGAAAGAGCTGTCGCAATAACTCA
GCAACATTGGGATGAGAAGATCGGTGAACTTGCAGGTATAACCCG-
TAATGCTGATCGTAG-
TCAGAGTGGTAAGGCATATATTAATTATCTGGAGAATGGAGGGCTTTTAGA
GGATCAACCGAAGGAGTTTACACAACAAGTTTTT-
GATCCTCAAAAAGGGACCATAGAC-
CTTTCAACAGGTAATGTATCAAGTGTTTTGACATTTGTAACACCAACATTT
ACCCCAGGAGAAGAAGTCAGAGAAAGAAAACAGAGTGGTAAA-
TATGAATATATGACATCTCTTATTGTAAATGGTAAGGATACATGGTCTGTA
AAAGGATTAAAAAGTCATAAAGGTGTATATGATTATTCAAATCTGAT-
TCAGTTTGTT-
GAAAAGGATAATAAATACCATCAGGCGAAAATAATTTCTGAGCTCGGAG
ATAAAAATGATATTGTTTATTCTGGCTCAGGCTCGTCAGAAGTATTTGCTG-
GA-
GAAGGCCATGACACCGTATCTTATAATAAGACGGATGCTGGCAAACTAAC
AATTGATGCAACAGGAGCATCGAAACCTGGTGAGTATA-
TAGTTTCAAAAAA-
TATGTATGGTGACGTGAAGGTATTGCAGGAAGTCGTTAATGAACAGGAGG
TGTCAGTGGGAAAGCGAACAGAGAAAATACAATATCGTGATTTT-
GAATTCAGAGCCGGTG-
GAATTTCTTATGATGTGATAGATAAGCTACATTCTGTTGAAGAACTCATCG
GCGGAAAACATGATGATGAATTCAAAGGCGGTAAGTTTAATGATA-
TATTCCATGGCG-
CAGATGGGAACGATTATATCGAAGGTAATTATGGTAATGATCGACTATAC
GGCGATGATGGGGATGATTATATATCCGGAGGACAGGGCGACGACCAG-
TTATTTGGTGGTAG-
TGGAAACGATAAATTGAGTGGAGGGGATGGTAATAATTATCTGACAGGAG
GAAGCGGTAATGATGAGCTTCAGGCACACGGAGCTTATAA-
TATTCTGTCAGGTGGCAC-
TGGTGATGATAAACTTTATGGTGGTGGTATTGACCTTCTGGATGGAGGGGA
AGGTAATGACTATCTGAATGGCGGTTTTGGTAATGA-
TATTTATGTTTATCGGCAAAAC-
TATGGTCATCATACAATTGCAGATGAAGGAGGTAAAGGAGATCGCCTGCA
CTTATCTGATATTAGCTTTGATGATATCGCATTTAAGAAAGTGG-
GAAATGATCTTATCATGAATAAAGCCATTAATGGTGTACTATCATTTAATG
AGTCAAATGATGTCAATGGGATAACATTTAAAAACTGGTTTGCGAAA-
GATGCCTCAGGAG-
CAGATAATCATCTTGTTGAGATTATAACAGATAAAGATGGTCGTGAAATTA
AATCTGATAACATACCTCATAAAAATAATGATTGGTCAGGTTATATAAAA-
GCTAGTTC-
TATAGCATCAGAAAAAAATATGGTTAATATCACCAGTGTTGCCAATGATA

| | | | | |
|---|---|---|---|---|
| EF088504.1 (GZ-021210) | O157 | B | Cattle | TTAACAAAATTATTTCTTCAGTTTCAGGGTTAGATTCAGGTGATGAAC-<br>GATTAGCATCTTTA-<br>TATAATTTAGCCTTACATCAAAATAACACACACTCAACAATTTTAACGACA<br>ACTGTCTGA<br>ATGACAGTAAATAAAATAAAGAACATTTTCAATAATGCGACATTGAC-<br>TACAAAATCAGCATTTAATACAG<br>CATCATCAAGCGTACGTTCCGCTGGAAAAAAACTCATATTATTAATAC-<br>CTGATAATTATGAAGCTCAGGG<br>CGTGGGTATTAATGAGTTGGTCAAAGCTGCTGATGAGCTTGGAA-<br>TAGAAATACACCGTACTGAACGAGAT<br>GATACAGCGATTGCAAACCAGTTTTTGGTGCAGCAGAAAAAGTTGTAG-<br>GATTAACTGAACGTGGTGTTG<br>CAATATTCGCACCACAACTTGACAAACTTCTGCAGAAGTATCAGAAAGTT-<br>GGGAGTAAAATAGGAGGAAC<br>CGCTGAAAATGTAGGTAATAATCTGGGAAAAGCCGGAACAG-<br>TTCTCTCAGCACTACAGAATTTTACGGGG<br>ATTGCTTTATCAGGCATGGCTCTTGATGAATTGCTGAGAAAACAACGG-<br>GAAGGAGAGGATATAAGTCAGA<br>ATGATATTGCCAAAAGTAGTATTGAACTTATTAATCAGCTTGTAGATA-<br>CAGTATCAAGTATAAACAGTAC<br>CGTTGATTCATTTTCTGAGCAGCTTAACCAGCTTGGCTCATTTTTATCCAG-<br>TAAACCTCGATTAAGTTCT<br>GTTGGTGGGAAATTACAAAATTTACCAGACCTGGGCCCCCTGGGG-<br>GATGGGCTGGATGTTGTCTCCGGAA<br>TTCTTTCTGCTGTATCAGCAAGCTTTATTCTGGGAAACAGTGACGCACATA-<br>CAGGAACAAAAGCTGCAGC<br>GGGTATCGAACTGACAACTCAGGTTCTTGGAAATGTTGGTAAA-<br>GCTGTTTCGCAATATATTCTGGCTCAG<br>AGAATGGCACAGGGGTTATCGACAACAGCTG-<br>CAAGTGCGGGTCTGATCACATCGGCTGTTATGCTGGCTA<br>TCAGTCCTCTTTCTTTCCTGGCTGCTGCAGATAAATTTGAGCGAGCTAA-<br>GCAGCTTGAATCATATTCTGA<br>ACGATTTAAAAAATTGAATTATGAAGGGGATGCTTTACTCG-<br>CAGCCTTTCATAAAGAAACCGGAGCTATA<br>GATGCAGCCCTGACAACAATAAATACTGTCCTGAGTTCTGTATCTGCGG-<br>GAGTTAGTGCAGCCTCCAGTG<br>CATCCCTCATAGGGGCCCCGATAAGCATGCTGGTGAGTGCATTAACCGG-<br>TACGATATCTGGCATTCTGGA<br>AGCATCAAAACAGGCTATGTTTGAGCACGTTGCAGA-<br>GAAATTCGCTGCTCGGATCAATGAATGGGAAAAG<br>GAGCATGGCAAAAATTATTTTGAGAATGGATATGACGCAAGA-<br>CATGCTGCGTTTTTAGAAGACTCTCTGT<br>CTTTGCTTGCTGATTTTTCTCGTCAGCATGCAGTAGAAAGAGCAGTCGCAA-<br>TAACCCAGCAACATTGGGA<br>TGAGAAGATCGGTGAACTTGCAGGCATAACCCGTAATGCTGATCGCAG-<br>TCAGAGTGGTAAGGCATATATT<br>AATTATCTGGAAAATGGAGGGCTTTTAGAGGCTCAACCGAAGGAG-<br>TTTACACAACAAGTGTTTGATCCTC<br>AAAAAGGGACCATAGACCTTTCAACAGGTAATGTATCAAGTGTTTT-<br>GACATTTATAACACCAACATTTAC |

| | | | | |
|---|---|---|---|---|
| | | | | CCCAGGAGAAGAAGTTAGAGAAAGAAAACAGAGTGGTAAA-<br>TATGAATATATGACATCTCTTATTGTAAAT<br>GGTAAGGATACATGGTCTGTAAAAGGCATAAAAAATCATAAAGGTG-<br>TATATGATTATTCAAAATTGATTC<br>AGTTTGTTGAAAAGAATAACAAACACTATCAGGCGAGAA-<br>TAATTTCTGAGCTCGGAGATAAAGACGATGT<br>GGTTTATTCTGGAGCAGGCTCATCAGAAGTATTTGCTGGTGAAGGTTATGA-<br>TACCGTATCTTATAATAAG<br>ACGGATGTTGGTAAACTAACAATTGATGCAACAGGAGCATCAAAAC-<br>CTGGTGAGTATATAGTTTCAAAAA<br>ATATGTATGGTGACGTGAAGGTATTGCAGGAAGTCGTTAAGGAACAG-<br>GAGGTGTCAGTAGGGAAGCGAAC<br>AGAGAAAATACAATATCGTGATTTTGAATTCAGAACCGGTG-<br>GAATTCCTTATGATGTAATAGATAATCTT<br>CATTCTGTTGAAGAGCTCATTGGCG-<br>GAAAACATGATGATGAATTCAAAGGCGGTAAGTTTAATGATATAT<br>TCCATGGCGCAGATGGGAACGATTATATCGAAGGTAATTATGG-<br>TAATGATCGACTATACGGCGATGATGG<br>GGATGATTATATATCCGGAGGACAGGGAGACGACCAGTTATTTGGTGG-<br>TAGTGGAAACGATAAATTGAGT<br>GGAGGGGATGGTAATAATTATCTGACAGGAGGAAGCGG-<br>TAATGATGAGCTTCAGGCACACGGAGCTTATA<br>ATATTCTGTCAGGTGGTACTGGTGATGATAAACTTTATGGTGGTGGTGG-<br>TATTGATCTTCTGGATGGAGG<br>GGAAGGTAATGACTATCTGAATGGTGGTTTTGGTAATGA-<br>TATTTATGTTTATGGGCAAAACTATGGTCAT<br>CATACAATTGCAGATGAAGGAGGTAAAGGAGATCGTTTGCACTTATCTGA-<br>TATTAGCTTTGATGATATCG<br>CATTTAAGAGAGTTGGAAATGATCTTATCATGAATAAA-<br>GCCATTAATGGTGTACTTTCATTTAATGAGTC<br>AAATGATGTCAATGGGATAACATTTAAAAACTGGTTTGCGAAA-<br>GATGCCTCAGGAGCAGATAATCATCTT<br>GTTGAGGTTATAACAGATAAAGATGGTCGAGAGATAAAAGTTGATAAGA-<br>TACCTCATAATAATAATGAAC<br>GGTCAGGTTATATAAAAGCCAGTAA-<br>TATAGCATCTGAAAAAAACATGGTTAATATCACCAGTGTTACCAA<br>TGATATTAATAAGATTATTTCTTCAGTTTCAGGGTTCGAT-<br>TCAGGTGATGAACGATTAGCATCTTTATAT<br>AATTTATCCTTACATCAAAACAACACACACTCAACAACTTTAAC-<br>GACAACTGTCTGA |
| EF204919.1 (AGR047) | O90 | C | Cattle and sheep | ATGACAGTAAATAAAATAAAGAACATTTTCAATAATGCGACATTGAC-<br>TACAAAATCAGCATTTAATACAG<br>CATCATCAAGCGTACGTTCCGCTGGAAAAAAACTCATATTATTAATAC-<br>CTGATAATTATGAAGCTCAGGG<br>CGTGGGTATTAATGAGTTGGTCAAAGCTGCTGATGAGCTTGGAA-<br>TAGAAATACACCGTACTGAACGAGAT<br>GATACAGCGATTGCAAACCAGTTTTTTGGTGCAGCAGAAAAAGTTGTAG-<br>GATTAACTGAACGTGGTGTTG<br>CAATATTCGCACCACAACTTGACAAACTTCTGCAGAAGTATCAGAAAGTT-<br>GGGAGTAAAATAGGAGGAAC |

CGCTGAAAATGTAGGTAATAATCTGGGAAAAGCCGGAACAG-
TTCTCTCAGCACTACAGAATTTTACGGGG
ATTGCTTTATCAGGCATGGCTCTTGATGAATTGCTGAGAAAACAACGG-
GAAGGAGAGGATATAAGTCAGA
ATGATATTGCCAAAAGTAGTATTGAACTTATTAATCAGCTTGTAGATA-
CAGTATCAAGTATAAACAGTAC
CGTTGATTCATTTTCTGAGCAGCTTAACCAGCTTGGCTCATTTTTATCCAG-
TAAACCTCGCTTAAGTTCT
GTTGGTGGGAAATTACAAAATTTACCAGACCTGGGCTCCCTGGGG-
GATGGGCTGGATGTTGTCTCCGGAA
TTCTTTCTGCTGTATCAGCAAGCTTTATTCTGGGAAACAGTGACGCACATA-
CAGGAACAAAAGCTGCAGC
GGGTATCGAACTGACAACTCAGGTTCTTGGAAATGTTGGTAAA-
GCTGTTTCGCAATATATTCTGGCTCAG
AGAATGGCACAGGGGTTATCGACAACAGCTG-
CAAGTGCGGGTCTGATCACATCGGCTGTTATGCTGGCTA
TCAGTCCTCTTTCTTTCCTGGCTGCTGCAGATAAATTTGAGCGAGCTAA-
GCAGCTTGAATCATATTCTGA
ACGATTTAAAAAATTGAATTATGAAGGGGATGCTTTACTCG-
CAGCCTTTCATAAAGAAACCGGAGCTATA
GATGCAGCCCTGACAACAATAAATACTGTCCTGAGTTCTGTATCTGCGG-
GAGTTAGTGCAGCCTCCAGTG
CATCCCTCATAGGGGCCCCGATAAGCATGCTGGTGAGTGCATTAACCGG-
TACGATATCTGGCATTCTGGA
AGCATCAAAACAGGCTATGTTTGAGCACGTTGCAGA-
GAAATTCGCTGCTCGGATCAATGAATGGGAAAAG
GAGCATGGCAAAAAATTATTTTGAGAATGGCTATGACGCAAGA-
CATGCTGCGTTTTTAGAAGACTCTCTGT
CTTTGCTTGCTGATTTTTCTCGTCAGCATGCAGTAGAAAGAGCAGTCGCAA-
TAACCCAGCAACATTGGGA
TGAGAAGATCGGTGAACTTGCAGGCATAACCCGTAATGCTGATCGCAG-
TCAGAGTGGTAAGGCATATATT
AATTATCTGGAAAATGGAGGGCTTTTAGAGGCTCAACCGAAGGAG-
TTTACACAACAAGTGTTTGATCCTC
AAAAAGGGACCATAGACCTTTCAACAGGTAATGTATCAAGTGTTTT-
GACATTTATAACACCAACATTTAC
CCCAGGAGAAGAAGTTAGAGAAAGAAAACAGAGTGGTAAA-
TATGAATATATGACATCTCTTATTGTAAAT
GGTAAGGATACATGGTCTGTAAAAGGCATAAAAAATCATAAAGGTG-
TATATGATTATTCAAAATTGATTC
AATTTGTTGAAAAGAATAACAAACACTATCAGGCGAGAA-
TAATTTCTGAGCTCGGAGATAAAGACGATGT
GGTTTATTCTGGAGCAGGCTCATCAGAAGTATTT-
GCTGGTGAAGGTCATGATACCGTATCTTATAATAAG
ACGGATGTTGGTAAACTAACAATTGATGCAACAGGAGCATCAAAAC-
CTGGTGAGTATATAGTTTCAAAAA
ATATGTATGGTGACGTGAAGGTATTGCAGGAAGTCGTTAAGGAACAG-
GAGGTGTCAGTAGGGAAGCGAAC
AGAGAAAATACAATATCGTGATTTTGAATTCAGAACCGGTG-
GAATTCCTTATGATGTAATAGATAATCTT

| | | | | |
|---|---|---|---|---|
| EF204920.1 (AGR053) | O131 | C | Cattle and sheep | CATTCTGTTGAAGAGCTCATTGGCG- GAAAACATGATGATGAATTCAAAGGCGGTAAGTTTAATGATATAT TCCATGGCGCAGATGGGAACGATTATATCGAAGGTAATTATGG- TAATGATCGACTATACGGCGATGATGG GGATGATTATATATCCGGAGGACAGGGAGACGACCAGTTATTTGGTGG- TAGTGGAAACGATAAATTGAGT GGAGGGGATGGTAATAATTATCTGACAGGAGGAAGCGG- TAATGATGAGCTTCAGGCACACGGAGCTTATA ATATTCTGTCAGGTGGTACTGGTGATGATAAACTTTATGGTGGTGGTGG- TATTGATCTTCTGGATGGAGG GGAAGGTAATGACTATCTGAATGGTGGTTTTGGTAATGA- TATTTATGTTTATAGGCAAAACTATGGTCAT CATACAATTGCAGATGAAGGAGGTAAAGGAGATCGTTTGCACTTATCTGA- TATTAGCTTTGATGATATCG CATTTAAGAGAGTTGGAAATGATCTTATCATGAATAAA- GCCATTAATGGTGTACTTTCATTTAATGACTC AAATGATGTCAATGGGATAACATTTAAAAACTGGTTTGCGAAA- GATGCCTCAGGAGCAGATAATCATCTT GTTGAGGTTATAACAGATAAAGATGGTCGAGAGATAAAAGTTGATAAGA- TACCTCATAATAATAATGAAC GGTCAGGTTATATAAAAGCCAGTAA- TATAGCATCTGAAAAAAACATGGTTAATATCACCAGTGTTGCCAA TGATATTAATAAGATTATTTCTTCAGTTTCAGGGTTCGAT- TCAGGTGATGAACGATTAGCATCTTTATAT AATTTATCCTTACATCAAAACAACACACACTCAACAACTTTAAC- GACAACTGTCTGA ATGACAGTAAATAAAATAAAGAACATTTTCAATAATGCGACATTGAC- TACAAAATCAGCATTTAATACAG CATCATCAAGCGTACGTTCCGCTGGCAAAAAACTCATATTATTAATAC- CTGATAATTATGAAGCTCAGGG CGTGGGTATTAATGAGTTGGTCAAAGCTGCTGATGAGCTTGGAA- TAGAAATACACCGTACTGAACGAGAT GATACAGCGATTGCAAACCAGTTTTTTGGTGCAGCAGAAAAAGTTGTAG- GATTAACTGAACGTGGTGTTG CAATATTCGCACCACAACTTGACAAACTTCTGCAGAAGTATCAGAAAGTT- GGGAGTAAAATAGGAAGAAC CGCTGAAAATGTAGGTAATAATCTGGGAAAAGCCGGAACAG- TTCTCTCAGCACTACAGAATTTTACGGGG ATTGCTTTATCAGGCATGGCTCTTGATGAATTGCTGAGAAAACAACGG- GAAGGAGAGGATATAAGTCAGA ATGATATTGCCAAAAGTAGTATTGAACTTATTAATCAGCTTGTAGATA- CAGTATCAAGTATAAACAGTAC CGTTGATTCATTTTCTGAGCAGCTTAACCAGCTTGGCTCATTTTTATCCAG- TAAACCTCGCTTAAGTTCT GTTGGTGGGAAATTACAAAATTTACCAGACCTGGGCTCCCTGGGG- GATGGGCTGGATGTTGTCTCCGGAA TTCTTTCTGCTGTATCAGCAAGCTTTATTCTGGGAAACAGTGACGCACATA- CAGGAACAAAAGCTGCAGC GGGTATCGAACTGACAACTCAGGTTCTTGGAAATGTTGGTAAA- GCTGTTTCGCAATATATTCTGGCTCAG |

AGAATGGCACAGGGGTTATCGACAACAGCTG-
CAAGTGCGGGTCTGATCACATCGGCTGTTATGCTGGCTA
TCAGTCCTCTTTCTTTCCTGGCTGCTGCAGATAAATTTGAGCGAGCTAA-
GCAGCTTGAATCATATTCTGA
ACGATTTAAAAAATTGAATTATGAAGGGGATGCTTTACTCG-
CAGCCTTTCATAAAGAAACCGGAGCTATA
GATGCAGCCCTGACAACAATAAATACTGTCCTGAGTTCTGTATCTGCGG-
GAGTTAGTGCAGCCTCCAGTG
CATCCCTCATAGGGGCCCCGATAAGCATGCTGGTGAGTGCATTAACCGG-
TACGATATCTGGCATTCTGGA
AGCATCAAAACAGGCTATGTTTGAGCACGTTGCAGA-
GAAATTCGCTGCTCGGATCAATGAATGGGAAAAG
GAGCATGGCAAAAATTATTTTGAGAATGGCTATGACGCAAGA-
CATGCTGCGTTTTTAGAAGACTCTCTGT
CTTTGCTTGCTGATTTTTCTCGTCAGCATGCAGTAGAAAGAGCAGTCGCAA-
TAACCCAGCAACATTGGGA
TGAGAAGATCGGTGAACTTGCAGGCATAACCCGTAATGCTGATCGCAG-
TCAGAGTGGTAAGGCATATATT
AATTATCTGGAAAATGGAGGGCTTTTAGAGGCTCAACCGAAGGAG-
TTTACACAACAAGTGTTTGATCCTC
AAAAAGGGACCATAGACCTTTCAACAGGTAATGTATCAAGTGTTTT-
GACATTTATAACACCAACATTTAC
CCCAGGAGAAGAAGTTAGAGAAAGAAAACAGAGTGGTAAA-
TATGAATATATGACATCTCTTATTGTAAAT
GGTAAGGATACATGGTCTGTAAAAGGCATAAAAAAATCATAAAGGTG-
TATATGATTATTCAAAATTGATTC
AGTTTGTTGAAAAGAATAACAAACACTATCAGGCGAGAATGAT-
TTCTGAACTCGGAGATAAAGACGATGT
GGTTTATTCTGGAGCAGGCTCATCAGAAGTATTT-
GCTGGTGAAGGTCATGATACCGTATCTTATAATAAG
ACGGATGTTGGTAAACTAACAATTGATGCAACAGGAGCATCAAAAC-
CTGGTGAGTATATAGTTTCAAAAA
ATATGTATGGTGACGTGAAGGTATTGCAGGAAGTCGTTAAGGAACAG-
GAGGTGTCAGTAGGGAAGCGAAC
AGAGAAAATACAATATCGTGATTTTGAATTCAGAACCGGTG-
GAATTCCTTATGATGTAATAGATAATCTT
CATTCTGTTGAAGAGCTCATTGGCG-
GAAAACATGATGATGAATTCAAAGGCGGTAAGTTTAATGATATAT
TCCATGGCGCAGATGGGAACGATTATATCGAAGGTAATTATGG-
TAATGATCGACTATACGGCGATGATGG
GGATGATTATATATCCGGAGGACAGGGAGACGACCAGTTATTTGGTGG-
TAGTGGAAACGATAAATTGAGT
GGAGGGGATGGTAATAATTATCTGACAGGAGGAAGCGG-
TAATGATGAGCTTCAGGCACACGGAGCTTATA
ATATTCTGTCAGGTGGTACTGGTGATGATAAACTTTATGGTGGTGGTGG-
TATTGATCTTCTGGATGGAGG
GGAAGGTAATGACTATCTGAATGGTGGTTTTGGTAATGA-
TATTTATGTTTATAGGCAAAACTATGGTCAT
CATACAATTGCAGATGAAGGAGGTAAAGGAGATCGTTTGCACTTATCTGA-
TATTAGCTTTGATGATATCG

| | | | | |
|---|---|---|---|---|
| EF204921.1 (AGR119) | O153 | F | Cattle and sheep | CATTTAAGAGAGTTGGAAATGATCTTATCATGAATAAA-<br>GCCATTAATGGTGTACTTTCATTTAATGAGTC<br>AAATGATGTCAATGGGATAACATTTAAAAACTGGTTTGCGAAA-<br>GATGCCTCAGGAGCAGATAATCATCTT<br>GTTGAGGTTATAACAGATAAAGATGGTCGAGAGATAAAAGTTGATAAAA-<br>TACCTCATAATAATAATGAAC<br>GGTCAGGTTATATAAAAGCCAGTAA-<br>TATAGCATCTGAAAAAAACATGGTTAATATCACCAGTGTTGCCAA<br>TGATATTAATAAGATTATTTCTTCAGTTTCAGGGTTCGAT-<br>TCAGGTGATGAACGATTAGCATCTTTATAT<br>AATTTATCCTTACATCAAAACAACACACACTCAACAACTTTAAC-<br>GACAACTGTCTGA<br>ATGACAGTAAATAAAATAAAGAACATTTTCAATAATGCGACATTGAC-<br>TACAAAATCAGCATTTAATACAG<br>CATCATCAAGCGTACGTTCCGCTGGCAAAAAACTCATATTATTAATAC-<br>CTGATAATTATGAAGCTCAGGG<br>CGTGGGTATTAATGAGTTGGTCAAAGCTGCTGATGAGCTTGGAA-<br>TAGAAATACACCGTACTGAACGAGAT<br>GATACAGCGATTGCAAACCAGTTTTTGGTGCAGCAGAAAAAGTTGTAG-<br>GATTAACTGAACGTGGTGTTG<br>CAATATTCGCACCACAACTTGACAAACTTCTGCAGAAGTATCAGAAAGTT-<br>GGGAGTAAAATAGGAGGAAC<br>CGCTGAAAATGTAGGTAATAATCTGGGAAAAGCCGGAACAG-<br>TTCTCTCAGCACTACAGAATTTTACGGGG<br>ATTGCTTTATCAGGCATGGCTCTTGATGAATTGCTGAGAAAACAACGG-<br>GAAGGAGAGGATATAAGTCAGA<br>ATGATATTGCCAAAAGTAGTATTGAACTTATTAATCAGCTTGTAGATA-<br>CAGTATCAAGTATAAACAGTAC<br>CGTTGATTCATTTTCTGAGCAGCTTAACCAGCTTGGCTCATTTTTATCCAG-<br>TAAACCTCGCTTAAGTTCT<br>GTTGGTGGGAAATTACAAAATTTACCAGACCTGGGCTCCCTGGGG-<br>GATGGGCTGGATGTTGTCTCCGGAA<br>TTCTTTCTGCTGTATCAGCAAGCTTTATTCTGGGAAACAGTGACGCACATA-<br>CAGGAACAAAAGCTGCAGC<br>GGGTATAGAACTGACAACTCAGGTTCTTGGAAATGTTGGTAAA-<br>GCTGTTTCGCAATATATTCTGGCTCAG<br>AGAATGGCACAGGGGTTATCGACAACAGCTG-<br>CAAGTGCGGGTCTGATCACATCGGCTGTTATGCTGGCTA<br>TCAGTCCTCTTTCTTTCCTGGCTGCTGCAGATAAATTTGAGCGAGCTAA-<br>GCAGCTTGAATCATATTCTGA<br>ACGATTTAAAAAATTGAATTATGAAGGGGATGCTTTACTCG-<br>CAGCCTTTCATAAAGAAACCGGAGCTATA<br>GATGCAGCCCTGACAACAATAAATACTGTCCTGAGTTCTGTATCTGCGG-<br>GAGTTAGTGCAGCCTCCAGTG<br>CATCCCTCATAGGGGCCCCGATAAGCATGCTGGTGAGTGCATTAACCGG-<br>TACGATATCTGGCATTCTGGA<br>AGCATCAAAACAGGCTATGTTTGAGCACGTTGCAGA-<br>GAAATTCGCTGCTCGGATCAATGAATGGGAAAAG<br>GAGCATGGCAAAAATTATTTTGAGAATGGCTATGACGCAAGA-<br>CATGCTGCGTTTTTAGAAGACTCTCTGT |

| | | | | |
|---|---|---|---|---|
| | | | | CTTTGCTTGCTGATTTTTCTCGTCAGCATGCAGTAGAAAGAGCAGTCGCAA-<br>TAACCCAGCAACATTGGGA<br>TGAGAAGATCGGTGAACTTGCAGGCATAACCCGTAATGCTGATCGCAG-<br>TCAGAGTGGTAAGGCATATATT<br>AATTATCTGGAAAATGGAGGGCTTTTAGAGGCTCAACCGAAGGAG-<br>TTTACACAACAAGTGTTTGATCCTC<br>AAAAAGGGACCATAGACCTTTCAACAGGTAATGTATCAAGTGTTTT-<br>GACATTTATAACACCAACATTTAC<br>CCCAGGAGAAGAAGTTAGAGAAAGAAAACAGAGTGGTAAA-<br>TATGAATATATGACATCTCTTATTGTAAAT<br>GGTAAGGATACATGGTCTGTAAAAGGCATAAAAAATCATAAAGGTG-<br>TATATGATTATTCAAAATTGATTC<br>AGTTTGTTGAAAAGAATAACAAACACTATCAGGCGAGAA-<br>TAATTTCTGAGCTCGGAGATAAAGACGATGT<br>GGTTTATTCTGGAGCAGGCTCATCAGAAGTATTT-<br>GCTGGTGAAGGTCATGATACCGTATCTTATAATAAG<br>ACGGATGTTGGTAAACTAACAATTGATGCAACAGGAGCATCAAAAC-<br>CTGGTGAGTATATAGTTTCAAAAA<br>ATATGTATGGTGACGTGAAGGTATTGCAGGAAGTCGTTAAGGAACAG-<br>GAGGTGTCAGTAGGGAAGCGAAC<br>AGAGAAAATACAATATCGTGATTTTGAATTCAGAACCGGTG-<br>GAATTCCTTATGATGTAATAGATAATCTT<br>CATTCTGTTGAAGAGCTCATTGGCG-<br>GAAAACATGATGATGAATTCAAAGGCGGTAAGTTTAATGATATAT<br>TCCATGGCGCAGATGGGAACGATTATATCGAAGGTAATTATGG-<br>TAATGATCGACTATACGGCGATGATGG<br>GGATGATTATATATCCGGAGGACAGGGAGACGACCAGTTATTTGGTGG-<br>TAGTGGAAACGATAAATTGAGT<br>GGAGGGGATGGTAATAATTATCTGACAGGAGGAAGCGG-<br>TAATGATGAGCTTCAGGCACACGGAGCTTATA<br>ATATTCTGTCAGGTGGTACTGGTGATGATAAACTTTATGGTGGTGGTGG-<br>TATTGATCTTCTGGATGGAGG<br>GGAAGGTAATGACTATCTGAATGGTGGTTTTGGTAATGA-<br>TATTTATGTTTATAGGCAAAACTATGGTCAT<br>CATACAATTGCAGATGAAGGAGGTAAAGGAGATCGTTTGCACTTATCTGA-<br>TATTAGCTTTGATGATATCG<br>CATTTAAGAGAGTTGGAAATGATCTTATCATGAATAAA-<br>GCCATTAATGGTGTACTTTCATTTAATGAGTC<br>AAATGATGTCAATGGGATAACATTTAAAAACTGGTTTGCGAAA-<br>GATGCCTCAGGAGCAGATAATCATCTT<br>GTTGAGGTTATAACAGATAAAGATGGTCGAGAGATAAAAGTTGATAAAA-<br>TACCTCATAATAATAATGAAC<br>GGTCAGGTTATATAAAAGCCAGTAA-<br>TATAGCATCTGAAAAAAACATGGTTAATATCACCAGTGTTGCCAA<br>TGATATTAATAAGATTATTTCTTCAGTTTCAGGATTCGAT-<br>TCAGGTGATGAACGATTAGCATCTTTATAT<br>AATTTATCCTTACATCAAAACAACACACACTCAACAACTTTAAC-<br>GACAACTGTCTGA |
| EF2049<br>22.1 | O98 | A | Cattle<br>and<br>sheep | ATGACAGTAAATAAAATAAAGAACATTTTCAACAATGCGACATCGAC-<br>TACAAAATCAGCATTTAATACAG |

(AGR1 51)

CATCATCAAGCGTACGTTCCGCTGGAAAAAAACTCATATTATTAATAC-
CTGATAATTATGAAGCTCAGGG
CGTGGGTATTAATGAGTTGGTCAAAGCTGCTGATGAGCTTGGAA-
TAGAAATACACCGTACTGAACGAGAT
GATACAGCGATTGCAAACCAGTTTTTGGTACAGCAGAAAAAGTTGTAG-
GATTAACTGAACGTGGTGTTG
CAATATTCGCACCACAACTTGACAAACTTCTGCAGAAGTATCAGAAAGTT-
GGGAGTAAAATAGGAGGAAC
CGCTGAAAATGTAGGTAATAATCTGGGAAAAGCCGGAACAG-
TTCTCTCAGCACTACAGAATTTTACGGGG
ATTGCTTTATCAGGCATGGCTCTTGATGAATTGCTGAGAAAACAACGG-
GAAGGAGAGGATATAAGTCAGA
ATGATATTGCCAAAAGTAGTATTGAACTTATTAATCAGCTTGTAGATA-
CAGTATCAAGTATAAACAGTAC
CGTTGATTCATTTTCTGAGCAGCTTAACCAGCTTGGCTCATTTTTATCCAG-
TAAACCTCGCTTAAGTTCT
GTTGGTGGAAAATTACAAAATTTACCAGACCTGGGCCCCCTGGGG-
GATGGGCTGGATGTTGTCTCCGGAA
TTCTTTCTGCTGTATCAGCAAGTTTTATTCTGGGAAACAGTGACGCACATA-
CAGGAACAAAAGCTGCAGC
GGGTATCGAACTGACAACTCAGGTTCTTGGAAATGTTGGTAAA-
GCTGTTTCGCAATATATTCTGGCTCAG
AGAATGGCACAGGGATTATCGACAACAGCTG-
CAAGTGCGGGTCTGATCACATCGGCTGTTATGCTGGCTA
TCAGTCCTCTTTCTTTCCTGGCTATTGCAGATAAATTTGAGCGAGCTAA-
GCAGCTTGAATCATATTCTGA
ACGATTTAAAAAATTCAATTATGAAGGGGATGCTTTACTCG-
CAGCCTTTCATAAAGAAAGCGGAGCTATA
GATGCAGCCCTGACAACAATAAATACTGTCCTGAGTTCTGTATCTGCGG-
GAGTTAGTGCAGCCTCCAGTG
CATCCCTCATAGGGGCCCCGATAAGCATGCTGGTGAGTGCATTAACCGG-
TACGATATCTGGCATTCTGGA
TGCATCAAAACAGGCTATGTTTGAGCACGTTGCAGA-
TAAATTCGCTGCTCGGATCAATGAATGGGAAAAG
GAGCATGGCAAAAATTATTTTGAGAATGGCTATGACGCAAGA-
CATGCTGCGTTTTTAGAAGACTCTCTGT
CTTTGCTTGCTGATTTTTCTCGTCAGCATGCAGTAGAAAGAGCTGTCGCAA-
TAACCCAGCAACATTGGGA
TGAGAAGATCGGTGAACTTGCAGGTATAACCCGTAATGCTGATCGCAG-
TCAGAGTGGTAAGGCATATATT
AATTATCTGGAGAATGGAGGGCTTTTAGAGGCTCAACCGAAGGAG-
TTTACACAACAAGTTTTTGATCCTC
AAAAAGGGACTATAGACCTTTCAACAGGTAATGTATCAAGTGTTTT-
GACATTTGTAACACCAACATTTAC
CCCAGGAGAAGAAGTCAGAGAAAGAAAACAGAGTGGTAAA-
TATGAATATATGACATCTCTTATTGTAAAT
GGTAAGGATACATGGTCTGTAAAAGGCATAAAAAATCATAAAGGTG-
TATATGATTATTCAAATTTGATTC
AGTTTGTTGAAAAGGATAACAAACACTATCAGGCGAGAA-
TAATTTCTGAGCTCGGAGATAAAGACGATAT

| | | | | |
|---|---|---|---|---|
| EF2049 23.1 (AGR1 58) | O101 | E | Cattle and sheep | AGTTTATTCTGGGGCAGGCTCATCAGAAGTATTT-GCTGGTGAAGGTCATGATACCGTATCTTATAATAAG ACGGATGTTGGTAAACTAACAATTGATGCAACAGGAGCATCAAAAC-CTGGTGAATATATAGTTTCAAAAA ATATGTATGGTGACGTGAAGGTATTGCAGGAAGTCGTTAAGGAACAG-GAGGTGTCAGTAGGGAAGAGAAC AGAGAAAATACAATATCGTGATTTTGAATTCAGAACCGGTG-GAATTCCTTATGATGTGATAGATAATCTT CATTCTGTTGAAGAACTCATTGGCG-GAAAACATGATGATGAATTCAAAGGCGGTAAGTTTAATGATATAT TCCATGGTGCAGATGGGAACGATTATATCGAAGGTAATTATGG-TAATGATCGACTATACGGCGATGATGG GGATGATTATATATCCGGAGGACAGGGAGACGACCAGTTATTTGGTGG-TAGTGGAAATGATAAATTGAGT GGAGGGGATGGTAATAATTATCTGACAGGAGGAAGCGG-TAATGATGAGCTTCAGGCACACGGAGCTTATA ATATTCTGTCAGGTGGTACTGGTGATGATAAACTTTATGGTGGTGGTGG-TATTGACCTTCTGGATGGAGG GGAAGGTAATGACTATCTGAATGGTGGTTTTGGTAATGA-TATTTATGTTTATAGGCAAAACTATGGTCAT CATACAATTGCAGATGAAGGAGGTAAAGGAGATCGTCTGCACTTATCTGA-TATTAGCTTTGATGATATCG CATTTAAGAAAGTTGGAAATGATCTTATCATGAATAAA-GCCATTAATGGTGCACTTTCATTTAATGAGTC AAATGATGTCAATGGGATAACATTTAAAAACTGGTTTGCGAAA-GATGCCTCAGGTGAAGATAATCATCTT GTTGAGGTTATAACAGATAAAGATGGTCGTGAGATAAAAGCTGATAAGA-TATCTCATAATAATAATGAAC AGTCAGGTTATATAAAACCCAGTAA-TATAGCATCTGAAAAAAACATGGTTAATATCACCAGTGTTGCCAA TGATATTAACAAGATTATTTCTTCAGTTTCAGGGTTCGAT-TCAGGTGATGAACGATTAGCATCTTTATAT AATTTATCTTTACATCAAAATAACACACACTCAACAACTTTAAC-GACAACTGTCTGA ATGACAGTAAATAAAATAAAGAACATTTTCAATAATGCTGCATTGAC-TACAAAATCAGCATTTAATACAG CATCATCAAGCGTACGTTCCGCTGGAAAAAAACTCATATTATTAATAC-CTGATAATTATGAAGCTCAGGG CGTGGGTATTAATGAGTTGGTCAAAGCTGCTGATGAGCTTGGAA-TAGAAATACACCGTACTGAACGAGAT GATACAGCGATTGCAAACCAGTTTTTTGGTACAGCAGAAAAAGTTGTAG-GATTAACTGAACGTGGTGTTG CAATATTCGCACCACAACTTGACAAACTTCTGCAGAAGTATCAGAAAGTT-GGGAGTAAAATAGGAGGAAC CGCTGAAAATGTAGGTAATAATCTGGGAAAAGCCGGAACAGTTCTCTCAC-CACTACAGAATTTTACGGGG ATTGCTTTATCAGGCATGGCTCTTGATGAATTGCTGAGAAAACAACGG-GAAGGAGAGGATATAAGTCAGA ATGATATTGCCAAAAGTAGTATTGAACTTATTAATCAACTTGTAGATA-CAGTATCAAGTATAAACAGTAC |

CGTTGATTCATTTTCTGAGCAGCTTAACCAGCTTGGCTCATTTTTATCCAG-
TAAACCTCGCTTAAGTTCT
GTTGGTGGAAAATTACAAAATTTACCGGACCTGGGCCCCTTGGGG-
GATGGGCTGGATGTTGTCTCCGGAA
TTCTTTCTGCTGTATCAGCAAGCTTTATTCTGGGAAACAGTGACGCACATA-
CAGGAACAAAAGCTGCAGC
GGGTATCGAACTGACAACTCAGGTTCTTGGAAATGTTGGTAAA-
GCTGTTTCGCAATATATTCTGGCTCAG
AGAATAGCACAGGGGTTATCGACAACAGCTG-
CAAGTGCGGGTCTGATCACATCGGCTGTTATGCTGGCTA
TCAGTCCTCTTTCTTTCCTGGCTATTGCAGATAAATTTGAGCGAGCTAA-
GCAGCTTGAATCATATTCTGA
ACGATTTAAAAAATTGAATTATGAAGGGGATGCTTTACTCG-
CAGCCTTTCATAAAGAAACCGGAGCTATA
GATGCAGCCCTGACAACAATAAATACTGTCCTGAGTTCTGTATCTGCGG-
GAGTTAGTGCAGCCTCCAGTG
CATCCCTCATAGGGGCCCCGATAAGCATGCTGGTGAGTGCATTAACCGG-
TACGATATCTGGCATTCTGGA
AGCATCAAAACAGGCTATGTTTGAGCACGTTGCAGA-
TAAATTCGCTGCTCGGATCAATGAATGGGAAAAG
GAGCATGGCAAAAATTATTTTGAGAATGGCTATGACGCAAGA-
CATGCTGCGTTTTTAGAAGATTCTCTGT
CTTTGCTTGCTGATTTTTCTCGTCAGCATGCAGTAGAAAGAGCTGTCGCAA-
TAACCCAGCAACATTGGGA
TGAGAAGATCGGTGAACTTGCAGGTATAACCCGTAATGCTGATCGCAG-
TCAGAGTGGTAAGGCATATATT
AATTATCTGGAGAATGGAGGGCTTTTAGCGGCTCAACCGAAGGAG-
TTTACACAACAAGTTTTTGATCCTC
AAAAAGGGACTATAGACCTTTCAACAGGTAATGTATCAAGTGTTTT-
GACATTTGTAACACCAACATTTAC
CCCAGGAGAAGAAGTCAGAGAAAGAAAACAGAGTGGTAAA-
TATGAATATATGACATCTCTTATTGTAAAT
GGTAAGGATACATGGTCTGTAAAAGGCATAAAAAATCATAAAGGTG-
TATATGATTATTCAAATTTGATTC
AGTTTGTTGAAAAGGATAACAAACACTATCAGGCGAGAA-
TAATTTCTGAGCTCGGAGATAAAGACGATAT
AGTTTATTCTGGGGCTGGCTCATCAGAAGTATTTGCTGGTGAAGGTCATGA-
TACCGTATCTTATAATAAG
ACGGATGTTGGTAAACTAACAATTGATGCAACAGGAGCATCAAAAC-
CTGGTGAATATATAGTTTCAAAAA
ATATGTATGGTGACGTGAAGGTATTGCAGGAAGTCGTTAAGGAACAG-
GAGGTGTCAGTAGGGAAGCGAAC
AGAGAAAATACAATATCGTGATTTTGAATTCAGAACCGGTG-
GAATTCCTTATGATGTGATAGATAATCTT
CATTCTGTTGAAGAACTCATTGGCG-
GAAAACATGATGATGAATTCAAAGGCGGTAAGTTTAATGATATAT
TCCATGGCGCAGATGGGAACGATTATATCGAAGGTAATTATGG-
TAATGATCGACTATACGGCGATGATGG
GGATGATTATATATCCGGAGGACAGGGAGACGACCAGTTATTTGGTGG-
TAGTGGAAACGATAAATTGAGT

| | | | | |
|---|---|---|---|---|
| EF2049<br>24.1<br>(AGR2<br>70) | O121 | B | Cattle<br>and<br>sheep | GGAGGGGATGGTAATAATTATCTGACAGGAGGAAGCGG-<br>TAATGATGAGCTTCAGGCACACGGAGCTTATA<br>ATATTCTGTCAGGTGGTACTGGTGATGATAAACTTTATGGTGGTGGTGG-<br>TATTGACCTTCTGGATGGAGG<br>GGAAGGTAATGACTATCTGAATGGTGGTTTTGGTAATGA-<br>TATTTATGTTTATAGGCAAAACTATGGTCAT<br>CATACAATTGCAGATGAAGGAGGTAAAGGAGATCGTCTGCACTTATCTGA-<br>TATTAGCTTTGATGATATCG<br>CATTTAAGAGAGTTGGAAATGATCTTATCATGAATAAA-<br>GCCATTAATGGTGTACTTTCATTTAATGAGTC<br>AAATGATGTCAATGGGATAACATTTAAAAACTGGTTTGCGAAA-<br>GATGCCTCAGGAGCAGATAATCATCTT<br>GTTGAGGTTATAACAGATAAAGATGGTCGTGAGATAAAAGCTGATAAGA-<br>TACCTCATAATAATAATGAAC<br>GGTCAGGTTATATAAAAGCCAGTAA-<br>TATAGCATCTGAAAAAAACATGGTTAATATCACCAGTGTTGCCGA<br>TGATATTAACAAGATTATTTCTTCAGTTTCAGGGTTCGAT-<br>TCAGGTGATGAACGATTAGCATCTTTATAT<br>AATTTATCTTTACATCAAAACAACACACACTCAACAACTTTAAC-<br>GACAACTGTCTGA<br>ATGACAGTAAATAAAATAAAGAACATTTTCAATAATGCGACATTGAC-<br>TACAAAATCAGCATTTAATACAG<br>CATCATCAAGCGTACGTTCCGCTGGAAAAAAACTCATATTATTAATAC-<br>CTGATAATTATGAAGCTCAGGG<br>CGTGGGTATTAATGAGTTGGTCAAAGCTGCTGATGAGCTTGGAA-<br>TAGAAATACACCGTACTGAACGAGAT<br>GATACAGCGATTGCAAACCAGTTTTTGGTGCAGCAGAAAAAGTTGTAG-<br>GATTAACTGAACGTGGTGTTG<br>CAATATTCGCACCACAACTTGACAAACTTCTGCAGAAGTATCAGAAAGTT-<br>GGGAGTAAAATAGGAGGAAC<br>CGCTGAAAATGTAGGTAATAATCTGGGAAAAGCCGGAACAG-<br>TTCTCTCAGCACTACAGAATTTTACGGGG<br>ATTGCTTTATCAGGCATGGCTCTTGATGAATTGCTGAGAAAACAACGG-<br>GAAGGAGAGGATATAAGTCAGA<br>ATGATATTGCCAAAAGTAGTATTGAACTTATTAATCAGCTTGTAGATA-<br>CAGTATCAAGTATAAACAGTAC<br>CGTTGATTCATTTTCTGAGCAGCTTAACCAGCTTGGCTCATTTTTATCCAG-<br>TAAACCTCGATTAAGTTCT<br>GTTGGTGGGAAATTACAAAATTTACCAGACCTGGGCCCCCTGGGG-<br>GATGGGCTGGATGTTGTCTCCGGAA<br>TTCTTTCTGCTGTATCAGCAAGCTTTATTCTGGGAAACAGTGACGCACATA-<br>CAGGAACAAAAGCTGCAGC<br>GGGTATCGAACTGACAACTCAGGTTCTTGGAAATGTTGGTAAA-<br>GCTGTTTCGCAATATATTCTGGCTCAG<br>AGAATGGCACAGGGGTTATCGACAACAGCTG-<br>CAAGTGCGGGTCTGATCACATCGGCTGTTATGCTGGCTA<br>TCAGTCCTCTTTCTTTCCTGGCTGCTGCAGATAAATTTGAGCGAGCTAA-<br>GCAGCTTGAATCATATTCTGA<br>ACGATTTAAAAAATTGAATTATGAAGGGGATGCTTTACTCG-<br>CAGCCTTTCATAAAGAAACCGGAGCTATA |

GATGCAGCCCTGACAACAATAAATACTGTCCTGAGTTCTGTATCTGCGG-
GAGTTAGTGCAGCCTCCAGTG
CATCCCTCATAGGGGCCCCGATAAGCATGCTGGTGAGTGCATTAACCGG-
TACGATATCTGGCATTCTGGA
AGCATCAAAACAGGCTATGTTTGAGCACGTTGCAGA-
GAAATTCGCTGCTCGGATCAATGAATGGGAAAAG
GAGCATGGCAAAAATTATTTGAGAATGGATATGACGCAAGA-
CATGCTGCGTTTTTAGAAGACTCTCTGT
CTTTGCTTGCTGATTTTTCTCGTCAGCATGCAGTAGAAAGAGCAGTCGCAA-
TAACCCAGCAACATTGGGA
TGAGAAGATCGGTGAACTTGCAGGCATAACCCGTAATGCTGATCGCAG-
TCAGAGTGGTAAGGCATATATT
AATTATCTGGAAAATGGAGGGCTTTTAGAGGCTCAACCGAAGGAG-
TTTACACAACAAGTGTTTGATCCTC
AAAAAGGGACCATAGACCTTTCAACAGGTAATGTATCAAGTGTTTT-
GACATTTATAACACCAACATTTAC
CCCAGGAGAAGAAGTTAGAGAAAGAAAACAGAGTGGTAAA-
TATGAATATATGACATCTCTTATTGTAAAT
GGTAAGGATACATGGTCTGTAAAAGGCATAAAAAATCATAAAGGTG-
TATATGATTATTCAAAATTGATTC
AGTTTGTTGAAAAGAATAACAAACACTATCAGGCGAGAA-
TAATTTCTGAGCTCGGAGATAAAGACGATGT
GGTTTATTCTGGAGCAGGCTCATCAGAAGTATTTGCTGGTGAAGGTTATGA-
TACCGTATCTTATAATAAG
ACGGATGTTGGTAAACTAACAATTGATGCAACAGGAGCATCAAAAC-
CTGGTGAGTATATAGTTTCAAAAA
ATATGTATGGTGACGTGAAGGTATTGCAGGAAGTCGTTAAGGAACAG-
GAGGTGTCAGTAGGGAAGCGAAC
AGAGAAAATACAATATCGTGATTTTGAATTCAGAACCGGTG-
GAATTCCTTATGATGTAATAGATAATCTT
CATTCTGTTGAAGAGCTCATTGGCG-
GAAAACATGATGATGAATTCAAAGGCGGTAAGTTTAATGATATAT
TCCATGGCGCAGATGGGAACGATTATATCGAAGGTAATTATGG-
TAATGATCGACTATACGGCGATGATGG
GGATGATTATATATCCGGAGGACAGGGAGACGACCAGTTATTTGGTGG-
TAGTGGAAACGATAAATTGAGT
GGAGGGGATGGTAATAATTATCTGACAGGAGGAAGCGG-
TAATGATGAGCTTCAGGCACACGGAGCTTATA
ATATTCTGTCAGGTGGTACTGGTGATGATAAACTTTATGGTGGTGGTGG-
TATTGATCTTCTGGATGGAGG
GGAAGGTAATGACTATCTGAATGGTGGTTTTGGTAATGA-
TATTTATGTTTATGGGCAAAACTATGGTCAT
CATACAATTGCAGATGAAGGAGGTAAAGGAGATCGTTTGCACTTATCTGA-
TATTAGCTTTGATGATATCG
CATTTAAGAGAGTTGGAAATGATCTTATCATGAATAAA-
GCCATTAATGGTGTACTTTCATTTAATGAGTC
AAATGATGTCAATGGGATAACATTTAAAAACTGGTTTGCGAAA-
GATGCCTCAGGAGCAGATAATCATCTT
GTTGAGGTTATAACAGATAAAGATGGTCGAGAGATAAAAGTTGATAAGA-
TACCTCATAATAATAATGAAC

| | | | | |
|---|---|---|---|---|
| EF204925.1 (AGR340) | O91 | A | Cattle and sheep | GGTCAGGTTATATAAAAGCCAGTAA-<br>TATAGCATCTGAAAAAAACATGGTTAATATCACCAGTGTTGCCAA<br>TGATATTAATAAGATTATTTCTTCAGTTTCAGGGTTCGAT-<br>TCAGGTGATGAACGATTAGCATCTTTATAT<br>AATTTATCCTTACATCAAAACAACACACACTCAACAACTTTAAC-<br>GACAACTGTCTGA<br>ATGACAGTAAATAAAATAAAGAACATTTTCAACAATGCGACATCGAC-<br>TACAAAATCAGCATTCAATACAG<br>CATCATCAAGCGTACGTTCCGCTGGAAAAAAACTCATATTATTAATAC-<br>CTGATAATTATGAAGCTCAGGG<br>CGTGGGTATTAATGAGTTGGTCAAAGCTGCTGATGATCTTGGAA-<br>TAGAAATACACCGTACTGAACGAGAT<br>GATACAGCGATTGCAAACCAGTTTTTGGTACAGCAGAAAAAGTTGTAG-<br>GATTAACTGAACGTGGTGTTG<br>CAATATTCGCACCACAACTTGACAAACTTCTGCAGAAGTATCAGAAAGTT-<br>GGGAGTAAAATAGGAGGAAC<br>CGCTGAAAATGTAGGTAATAATCTGGGAAAAGCCGGAACAG-<br>TTCTCTCAGCACTACAGAATTTTACGGGG<br>ATTGCTTTATCTGGCATGGCTCTTGATGAATTGCTGAGAAAACAACGG-<br>GAAGGAGAGGATATAAGTCAGA<br>ATGATATTGCCAAAAGTAGTATTGAACTTATTAATCAGCTTGTAGATA-<br>CAGTATCAAGTATAAACAGTAC<br>CGTTGATTCATTTTCTGAGCAGCTTAACCAGCTTGGCTCATTTTTATCCAG-<br>TAAACCTCGCTTAAGTTCT<br>GTTGGTGGAAAATTACAAAATTTACCGAACCTGGGCCCCCTGGGG-<br>GATGGGCTGGATGTTGTCTCCGGAA<br>TTCTTTCTGCTGTATCAGCAAGCTTTATTCTGGGAAACAGTGACGCACATA-<br>CAGGAACAAAAGCTGCAGC<br>GGGTATCGAACTGACAACTCAGGTTCTTGGAAATGTTGGTAAA-<br>GCTGTTTCGCAATATATTCTGGCTCAG<br>AGAATGGCACAGGGATTATCGACAACAGCTG-<br>CAAGTGCGGGTCTGATCACATCGGCTGTTATGCTGGCTA<br>TCAGTCCTCTTTCTTTCCTGGCTATTGCAGATAAATTTGAGCGAGCTAA-<br>GCAGCTTGAATCATATTCTGA<br>ACGATTTAAAAAATTGAATTATGAAGGGGATGCTTTACTCG-<br>CAGCCTTTCATAAAGAAACCGGAGCTATA<br>GATGCAGCCCTGACAACAATAAATACTGTCCTGAGTTCTGTATCTGCGG-<br>GAGTTAGTGCCGCCTCCAGTG<br>CATCCCTCATAGGGGCCCCGATAAGCATGCTGGTGAGTGCATTAACCGG-<br>TACGATATCTGGCATTCTGGA<br>AGCATCAAAACAGGCTATGTTTGAGCACGTTGCAGA-<br>TAAATTCGCTGCCCGGATCAATGAATGGGAAAAG<br>GAGCATGGCAAAAATTATTTTGAGAATGGCTATGACGCAAGA-<br>CATGCTGCGTTTTTAGAAGATTCTCTGT<br>CTTTGCTTGCTGATTTTTCTCGTCAGCATGCAGTAGAAAGAGCTGTCG-<br>CAATCACCCAGCAACATTGGGA<br>TGAGAAGATCGGTGAACTTGCAGGTATAACCCGTAATGCTGATCGCAG-<br>TCAGAGTGGTAAGGCATATATT<br>AATTATCTGGAGAATGGAGGGCTTTTAGAGGCTCAACCGAAGGAG-<br>TTTACACAACAAGTTTTGATCCTC |

| | | | | |
|---|---|---|---|---|
| EF204927.1 (AGR670) | ONT | D | Cattle and sheep | AAAAAGGGACTATAGACCTTTCAACAGGTAATGTATCAAGTGTTTT-<br>GACATTTGTAACACCAACATTTAC<br>CCCAGGAGAAGAAGTCAGAGAAAGAAAACAGAGTGGTAAA-<br>TATGAATATATGACATCTCTTATTGTAAAT<br>GGTAAGGATACATGGTCTGTAAAAGGCATAAAAAATCATAAAGGTG-<br>TATATGATTATTCAAATTTGATTC<br>AGTTTGTTGAAAAGGATAACAAACACTATCAGGCGAGAA-<br>TAATTTCTGAGCTCGGAGATAAAGACGATAT<br>AGTTTATTCTGGGGCAGGCTCATCAGAAGTATTT-<br>GCTGGTGAAGGTCATGATACCGTATCTTATAATAAG<br>ACGGATGTTGGTAAACTAACAATTGATGCAACAGGAGCATCAAAAC-<br>CTGGTGAATATATAGTTTCAAAAA<br>ATATGTATGGTGACGTGAAGGTATTGCAGGAAGTCGTTAAGGAACAG-<br>GAGGTGTCAGTAGGGAAGCGAAC<br>AGAGAAAATACAATATCGTGATTTTGAATTCAGAACCGGTG-<br>GAATTCCTTATGATGTGATAGATAATCTT<br>CATTCTGTTGAAGAACTCATTGGCG-<br>GAAAACATGATGATGAATTCAAAGGCGGTAAGTTTAATGATATAT<br>TCCATGGCGCAGATGGGAACGATTATATCGAAGGTAATTATGG-<br>TAATGATCGACTATACGGCGATGATAG<br>GGATGATTATATATCCGGAGGACAGGGAGACGACCAGTTATTTGGTGG-<br>TAGTGGAAACGATAAATTGAGT<br>GGAGGGGATGGTAATAATTATCTGACAGGAGGAAGCGG-<br>TAATGATGAGCTTCAGGCACACGGAGCTTATA<br>ATATTCTGTCAGGTGGTACTGGTGATGATAAACTTTATGGTGGTGGTGG-<br>TATTGACCTTCTGGATGGAGG<br>GGAAGGTAATGACTATCTGAATGGTGGTTTTGGTAATGA-<br>TATTTATGTTTATAGGCAAAACTATGGTCAT<br>CATACAATTGCAGATGAAGGAGGTAAAGGAGATCGTCTGCACTTATCTGA-<br>TATTAGCTTTGATGATATCG<br>CATTTAAGAGAGTTGGAAATGATCTTATCATGAATAAA-<br>GCCATTAATGGTGTACTTTCATTTAATGAGTC<br>AAATGATGTCAATGGGATAACATTTAAAAACTGGTTTGCGAAA-<br>GATGCCTCAGGAGCAGATAATCATCTT<br>GTTGAGGTTATAACAGATAAAGATGGTCGTGAGATAAAAGCTGATAAGA-<br>TACCTCATAATAATAATGAAC<br>GGTCAGGTTATATAAAAGCCAGTAA-<br>TATAGCATCTGAAAAAAACATGGTTAATATCACCAGTGTTGCCGA<br>TGATATTAACAAGATTATTTCTTCAGTTTCAGGGTTCGAT-<br>TCAGGTGATGAACGATTAGCATCTTTATAT<br>AATTTATCCTTACATCAAAACAACACACATTCAACAACTTTAAC-<br>GACAACTGTCTGA<br>ATGACAGTAAATAAAATAAAGAACATTTTCAGCAATGCGACATCGAC-<br>TACAAAATCAGCATTTAATACAG<br>CATCATCAAACGTACGTACCGCTGGAAAAAAAATCATATTATTAATAC-<br>CTGATGATTATGAAGCTCAGGG<br>CGTGGGTATTAATGAGTTGGTCAAAGCTGCTGATGAGCTTGGAA-<br>TAGAAATACACCGTACTGAACGAGAT<br>GATACAGCGATTGCAAACCAGTTTTTTGGTACAGCAGAAAAAGTTTTAG-<br>GATTAACTGAACGTGGTGTTG |

CAATATTCGCACCACAACTTGATAAACTTCTGCAGAAGTATCAGAACGTT-GGGAGTAAAATAGGCGGAAC
CGCTGAGAATGTAGGTAATAATCTGGGAAAAGCCGGAACAGTTCTTTCAG-CACTACATAATTTTACGGGG
ATTGCCTTATCAGGCATGGCTCTTGATGAATTGCTGAGAAAACAACGGG-GAGGAGAGGATATAAGTCAGA
ATGATATTGCCAAAAGTAGTATTGAACTTATTAATCACCTTGTAGATA-CAGTATCAAGTATAAACAGTAC
TGTTGATTCATTTTCTGAGCAGCTTAACCAGCTTGGCTCATTTTTATCCAG-TAAACCTAGCTTAAGTTCT
GTTGGTGGAAAATTACAAAATTTACCTGACCTGGGCCCCTTGGGG-GATGGGCTGGATGTTATCTCTGGAA
TTCTTTCAGCTGTATCAGCAAGCTTTATTCTGGGAAACAGTGACGCACATA-CAGGAACAAAAGCAGCAGC
AGGTATCGAACTGACAACTCAGGTTCTGGGAAATGTTGGTAAA-GCTGTTTCACAATATATTCTGGCTCAG
AGAATGGCACAGGGCTTATCGACAACAGCTG-CAAGTGCGGGTCTGATCACATCGGCTGTTATGCTGGCTA
TCAGTCCTCTTTCTTTCCTGGCTATTGCAGATAAATTTGAGCGAGCTAA-GCAGCTTGAAGCATATTCTGA
ACGATTTAAAAAATTGAATTATGAAGGGGATGCTTTACTT-GCAGCCTTTCATAAAGAAACCGGAGCTATA
GATGCAGCCCTGACAACAATAAATACTGTTCTGAGTTCTGTATCTGCGG-GAGTTAGTGCAGCCTCCAGTG
CATCCCTCATAGGGGCCCCGATAAGCATGCTAGTGAGTGCATTAACCGG-TACGATATCTGGCATTCTGGA
AGCATCAAAACAGGCTATGTTTGAGCACGTTGCAGA-TAAATTCGCTGCTCGGATCAATGAATGGGAAAAG
GAGCATGGCAAAAATTATTTGAGAATGGCTATGATGCAAGACATGCTG-CATTTTTAGAAGACTCTCTGT
CTTTGCTTGCAGATTTTTCTCGTCAGCATGCAGTAGAAAGAGCTGTCGCAA-TAACTCAGCAACATTGGGA
TGAGAAGATCGGTGAACTTGCAGGTATAACCCGTAATGCTGATCGTAG-TCAGAGTGGTAAGGCATATATT
AATTATCTGGAGAATGGAGGGCTTTTAGAGGATCAACCGAAGGAG-TTTACACAACAAGTTTTTGATCCTC
AAAAAGGGACCATAGACCTTTCAACAGGTAATGTATCAAGTGTTTT-GACATTTGTAACACCAACATTTAC
CCCAGGAGAAGAAGTCAGAGAAAGAAAACAGAGTGGTAAA-TATGAATATATGACATCTCTTATTGTAAAT
GGTAAGGATACATGGACTGTAAAAGGATTAAAAAGTCATAAAGGTG-TATATGATTATTCAAATCTGATTC
AGTTTGTTGAAAAGGATAATAAATACCATCAGGCGAAAA-TAATTTCTGAGCTCGGAGATAAAAATGATAT
TGTTTATTCTGGCTCAGGCTCGTCAGAAGTATTTGCTGGA-GAAGGCCATGACACCGTATCTTATAATAAG
ACGGATGCTGGCAAACTAACAATTGATGCAACAGGAGCATCGAAAC-CTGGTGAGTATATAGTTTCAAAAA
ATATGTATGGTGACGTGAAGGTATTGCAGGAAGTCGTTAATGAACAG-GAGGTGTCAGTAGGAAAGCGAAC

| | | | | |
|---|---|---|---|---|
| EF204928.1 (AGR674) | ONT | D | Cattle and sheep | AGAGAAAATACAATATCGTGATTTTGAATTCAGAGCCGGTG-<br>GAATTTCTTATGATGTGATAGATAAGCTA<br>CATTCTGTTGAAGAACTCATCGGCG-<br>GAAAACATGATGATGAATTCAAAGGCGGTAAGTTTAATGATATAT<br>TCCATGGCGCAGATGGGAACGATTATATCGAAGGTAATTATGG-<br>TAATGATCGACTATACGGCGATGATGG<br>GGATGATTATATATCCGGAGGACAGGGCGACGACCAGTTATTTGGTGG-<br>TAGTGGAAACGATAAATTGAGT<br>GGAGGGGATGGTAATAATTATCTGACAGGAGGAAGCGG-<br>TAATGATGAGCTTCAGGCACATGGAGCTTATA<br>ATATTCTGTCAGGTGGCACTGGTGATGATAAACTTTATGGTGGTGG-<br>TATTGACCTTCTGGATGGAGGGGA<br>AGGTAATGACTATCTGAATGGCGGTTTTGGTAATGA-<br>TATTTATGTTTATCGGCAAAACTATGGTCATCAT<br>ACAATTGCAGATGAAGGAGGTAAAGGAGATCGCCTGCACTTATCTGA-<br>TATTAGCTTTGATGATATCGCAT<br>TTAAGAAAGTGGGAAATGATCTTATCATGAATAAAGCCATTAATGGTG-<br>TACTATCATTTAATGAGTCAAA<br>TGATGTCAATGGGATAACATTTAAAAACTGGTTTGCGAAAGATGCCTCAG-<br>GAGCAGATAATCATCTTGTT<br>GAGATTATAACAGATAAAGATGGTCGTGAAATTAAATCTGATAACAT-<br>ACCTCATAAAAATAATGATTGGT<br>CAGGTTATATAAAAGCTAGTTCTATAGCATCAGAAAAAAATATGGTTAA-<br>TATCACCAGTGTTGCCAATGA<br>TATTAACAAAATTATTTCTTCAGTTTCAGGGTTAGATTCAGGTGATGAAC-<br>GATTAGCATCTTTATATAAT<br>TTAGCCTTACATCAAAATAACACACACTCAACAACTTTAAC-<br>GACAACTGTCTGA<br>ATGACAGTAAATAAAATAAAGAACATTTTCAGCAATGCGACATCGAC-<br>TACAAAATCAGCATTTAATACAG<br>CATCATCAAACGTACGTACCGCTGGAAAAAAAATCATATTATTAATAC-<br>CTGATGATTATGAAGCTCAGGG<br>CGTGGGTATTAATGAGTTGGTCAAAGCTGCTGATGAGCTTGGAA-<br>TAGAAATACACCGTACTGAACGAGAT<br>GATACAGCGATTGCAAACCAGTTTTTGGTACAGCAGAAAAAGTTTTAG-<br>GATTAACTGAACGTGGTGTTG<br>CAATATTCGCACCACAACTTGATAAACTTCTGCAGAAGTATCAGAACGTT-<br>GGGAGTAAAATAGGCGGAAC<br>CGCTGAGAATGTAGGTAATAATCTGGGAAAAGCCGGAACAGTTCTTTCAG-<br>CACTACATAATTTTACGGGG<br>ATTGCCTTATCAGGCATGGCTCTTGATGAATTGCTGAGAAAACAACGGG-<br>GAGGAGAGGATATAAGTCAGA<br>ATGATATTGCCAAAAGTAGTATTGAACTTATTAATCACCTTGTAGATA-<br>CAGTATCAAGTATAAACAGTAC<br>TGTTGATTCATTTTCTGAGCAGCTTAACCAGCTTGGCTCATTTTTATCCAG-<br>TAAACCTAGCTTAAGTTCT<br>GTTGGTGGAAAATTACAAAATTTACCTGACCTGGGCCCCTTGGGG-<br>GATGGGCTGGATGTTATCTCTGGAA<br>TTCTTTCAGCTGTATCAGCAAGCTTTATTCTGGGAAACAGTGACGCACATA-<br>CAGGAACAAAAGCAGCAGC |

AGGTATCGAACTGACAACTCAGGTTCTGGGAAATGTTGGTAAA-
GCTGTTTCACAATATATTCTGGCTCAG
AGAATGGCACAGGGCTTATCGACAACAGCTG-
CAAGTGCGGGTCTGATCACATCGGCTGTTATGCTGGCTA
TCAGTCCTCTTTCTTTCCTGGCTATTGCAGATAAATTTGAGCGAGCTAA-
GCAGCTTGAAGCATATTCTGA
ACGATTTAAAAAATTGAATTATGAAGGGGATGCTTTACTT-
GCAGCCTTTCATAAAGAAACCGGAGCTATA
GATGCAGCCCTGACAACAATAAATACTGTTCTGAGTTCTGTATCTGCGG-
GAGTTAGTGCAGCCTCCAGTG
CATCCCTCATAGGGGCCCCGATAAGCATGCTAGTGAGTGCATTAACCGG-
TACGATATCTGGCATTCTGGA
AGCATCAAAACAGGCTATGTTTGAGCACGTTGCAGA-
TAAATTCGCTGCTCGGATCAATGAATGGGAAAAG
GAGCATGGCAAAAATTATTTGAGAATGGCTATGATGCAAGACATGCTG-
CATTTTTAGAAGACTCTCTGT
CTTTGCTTGCAGATTTTTCTCGTCAGCATGCAGTAGAAAGAGCTGTCGCAA-
TAACTCAGCAACATTGGGA
TGAGAAGATCGGTGAACTTGCAGGTATAACCCGTAATGCTGATCGTAG-
TCAGAGTGGTAAGGCATATATT
AATTATCTGGAGAATGGAGGGCTTTTAGAGGATCAACCGAAGGAG-
TTTACACAACAAGTTTTTGATCCTC
AAAAAGGGACCATAGACCTTTCAACAGGTAATGTATCAAGTGTTTT-
GACATTTGTAACACCAACATTTAC
CCCAGGAGAAGAAGTCAGAGAAAGAAAACAGAGTGGTAAA-
TATGAATATATGACATCTCTTATTGTAAAT
GGTAAGGATACATGGACTGTAAAAGGATTAAAAAGTCATAAAGGTG-
TATATGATTATTCAAATCTGATTC
AGTTTGTTGAAAAGGATAATAAATACCATCAGGCGAAAA-
TAATTTCTGAGCTCGGAGATAAAAATGATAT
TGTTTATTCTGGCTCAGGCTCGTCAGAAGTATTTGCTGGA-
GAAGGCCATGACACCGTATCTTATAATAAG
ACGGATGCTGGCAAACTAACAATTGATGCAACAGGAGCATCGAAAC-
CTGGTGAGTATATAGTTTCAAAAA
ATATGTATGGTGACGTGAAGGTATTGCAGGAAGTCGTTAATGAACAG-
GAGGTGTCAGTAGGAAAGCGAAC
AGAGAAAATACAATATCGTGATTTTGAATTCAGAGCCGGTG-
GAATTTCTTATGATGTGATAGATAAGCTA
CATTCTGTTGAAGAACTCATCGGCG-
GAAAACATGATGATGAATTCAAAGGCGGTAAGTTTAATGATATAT
TCCATGGCGCAGATGGGAACGATTATATCGAAGGTAATTATGG-
TAATGATCGACTATACGGCGATGATGG
GGATGATTATATATCCGGAGGACAGGGCGACGACCAGTTATTTGGTGG-
TAGTGGAAACGATAAATTGAGT
GGAGGGGATGGTAATAATTATCTGACAGGAGGAAGCGG-
TAATGATGAGCTTCAGGCACATGGAGCTTATA
ATATTCTGTCAGGTGGCACTGGTGATGATAAACTTTATGGTGGTGG-
TATTGACCTTCTGGATGGAGGGGA
AGGTAATGACTATCTGAATGGCGGTTTTGGTAATGA-
TATTTATGTTTATCGGCAAAACTATGGTCATCAT

| | | | | |
|---|---|---|---|---|
| EF2049<br>29.1<br>(ER03/<br>4238) | O157 | B | Cattle<br>and<br>sheep | ACAATTGCAGATGAAGGAGGTAAAGGAGATCGCCTGCACTTATCTGA-<br>TATTAGCTTTGATGATATCGCAT<br>TTAAGAAAGTGGGAAATGATCTTATCATGAATAAAGCCATTAATGGTG-<br>TACTATCATTTAATGAGTCAAA<br>TGATGTCAATGGGATAACATTTAAAAACTGGTTTGCGAAAGATGCCTCAG-<br>GAGCAGATAATCATCTTGTT<br>GAGATTATAACAGATAAAGATGGTCGTGAAATTAAATCTGATAACAT-<br>ACCTCATAAAAATAATGATTGGT<br>CAGGTTATATAAAAGCTAGTTCTATAGCATCAGAAAAAAATATGGTTAA-<br>TATCACCAGTGTTGCCAATGA<br>TATTAACAAAATTATTTCTTCAGTTTCAGGGTTAGATTCAGGTGATGAAC-<br>GATTAGCATCTTTATATAAT<br>TTAGCCTTACATCAAAATAACACACACTCAACAACTTTAACGACAC-<br>CTGTCTGA<br>ATGACAGTAAATAAAATAAAGAACATTTTCAATAATGCGACATTGAC-<br>TACAAAATCAGCATTTAATACAG<br>CATCATCAAGCGTACGTTCCGCTGGAAAAAAACTCATATTATTAATAC-<br>CTGATAATTATGAAGCTCAGGG<br>CGTGGGTATTAATGAGTTGGTCAAAGCTGCTGATGAGCTTGGAA-<br>TAGAAATACACCGTACTGAACGAGAT<br>GATACAGCGATTGCAAACCAGTTTTTGGTGCAGCAGAAAAAGTTGTAG-<br>GATTAACTGAACGTGGTGTTG<br>CAATATTCGCACCACAACTTGACAAACTTCTGCAGAAGTATCAGAAAGTT-<br>GGGAGTAAAATAGGAGGAAC<br>CGCTGAAAATGTAGGTAATAATCTGGGAAAAGCCGGAACAG-<br>TTCTCTCAGCACTACAGAATTTTACGGGG<br>ATTGCTTTATCAGGCATGGCTCTTGATGAATTGCTGAGAAAACAACGGG-<br>CAGGAGAGGATATAAGTCAGA<br>ATGATATTGCCAAAAGTAGTATTGAACTTATTAATCAGCTTGTAGATA-<br>CAGTATCAAGTATAAACAGTAC<br>CGTTGATTCATTTTCTGAGCAGCTTAACCAGCTTGGCTCATTTTTATCCAG-<br>TAAACCTCGATTAAGTTCT<br>GTTGGTGGGAAATTACAAAATTTACCAGACCTGGGCCCCCTGGGG-<br>GATGGGCTGGATGTTGTCTCCGGAA<br>TTCTTTCTGCTGTATCAGCAAGCTTTATTCTGGGAAACAGTGACGCACATA-<br>CAGGAACAAAAGCTGCAGC<br>GGGTATCGAACTGACAACTCAGGTTCTTGGAAATGTTGGTAAA-<br>GCTGTTTCGCAATATATTCTGGCTCAG<br>AGAATGGCACAGGGGTTATCGACAACAGCTG-<br>CAAGTGCGGGTCTGATCACATCGGCTGTTATGCTGGCTA<br>TCAGTCCTCTTTCTTTCCTGGCTGCTGCAGATAAATTTGAGCGAGCTAA-<br>GCAGCTTGAATCATATTCTGA<br>ACGATTTAAAAAATTGAATTATGAAGGGGATGCTTTACTCG-<br>CAGCCTTTCATAAAGAAACCGGAGCTATA<br>GATGCAGCCCTGACAACAATAAATACTGTCCTGAGTTCTGTATCTGCGG-<br>GAGTTAGTGCAGCCTCCAGTG<br>CATCCCTCATAGGGGCCCCGATAAGCATGCTGGTGAGTGCATTAACCGG-<br>TACGATATCTGGCATTCTGGA<br>AGCATCAAAACAGGCTATGTTTGAGCACGTTGCAGA-<br>GAAATTCGCTGCTCGGATCAATGAATGGGAAAAG |

GAGCATGGCAAAAATTATTTTGAGAATGGATATGACGCAAGA-
CATGCTGCGTTTTTAGAAGACTCTCTGT
CTTTGCTTGCTGATTTTTCTCGTCAGCATGCAGTAGAAAGAGCAGTCGCAA-
TAACCCAGCAACATTGGGA
TGAGAAGATCGGTGAACTTGCAGGCATAACCCGTAATGCTGATCGCAG-
TCAGAGTGGTAAGGCATATATT
AATTATCTGGAAAATGGAGGGCTTTTAGAGGCTCAACCGAAGGAG-
TTTACACAACAAGTGTTTGATCCTC
AAAAAGGGACCATAGACCTTTCAACAGGTAATGTATCAAGTGTTTT-
GACATTTATAACACCAACATTTAC
CCCAGGAGAAGAAGTTAGAGAAAGAAAACAGAGTGGTAAA-
TATGAATATATGACATCTCTTATTGTAAAT
GGTAAGGATACATGGTCTGTAAAAGGCATAAAAAATCATAAAGGTG-
TATATGATTATTCAAAATTGATTC
AGTTTGTTGAAAAGAATAACAAACACTATCAGGCGAGAA-
TAATTTCTGAGCTCGGAGATAAAGACGATGT
GGTTTATTCTGGAGCAGGCTCATCAGAAGTATTTGCTGGTGAAGGTTATGA-
TACCGTATCTTATAATAAG
ACGGATGTTGGTAAACTAACAATTGATGCAACAGGAGCATCAAAAC-
CTGGTGAGTATATAGTTTCAAAAA
ATATGTATGGTGACGTGAAGGTATTGCAGGAAGTCGTTAAGGAACAG-
GAGGTGTCAGTAGGGAAGCGAAC
AGAGAAAATACAATATCGTGATTTTGAATTCAGAACCGGTG-
GAATTCCTTATGATGTAATAGATAATCTT
CATTCTGTTGAAGAGCTCATTGGCG-
GAAAACATGATGATGAATTCAAAGGCGGTAAGTTTAATGATATAT
TCCATGGCGCAGATGGGAACGATTATATCGAAGGTAATTATGG-
TAATGATCGACTATACGGCGATGATGG
GGATGATTATATATCCGGAGGACAGGGAGACGACCAGTTATTTGGTGG-
TAGTGGAAACGATAAATTGAGT
GGAGGGGATGGTAATAATTATCTGACAGGAGGAAGCGG-
TAATGATGAGCTTCAGGCACACGGAGCTTATA
ATATTCTGTCAGGTGGTACTGGTGATGATAAACTTTATGGTGGTGGTGG-
TATTGATCTTCTGGATGGAGG
GGAAGGTAATGACTATCTGAATGGTGGTTTTGGTAATGA-
TATTTATGTTTATGGGCAAAACTATGGTCAT
CATACAATTGCAGATGAAGGAGGTAAAGGAGATCGTTTGCACTTATCTGA-
TATTAGCTTTGATGATATCG
CATTTAAGAGAGTTGGAAATGATCTTATCATGAATAAA-
GCCATTAATGGTGTACTTTCATTTAATGAGTC
AAATGATGTCAATGGGATAACATTTAAAAACTGGTTTGCGAAA-
GATGCCTCAGGAGCAGATAATCATCTT
GTTGAGGTTATAACAGATAAAGATGGTCGAGAGATAAAAGTTGATAAGA-
TACCTCATAATAATAATGAAC
GGTCAGGTTATATAAAAGCCAGTAA-
TATAGCATCTGAAAAAAACATGGTTAATATCACCAGTGTTGCCAA
TGATATTAATAAGATTATTTCTTCAGTTTCAGGGTTCGAT-
TCAGGTGATGAACGATTAGCATCTTTATAT
AATTTATCCTTACATCAAAACAACACACACTCAACAACTTTAAC-
GACAACTGTCTGA

| | | | | |
|---|---|---|---|---|
| CP0124 95.1 (CFSA N00417 7) | 145 | E | *Homo sapiens* | ATGACAGCAAATAAAATAAAGAGCATTTTCAATAATGCTGCATTGAC-<br>TACAAAATCAGCATTTAATACAG<br>CATCATCAAGCGTACGTTCCGCTGGAAAAAAACTCATATTATTAATAC-<br>CTGATAATTATGAAGCTCAGGG<br>CGTGGGTATTAATGAGTTGGTCAAAGCTGCTGATGAGCTTGGAA-<br>TAGAAATACACCGTACTGAACGAGAT<br>GATACAGCGATTGCAAACCAGTTTTTGGTACAGCAGAAAAAGTTGTAG-<br>GATTAACTGAACGTGGTGTTG<br>CAATATTCGCACCACAACTTGACAAACTTCTGCAGAAGTATCAGAAAGTT-<br>GGGAGTAAAATAGGAGGAAC<br>CGCTGAAAATGTAGGTAATAATCTGGGAAAAGCCGGAACAG-<br>TTCTCTCAGCACTACAGAATTTTACGGGG<br>ATTGCTTTATCAGGCATGGCTCTTGATGAATTGCTGAGAAAACAACGG-<br>GAAGGAGAGGATATAAGTCAGA<br>ATGATATTGCCAAAAGTAGTATTGAACTTATTAATCAACTTGTAGATA-<br>CAGTATCAAGTATAAACAGTAC<br>CGTTGATTCATTTTCTGAGCAGCTTAACCAGCTTGGCTCATTTTTATCCAG-<br>TAAACCTCGCTTAAGTTCT<br>GTTGGTGGAAAATTACAAAATTTACCGGACCTGGGCCCCTTGGGG-<br>GATGGGCTGGATGTTGTCTCCGGAA<br>TTCTTTCTGCTGTATCAGCAAGCTTTATTCTGGGAAACAGTGACGCACATA-<br>CAGGAACAAAAGCTGCAGC<br>GGGTATCGAACTGACAACTCAGGTTCTTGGAAATGTTGGTAAA-<br>GCTGTTTCGCAATATATTCTGGCTCAG<br>AGAATGGCACAGGGGTTATCGACAACAGCTG-<br>CAAGTGCGGGTCTGATCACATCGGCTGTTATGCTGGCTA<br>TCAGTCCTCTTTCTTTCCTGGCTATTGCAGATAAATTTGAGCGAGCTAA-<br>GCAGCTTGAATCATATTCTGA<br>ACGATTTAAAAAATTGAATTATGAAGGGGATGCTTTACTCG-<br>CAGCCTTTCATAAAGAAACCGGAGCTATA<br>GATGCAGCCCTGACAACAATAAATACTGTCCTGAGTTCTGTATCTGCGG-<br>GAGTTAGTGCAGCCTCCAGTG<br>CATCCCTCATAGGGGCCCCGATAAGCATGCTGGTGAGTGCATTAACCGG-<br>TACGATATCTGGCATTCTGGA<br>AGCATCAAAACAGGCTATGTTTGAGCACGTTGCAGA-<br>TAAATTCGCTGCTCGGATCAATGAATGGGAAAAG<br>GAGCATGGCAAAAATTATTTTGAGAATGGCTATGACGCAAGA-<br>CATGCTGCGTTTTTAGAAGATTCTCTGT<br>CTTTGCTTGCTGATTTTTCTCGTCAGCATGCAGTAGAAAGAGCTGTCGCAA-<br>TAACCCAGCAACATTGGGA<br>TGAGAAGATCGGTGAACTTGCAGGTATAACCCGTAATGCTGATCGCAG-<br>TCAGAGTGGTAAGGCATATATT<br>AATTATCTGGAGAATGGAGGGCTTTTAGCGGCTCAACCGAAGGAG-<br>TTTACACAACAAGTTTTTGATCCTC<br>AAAAAGGGACTATAGACCTTTCAACAGGTAATGTATCAAGTGTTTT-<br>GACATTTGTAACACCAACATTTAC<br>CCCAGGAGAAGAAGTCAGAGAAAGAAAACAGAGTGGTAAA-<br>TATGAATATATGACATCTCTTATTGTAAAT<br>GGTAAGGATACATGGTCTGTAAAAGGCATAAAAAATCATAAAGGTG-<br>TATATGATTATTCAAATTTGATTC |

| | | | | |
|---|---|---|---|---|
| CP012499.1 (GB089) | O168 | D | Ground beef | AGTTTGTTGAAAAGGATAACAAACACTATCAGGCGAGAA- TAATTTCTGAGCTCGGAGATAAAGACGATAT AGTTTATTCTGGGGCTGGCTCATCAGAAGTATTTGCTGGTGAAGGCCATGA- TACCGTATCTTATAATAAG ACGGATGTTGGTAAACTAACAATTAATGCAACAGGAGCATCAAAAC- CTGGTGAATATATAGTTTCAAAAA ATATGTATGGTGACGTGAAGGTATTGCAGGAAGTCGTTAAGGAACAG- GAGGTGTCAGTAGGGAAGCGAAC AGAGAAAATACAATATCGTGATTTTGAATTCAGAACCGGTG- GAATTCCTTATGATGTGATAGATAATCTT CATTCTGTTGAAGAACTCATTGGCG- GAAAACATGATGATGAATTCAAAGGCGGTAAGTTTAATGATATAT TCCATGGCGCAGATGGGAACGATTATATCGAAGGTAATTATGG- TAATGATCGACTATACGGCGATGATGG GGATGATTATATATCCGGAGGACAGGGAGACGACCAGTTATTTGGTGG- TAGTGGAAACGATAAATTGAGT GGAGGGGATGGTAATAATTATCTGACAGGAGGAAGCGG- TAATGATGAGCTTCAGGCACACGGGGCTTATA ATATTCTGTCAGGTGGTACTGGTGATGATAAACTTTATGGTGGTGGTGG- TATTGACCTTCTGGATGGAGG GGAAGGTAATGACTATCTGAATGGTGGTTTTGGTAATGA- TATTTATGTTTATAGGCAAAACTATGGTCAT CATACAATTGCAGATGAAGGAGGTAAAGGAGATCGTCTGCACTTATCTGA- TATTAGCTTTGATGATATCG CATTTAAGAGAGTTGGAAATGATCTTATCATGAATAAA- GCCATTAATGGTGTACTTTCATTTAATGAGTC AAATGATGTCAATGGGATAACATTTAAAAACTGGTTTGAGAAA- GATGCCTCAGGAGCAGATAATCATCTT GTTGAGGTTATAACAGATAAAGATGGTCGTGAGATAAAAGCTGATAAGA- TACCTCATAATAATAATGAAC GGTCAGGTTATATAAAAGCCAGTAA- TATAGCATCTGAAAAAAACATGGTTAATATCACCAGTGTTGCCGA TGATATTAACAAGATTATTTCTTCAGTTTCAGGGTTCGAT- TCAGGTGATGAACGATTAGCATCTTTATAT AATTTATCTTTACATCAAAACAACACACACTCAACAACTTTAAC- GACAACTGTCTGA ATGACAGTAAATAAAATAAAGAACATTTTCAGCAATGCGACATCGAC- TACAAAATCAG- CATTTAATACAGCATCATCAAACGTACGTACCGCTGGAAAAAAACTCATA TTATTAATACCTGATGATTATGAAGCTCAGGGCGTGGGTATTAATGAGTT- GGTCAAA- GCTGCTGATGAGCTTGGAATAGAAATACACCGTACTGAACGAGATGATAC AGCGATTGCAAACCAGTTTTTTGGTACAGCAGAAAAAGTTTTAGGAT- TAACTGAAC- GTGGTGTTGCAATATTCGCACCACAACTTGATAAACTTCTGCAGAAGTATC AGAACGTTGGGAGTAAAATAGGCGGAACCGCTGAGAATGTAGGTAA- TAATCTGGGAAAA- GCCGGAACAGTTCTTTCAGCACTACATAATTTTACGGGGATTGCCTTATCA GGCATGGCTCTTGATGAATTGCTGAGAAAACAACGGGGAGGAGAGGATA- TAAGTCAGAATGA- TATTGCCAAAAGTAGTATTGAACTTATTAATCACCTTGTAGATACAGTATC |

AAGTATAAACAGTACTGTTGATTCATTTTCTGAGCAGCTTAACCAGCTT-
GGCTCATTTTTATCCAGTAAACCTAGCTTAAGTTCTGTTGGTGGAAAATTAC
AAAATTTACCTGACCTGGGCCCCTTGGGGGATGGGCTGGATGTTATCTCTG-
GAATTCTTTCAGCTGTATCAGCAAGCTTTATTCTGGGAAACAGTGACGCAC
ATACAGGAACAAAAGCAGCAG-
CAGGTATCGAACTGACAACTCAGGTTCTGGGAAATGTTGG-
TAAAGCTGTTTCACAATATATTCTGGCTCAGAGAATGGCACAGGGCTTATC
GACAACAGCTGCAAGTGCGGGTCTGATCACATCGGCTGTTATGCTGGC-
TATCAG-
TCCTCTTTCTTTCCTGGCTATTGCAGATAAATTTGAGCGAGCTAAGCAGCTT
GAAGCATATTCTGAACGATTTAAAAAATTGAATTATGAAGGG-
GATGCTTTACTT-
GCAGCCTTTCATAAAGAAACCGGAGCTATAGATGCAGCCCTGACAACAAT
AAATACTGTTCTGAGTTCTGTATCTGCGGGAGTTAGTGCAGCCTCCAGTG-
CATCCCTCATAGGGGCCCCGATAAGCATGCTAGTGAGTGCATTAACCGGT
ACGATATCTGGCATTCTGGAAGCATCAAAACAGGCTATGTTTGAGCAC-
GTTGCAGA-
TAAATTCGCTGCTCGGATCAATGAATGGGAAAAGGAGCATGGCAAAAATT
ATTTTGAGAATGGCTATGATGCAAGACATGCTGCATTTTTAGAA-
GACTCTCTGTCTTTGCTT-
GCAGATTTTTCTCGTCAGCATGCAGTAGAAAGAGCTGTCGCAATAACTCA
GCAACATTGGGATGAGAAGATCGGTGAACTTGCAGGTATAACCCG-
TAATGCTGATCGTAG-
TCAGAGTGGTAAGGCATATATTAATTATCTGGAGAATGGAGGGCTTTTAGA
GGATCAACCGAAGGAGTTTACACAACAAGTTTTT-
GATCCTCAAAAAGGGACCATAGAC-
CTTTCAACAGGTAATGTATCAAGTGTTTTGACATTTGTAACACCAACATTT
ACCCCAGGAGAAGAAGTCAGAGAAAGAAAACAGAGTGGTAAA-
TATGAATATATGACATCTCTTATTGTAAATGGTAAGGATACATGGTCTGTA
AAAGGATTAAAAAGTCATAAAGGTGTATATGATTATTCAAATCTGAT-
TCAGTTTGTT-
GAAAAGGATAATAAATACCATCAGGCGAAAATAATTTCTGAGCTCGGAG
ATAAAAATGATATTGTTTATTCTGGCTCAGGCTCGTCAGAAGTATTTGCTG-
GA-
GAAGGCCATGACACCGTATCTTATAATAAGACGGATGCTGGCAAACTAAC
AATTGATGCAACAGGAGCATCGAAACCTGGTGAGTATA-
TAGTTTCAAAAAA-
TATGTATGGTGACGTGAAGGTATTGCAGGAAGTCGTTAATGAACAGGAGG
TGTCAGTGGGAAAGCGAACAGAGAAAATACAATATCGTGATTTT-
GAATTCAGAGCCGGTG-
GAATTTCTTATGATGTGATAGATAAGCTACATTCTGTTGAAGAACTCATCG
GCGGAAAACATGATGATGAATTCAAAGGCGGTAAGTTTAATGATA-
TATTCCATGGCG-
CAGATGGGAACGATTATATCGAAGGTAATTATGGTAATGATCGACTATAC
GGCGATGATGGGGATGATTATATATCCGGAGGACAGGGCGACGACCAG-
TTATTTGGTGGTAG-
TGGAAACGATAAATTGAGTGGAGGGGATGGTAATAATTATCTGACAGGAG
GAAGCGGTAATGATGAGCTTCAGGCACACGGAGCTTATAA-
TATTCTGTCAGGTGGCAC-
TGGTGATGATAAACTTTATGGTGGTGGTATTGACCTTCTGGATGGAGGGGA

| | | | | |
|---|---|---|---|---|
| NZ_CP006263.1 (RM13516) | 145 | C | Homo sapiens | AGGTAATGACTATCTGAATGGCGGTTTTGGTAATGA-<br>TATTTATGTTTATCGGCAAAAC-<br>TATGGTCATCATACAATTGCAGATGAAGGAGGTAAAGGAGATCGCCTGCA<br>CTTATCTGATATTAGCTTTGATGATATCGCATTTAAGAAAGTGG-<br>GAAATGATCTTATCATGAATAAAGCCATTAATGGTGTAATTTCATTTAATG<br>AGTCAAATGATGTCAATGGGATAACATTTAAAAACTGGTTTGCGAAA-<br>GATGCCTCAGGAG-<br>CAGATAATCATCTTGTTGAGATTATAACAGATAAAGATGGTCGTGAAATTA<br>AATCTGATAACATACCTCATAAAAATAATGATTGGTCAGGTTATATAAAA-<br>GCTAGTTC-<br>TATAGCATCAGAAAAAAATATGGTTAATATCACCAGTGTTGCCAATGATA<br>TTAACAAAATTATTTCTTCAGTTTCAGGGTTAGATTCAGGTGATGAAC-<br>GATTAGCATCTTTA-<br>TATAATTTAGCCTTACATCAAAATAACACACACTCAACAATTTTAACGACA<br>ACTGTCTGA<br>ATGACAGTAAATAAAATAAAGAACATTTTCAATAATGCGACATTGAC-<br>TACAAAATCAGCATTTAATACAG<br>CATCATCAAGCGTACGTTCCGCTGGCAAAAAACTCATATTATTAATAC-<br>CTGATAATTATGAAGCTCAGGG<br>CGTGGGTATTAATGAGTTGGTCAAAGCTGCTGATGAGCTTGGAA-<br>TAGAAATACACCGTACTGAACGAGAT<br>GATACAGCGATTGCAAACCAGTTTTTGGTGCAGCAGAAAAAGTTGTAG-<br>GATTAACTGAACGTGGTGTTG<br>CAATATTCGCACCACAACTTGACAAACTTCTGCAGAAGTATCAGAAAGTT-<br>GGGAGTAAAATAGGAAGAAC<br>CGCTGAAAATGTAGGTAATAATCTGGGAAAAGCCGGAACAG-<br>TTCTCTCAGCACTACAGAATTTTACGGGG<br>ATTGCTTTATCAGGCATGGCTCTTGATGAATTGCTGAGAAAACAACGG-<br>GAAGGAGAGGATATAAGTCAGA<br>ATGATATTGCCAAAAGTAGTATTGAACTTATTAATCAGCTTGTAGATA-<br>CAGTATCAAGTATAAACAGTAC<br>CGTTGATTCATTTTCTGAGCAGCTTAACCAGCTTGGCTCATTTTTATCCAG-<br>TAAACCTCGCTTAAGTTCT<br>GTTGGTGGGAAATTACAAAATTTACCAGACCTGGGCTCCCTGGGG-<br>GATGGGCTGGATGTTGTCTCCGGAA<br>TTCTTTCTGCTGTATCAGCAAGCTTTATTCTGGGAAACAGTGACGCACATA-<br>CAGGAACAAAAGCTGCAGC<br>GGGTATCGAACTGACAACTCAGGTTCTTGGAAATGTTGGTAAA-<br>GCTGTTTCGCAATATATTCTGGCTCAG<br>AGAATGGCACAGGGGTTATCGACAACAGCTG-<br>CAAGTGCGGGTCTGATCACATCGGCTGTTATGCTGGCTA<br>TCAGTCCTCTTTCTTTCCTGGCTGCTGCAGATAAATTTGAGCGAGCTAA-<br>GCAGCTTGAATCATATTCTGA<br>ACGATTTAAAAAATTGAATTATGAAGGGGATGCTTTACTCG-<br>CAGCCTTTCATAAAGAAACCGGAGCTATA<br>GATGCAGCCCTGACAACAATAAATACTGTCCTGAGTTCTGTATCTGCGG-<br>GAGTTAGTGCAGCCTCCAGTG<br>CATCCCTCATAGGGGCCCCGATAAGCATGCTGGTGAGTGCATTAACCGG-<br>TACGATATCTGGCATTCTGGA<br>AGCATCAAAACAGGCTATGTTTGAGCACGTTGCAGA-<br>GAAATTCGCTGCTCGGATCAATGAATGGGAAAAG |

GAGCATGGCAAAAATTATTTTGAGAATGGCTATGACGCAAGA-
CATGCTGCGTTTTTAGAAGACTCTCTGT
CTTTGCTTGCTGATTTTTCTCGTCAGCATGCAGTAGAAAGAGCAGTCGCAA-
TAACCCAGCAACATTGGGA
TGAGAAGATCGGTGAACTTGCAGGCATAACCCGTAATGCTGATCGCAG-
TCAGAGTGGTAAGGCATATATT
AATTATCTGGAAAATGGAGGGCTTTTAGAGGCTCAACCGAAGGAG-
TTTACACAACAAGTGTTTGATCCTC
AAAAAGGGACCATAGACCTTTCAACAGGTAATGTATCAAGTGTTTT-
GACATTTATAACACCAACATTTAC
CCCAGGAGAAGAAGTTAGAGAAAGAAAACAGAGTGGTAAA-
TATGAATATATGACATCTCTTATTGTAAAT
GGTAAGGATACATGGTCTGTAAAAGGCATAAAAAATCATAAAGGTG-
TATATGATTATTCAAAATTGATTC
AGTTTGTTGAAAAGAATAACAAACACTATCAGGCGAGAATGAT-
TTCTGAACTCGGAGATAAAGACGATGT
GGTTTATTCTGGAGCAGGCTCATCAGAAGTATTT-
GCTGGTGAAGGTCATGATACCGTATCTTATAATAAG
ACGGATGTTGGTAAACTAACAATTGATGCAACAGGAGCATCAAAAC-
CTGGTGAGTATATAGTTTCAAAAA
ATATGTATGGTGACGTGAAGGTATTGCAGGAAGTCGTTAAGGAACAG-
GAGGTGTCAGTAGGGAAGCGAAC
AGAGAAAATACAATATCGTGATTTTGAATTCAGAACCGGTG-
GAATTCCTTATGATGTAATAGATAATCTT
CATTCTGTTGAAGAGCTCATTGGCG-
GAAAACATGATGATGAATTCAAAGGCGGTAAGTTTAATGATATAT
TCCATGGCGCAGATGGGAACGATTATATCGAAGGTAATTATGG-
TAATGATCGACTATACGGCGATGATGG
GGATGATTATATATCCGGAGGACAGGGAGACGACCAGTTATTTGGTGG-
TAGTGGAAACGATAAATTGAGT
GGAGGGGATGGTAATAATTATCTGACAGGAGGAAGCGG-
TAATGATGAGCTTCAGGCACACGGAGCTTATA
ATATTCTGTCAGGTGGTACTGGTGATGATAAACTTTATGGTGGTGGTGG-
TATTGATCTTCTGGATGGAGG
GGAAGGTAATGACTATCTGAATGGTGGTTTTGGTAATGA-
TATTTATGTTTATAGGCAAAACTATGGTCAT
CATACAATTGCAGATGAAGGAGGTAAAGGAGATCGTTTGCACTTATCTGA-
TATTAGCTTTGATGATATCG
CATTTAAGAGAGTTGGAAATGATCTTATCATGAATAAA-
GCCATTAATGGTGTACTTTCATTTAATGAGTC
AAATGATGTCAATGGGATAACATTTAAAAACTGGTTTGCGAAA-
GATGCCTCAGGAGCAGATAATCATCTT
GTTGAGGTTATAACAGATAAAGATGGTCGAGAGATAAAAGTTGATAAAA-
TACCTCATAATAATAATGAAC
GGTCAGGTTATATAAAAGCCAGTAA-
TATAGCATCTGAAAAAAACATGGTTAATATCACCAGTGTTGCCAA
TGATATTAATAAGATTATTTCTTCAGTTTCAGGGTTCGAT-
TCAGGTGATGAACGATTAGCATCTTTATAT
AATTTATCCTTACATCAAAACAACACACACTCAACAACTTTAAC-
GACAACTGTCTGA

| | | | | |
|---|---|---|---|---|
| EF204926.1 (AGR374) | O5 | F | Cattle and sheep | ATGACAGTAAATAAAATAAAGAACATTTTCAATAATGCGACATTGAC-<br>TACAAAATCAGCATTTAATACAG<br>CATCATCAAGCGTACGTTCCGCTGGCAAAAAACTCATATTATTAATAC-<br>CTGATAATTATGAAGCTCAGGG<br>CGTGGGTATTAATGAGTTGGTCAAAGCTGCTGATGAGCTTGGAA-<br>TAGAAATACACCGTACTGAACGAGAT<br>GATACAGCGATTGCAAACCAGTTTTTGGTGCAGCAGAAAAAGTTGTAG-<br>GATTAACTGAACGTGGTGTTG<br>CAATATTCGCACCACAACTTGACAAACTTCTGCAGAAGTATCAGAAAGTT-<br>GGGAGTAAAATAGGAGGAAC<br>CGCTGAAAATGTAGGTAATAATCTGGGAAAAGCCGGAACAG-<br>TTCTCTCAGCACTACAGAATTTTACGGGG<br>ATTGCTTTATCAGGCATGGCTCTTGATGAATTGCTGAGAAAACAACGG-<br>GAAGGAGAGGATATAAGTCAGA<br>ATGATATTGCCAAAAGTAGTATTGAACTTATTAATCAGCTTGTAGATA-<br>CAGTATCAAGTATAAACAGTAC<br>CGTTGATTCATTTTCTGAGCAGCTTAACCAGCTTGGCTCATTTTTATCCAG-<br>TAAACCTCGCTTAAGTTCT<br>GTTGGTGGGAAATTACAAAATTTACCAGACCTGGGCTCCCTGGGG-<br>GATGGGCTGGATGTTGTCTCCGGAA<br>TTCTTTCTGCTGTATCAGCAAGCTTTATTCTGGGAAACAGTGACGCACATA-<br>CAGGAACAAAAGCTGCAGC<br>GGGTATAGAACTGACAACTCAGGTTCTTGGAAATGTTGGTAAA-<br>GCTGTTTCGCAATATATTCTGGCTCAG<br>AGAATGGCACAGGGGTTATCGACAACAGCTG-<br>CAAGTGCGGGTCTGATCACATCGGCTGTTATGCTGGCTA<br>TCAGTCCTCTTTCTTTCCTGGCTGCTGCAGATAAATTTGAGCGAGCTAA-<br>GCAGCTTGAATCATATTCTGA<br>ACGATTTAAAAAATTGAATTATGAAGGGGATGCTTTACTCG-<br>CAGCCTTTCATAAAGAAACCGGAGCTATA<br>GATGCAGCCCTGACAACAATAAATACTGTCCTGAGTTCTGTATCTGCGG-<br>GAGTTAGTGCAGCCTCCAGTG<br>CATCCCTCATAGGGGCCCCGATAAGCATGCTGGTGAGTGCATTAACCGG-<br>TACGATATCTGGCATTCTGGA<br>AGCATCAAAACAGGCTATGTTTGAGCACGTTGCAGA-<br>GAAATTCGCTGCTCGGATCAATGAATGGGAAAAG<br>GAGCATGGCAAAAATTATTTTGAGAATGGCTATGACGCAAGA-<br>CATGCTGCGTTTTTAGAAGACTCTCTGT<br>CTTTGCTTGCTGATTTTTCTCGTCAGCATGCAGTAGAAAGAGCAGTCGCAA-<br>TAACCCAGCAACATTGGGA<br>TGAGAAGATCGGTGAACTTGCAGGCATAACCCGTAATGCTGATCGCAG-<br>TCAGAGTGGTAAGGCATATATT<br>AATTATCTGGAAAATGGAGGGCTTTTAGAGGCTCAACCGAAGGAG-<br>TTTACACAACAAGTGTTTGATCCTC<br>AAAAAGGGACCATAGACCTTTCAACAGGTAATGTATCAAGTGTTTT-<br>GACATTTATAACACCAACATTTAC<br>CCCAGGAGAAGAAGTTAGAGAAAGAAAACAGAGTGGTAAA-<br>TATGAATATATGACATCTCTTATTGTAAAT<br>GGTAAGGATACATGGTCTGTAAAAGGCATAAAAAATCATAAAGGTG-<br>TATATGATTATTCAAAATTGATTC |

AGTTTGTTGAAAAGAATAACAAACACTATCAGGCGAGAA-
TAATTTCTGAGCTCGGAGATAAAGACGATGT
GGTTTATTCTGGAGCAGGCTCATCAGAAGTATTT-
GCTGGTGAAGGTCATGATACCGTATCTTATAATAAG
ACGGATGTTGGTAAACTAACAATTGATGCAACAGGAGCATCAAAAC-
CTGGTGAGTATATAGTTTCAAAAA
ATATGTATGGTGACGTGAAGGTATTGCAGGAAGTCGTTAAGGAACAG-
GAGGTGTCAGTAGGGAAGCGAAC
AGAGAAAATACAATATCGTGATTTTGAATTCAGAACCGGTG-
GAATTCCTTATGATGTAATAGATAATCTT
CATTCTGTTGAAGAGCTCATTGGCG-
GAAAACATGATGATGAATTCAAAGGCGGTAAGTTTAATGATATAT
TCCATGGCGCAGATGGGAACGATTATATCGAAGGTAATTATGG-
TAATGATCGACTATACGGCGATGATGG
GGATGATTATATATCCGGAGGACAGGGAGACGACCAGTTATTTGGTGG-
TAGTGGAAACGATAAATTGAGT
GGAGGGGATGGTAATAATTATCTGACAGGAGGAAGCGG-
TAATGATGAGCTTCAGGCACACGGAGCTTATA
ATATTCTGTCAGGTGGTACTGGTGATGATAAACTTTATGGTGGTGGTGG-
TATTGATCTTCTGGATGGAGG
GGAAGGTAATGACTATCTGAATGGTGGTTTTGGTAATGA-
TATTTATGTTTATAGGCAAAACTATGGTCAT
CATACAATTGCAGATGAAGGAGGTAAAGGAGATCGTTTGCACTTATCTGA-
TATTAGCTTTGATGATATCG
CATTTAAGAGAGTTGGAAATGATCTTATCATGAA-
TAAACCCATTAATGGTGTACTTTCATTTAATGAGTC
AAATGATGTCAATGGGATAACATTTAAAAACTGGTTTGCGAAA-
GATGCCTCAGGAGCAGATAATCATCTT
GTTGAGGTTATAACAGATAAAGATGGTCGAGAGATAAAAGTTGATAAAA-
TACCTCATAATAATAATGAAC
GGTCAGGTTATATAAAAGCCAGTAA-
TATAGCATCTGAAAAAAACATGGTTAATATCACCAGTGTTGCCAA
TGATATTAATAAGATTATTTCTTCAG-
TTTCAGGGTTCGATCCAGGTGATGAACGATTAGCATCTTTATAT
AATTTATCCTTACATCAAAACAACACACACTCAACAACTTTAAC-
GACAACTGTCTGA
